# Gut microbiome composition in the Hispanic Community Health Study/Study of Latinos is shaped by geographic relocation, environmental factors, and obesity

**DOI:** 10.1186/s13059-019-1831-z

**Published:** 2019-11-01

**Authors:** Robert C. Kaplan, Zheng Wang, Mykhaylo Usyk, Daniela Sotres-Alvarez, Martha L. Daviglus, Neil Schneiderman, Gregory A. Talavera, Marc D. Gellman, Bharat Thyagarajan, Jee-Young Moon, Yoshiki Vázquez-Baeza, Daniel McDonald, Jessica S. Williams-Nguyen, Michael C. Wu, Kari E. North, Justin Shaffer, Christopher C. Sollecito, Qibin Qi, Carmen R. Isasi, Tao Wang, Rob Knight, Robert D. Burk

**Affiliations:** 1 Department of Epidemiology and Population Health, Albert Einstein College of Medicine, 1300 Morris Park Avenue, Bronx, NY 10461 USA; 2 Division of Public Health Sciences, Fred Hutchinson Cancer Research Center, Seattle, WA USA; 3 Department of Pediatrics, Albert Einstein College of Medicine, Bronx, NY USA; 4 Department of Biostatistics, Gillings School of Global Public Health, University of North Carolina, Chapel Hill, NC USA; 5 Institute for Minority Health Research, University of Illinois at Chicago College of Medicine, Chicago, IL USA; 6 Department of Psychology, University of Miami, Miami, FL USA; 7 Division of Health Promotion and Behavioral Science, San Diego State University, San Diego, CA USA; 8 Division of Molecular Pathology and Genomics, University of Minnesota, Minneapolis, MN USA; 9 Jacobs School of Engineering, University of California, San Diego, La Jolla, CA USA; 10 Center for Microbiome Innovation, University of California, San Diego, La Jolla, CA USA; 11 Department of Pediatrics, University of California, San Diego, La Jolla, CA USA; 12 Department of Epidemiology, Gillings School of Global Public Health, University of North Carolina, Chapel Hill, NC USA; 13 Department of Computer Science and Engineering, University of California, San Diego, La Jolla, CA USA; 14 Department of Bioengineering, University of California, San Diego, La Jolla, CA USA; 15 Department of Microbiology & Immunology, Albert Einstein College of Medicine, Bronx, NY USA

**Keywords:** Microbiome, Epidemiology, Hispanic population, Mycobiome, Obesity

## Abstract

**Background:**

Hispanics living in the USA may have unrecognized potential birthplace and lifestyle influences on the gut microbiome. We report a cross-sectional analysis of 1674 participants from four centers of the Hispanic Community Health Study/Study of Latinos (HCHS/SOL), aged 18 to 74 years old at recruitment.

**Results:**

Amplicon sequencing of 16S rRNA gene V4 and fungal ITS1 fragments from self-collected stool samples indicate that the host microbiome is determined by sociodemographic and migration-related variables. Those who relocate from Latin America to the USA at an early age have reductions in *Prevotella* to *Bacteroides* ratios that persist across the life course. Shannon index of alpha diversity in fungi and bacteria is low in those who relocate to the USA in early life. In contrast, those who relocate to the USA during adulthood, over 45 years old, have high bacterial and fungal diversity and high *Prevotella* to *Bacteroides* ratios, compared to USA-born and childhood arrivals. Low bacterial diversity is associated in turn with obesity. Contrasting with prior studies, our study of the Latino population shows increasing *Prevotella* to *Bacteroides* ratio with greater obesity. Taxa within Acidaminococcus, Megasphaera, Ruminococcaceae, Coriobacteriaceae, Clostridiales, Christensenellaceae, YS2 (Cyanobacteria), and Victivallaceae are significantly associated with both obesity and earlier exposure to the USA, while Oscillospira and Anaerotruncus show paradoxical associations with both obesity and late-life introduction to the USA.

**Conclusions:**

Our analysis of the gut microbiome of Latinos demonstrates unique features that might be responsible for health disparities affecting Hispanics living in the USA.

Immigrants from Latin America and the Spanish-speaking Caribbean make up the majority of the foreign-born population living in the USA. Immigration-related life course experiences may affect the gut microbiome (GMB) among Latinos, with potential implications for chronic diseases that have been linked to the GMB [1]. Many of these, including obesity, diabetes, and asthma, are highly prevalent in the US Hispanic population [2, 3] although the association of these diseases with the Hispanic GMB pattern is unknown.

Migration from lower-income countries to higher-income countries is associated with change in community structure of the GMB due to adoption of a Western style diet, exposure to new natural and built environments, and other influences [4]. Follow-up studies of migrants suggest that geographic relocation to the USA often coincides with a decrease in gut microbial diversity and transition in GMB organisms, concurrent with replacement of dietary starches and fiber with animal proteins and fats [4]. Changes in diet alter the GMB makeup by restricting nutrients needed for growth of certain bacteria while enhancing the growth of others. After an altered GMB is established, the new microbial communities in the host gastrointestinal tract can lead to changes in metabolic processes and generation of metabolites [5, 6].

Hispanic/Latino groups, which include the largest immigrant population in the USA, are known to harbor a distinct GMB as compared with non-Hispanics [7], but this has only been studied in small, local populations [8]. Longitudinal assessments among migrants (e.g., Thailand to USA) [9] have extended over weeks to months and are consistent with geographic variation in GMB shown in cross-national comparisons between lower- and higher-income countries [9]. Lacking are large and detailed multicenter US Hispanic cohorts which can estimate effects of immigration on the GMB over the life course and inform about disease associations which may differ among populations [10]. Furthermore, such knowledge has the potential to facilitate the development of therapeutic interventions to alter the microbiome and treat or prevent disease.

We used data from a longstanding multicenter US cohort study to characterize the association of relocation to the mainland USA with GMB characteristics among individuals from several Latin American national backgrounds.

## Results

### Population characteristics

Among the participating group of 1674 Hispanic US residents (Table 1), approximately half were of Mexican/Mexican American background, while Puerto Ricans and Cubans each comprised over 10% of the population. Thirteen percent had been born in the mainland USA, almost all of whom were “second-generation” offspring of at least one Latin American-born parent. Fourteen percent were first-generation individuals who had relocated to the US mainland during childhood and adolescence (“relocation age” < 18 years old), whom we considered to be the “1.5 generation”. The remaining three quarters of the population had relocated from Latin America to the US mainland during adulthood. Puerto Rican-born individuals had the youngest relocation age (mean 18.6 years, standard deviation 12.1 years) and Cuban-born migrants had the oldest relocation age (mean 41.4 years, standard deviation 14.5 years) (Additional file 1: Figure S1). Peak decade of relocation ranged from the 1970s for Puerto Ricans to the 2000s for Cubans and Central and South Americans (Additional file 1: Figure S2).

**Table 1:** Demographic, behavioral, and socioeconomic variables, by birthplace and age at relocation to the mainland USA

	Mainland US born	Relocated 0–17 years old	Relocated 18–34 years old	Relocated 35–44 years old	Relocated 45+ years old
*N*	225	235	730	306	261
Age in years, mean	48.3 (13.8)	54.7 (12.1)	56.6 (9.8)	59.2 (7.6)	65.7 (6.6)
Sex (women)	58%	64%	65%	66%	65%
Height, cm	164.0 (8.9)	160.6 (8.7)	159.5 (8.6)	159.7 (8.6)	159.2 (9.5)
Education level < 9th grade	6%	22%	34%	30%	32%
Education level > high school	61%	38%	32%	45%	45%
Household income > $30K/year	56%	46%	38%	35%	26%
Mother’s education > high school	14%	7%	8%	7%	7%
Father’s education > high school	10%	11%	10%	10%	11%
English language preference	76%	34%	2%	1%	0.40%
Median year of relocation to USA	–	1970	1984	1996	2003
Childhood economic hardship*	55%	48%	57%	55%	53%
Childhood sanitation†	99%	88%	76%	85%	74%
Return to home country in last year	35%	34%	50%	47%	50%
SASH social relations subscale	2.6 (0.5)	2.4 (0.6)	2.2 (0.6)	2.0 (0.6)	1.9 (0.5)
Hispanic diet habits (dietary acculturation) ‡	40%	61%	82%	90%	89%
AHEI score, mean	47.8 (8.2)	49.9 (7.7)	52.5 (7.1)	52.0 (7.1)	51.7 (7.1)
MVPA, meets 2008 guideline goals	44%	35%	39%	39%	30%
Sedentary time, upper quartile	19%	29%	21%	28%	36%
Hours of sleep, mean	7.9 (1.5)	7.9 (1.5)	7.8 (1.4)	7.8 (1.3)	7.7 (1.2)

*Numbers indicate percent or mean (standard deviation). SASH* Short Acculturation Scale for Hispanics (Marin [11], higher score indicates more non-Hispanic/Latino social relations), *AHEI* Alternative Healthy Eating Index (Chiuve [12], higher score indicates more healthful diet), *MVPA* moderate-to-vigorous physical activity, which was here defined according to whether individuals met 2008 US guidelines for healthy lifestyle (Ekelund, [13])

* English: “Did your family ever experience a period of time when they had trouble paying for their basic needs, such as food, housing, medical care, and utilities, when you were a child? / Spanish: ¿Su familia alguna vez tuvo dificultades para pagar sus necesidades básicas como comidas, vivienda, cuidados médicos, o servicios públicos, cuando usted era niño(a)?”

† English: “When you were growing up, did your home have the following basic utilities?... plumbing, septic tank. / Spanish: ¿Cuándo usted estaba creciendo, la casa donde vivía tenía los siguientes servicios públicos? Plomería, Drenaje/fosa séptica”

‡ English: “Of Hispanic/Latino and American food, do you usually eat...? Mainly or Mostly Hispanic/Latino foods” / Spanish: “De la comida hispana/latina y la comida americana, ¿por lo general come usted...? Principalmente comidas hispanas/latinas, or Mayormente comidas hispanas/latinas y algunas comidas americanas”

### Analysis of GMB composition and its correlates

Several markers of gut microbiome community structure were defined. We quantified alpha diversity using the Shannon index to describe the 16S rRNA gene V4 region bacterial and ITS1 fungal microbiome. We also derived the *Prevotella* to *Bacteroides* ratio from 16S data; these taxa frequently appear as important and dominant in other gut microbiome studies [14–16], hence the focus here. From analysis of the Bray-Curtis community ordination, we performed principal coordinate analysis (PcoA) using the 16S and ITS1 data. The first 16S principal coordinate (PCoA1) was strongly correlated with the *Prevotella* to *Bacteroides* ratio (Spearman’s *r* = − 0.89), while PCoA2 was correlated strongly with Shannon index (*r* = 0.77). Correlations with PCoA1 were − 0.89 and 0.94 for relative abundance of *Prevotella* and *Bacteroides*, respectively.

**Fig. 1:**
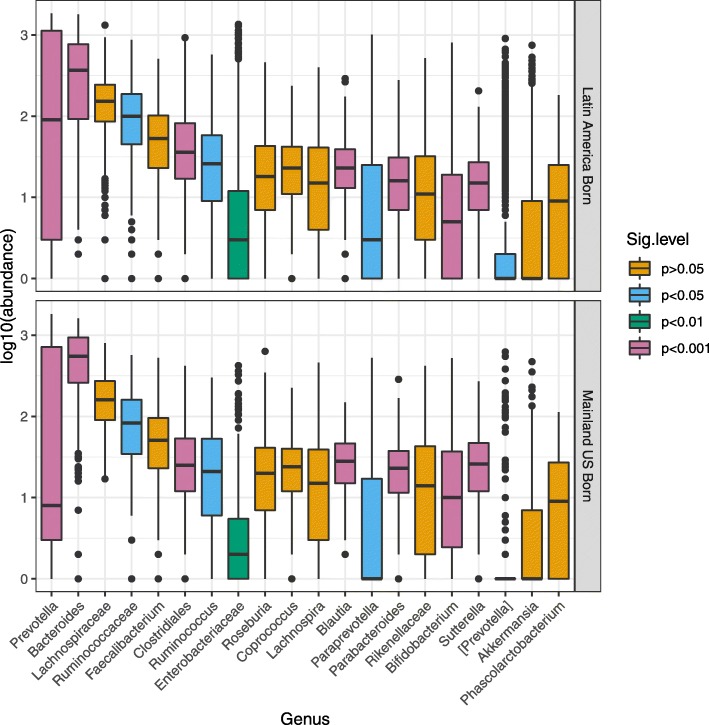
Relative frequency (median) and variability (interquartile range) of the 20 most abundant bacterial genera, among participants born in the mainland USA and those born in Latin America. Bar plots show the top 20 OTUs by order of abundance combined at the genus level using the phyloseq package. Boxes were colored based on the degree of statistical significance between US-born and foreign-born participants according to Wilcoxon signed-rank test to evaluate statistical significance between groups

**Fig. 2:**
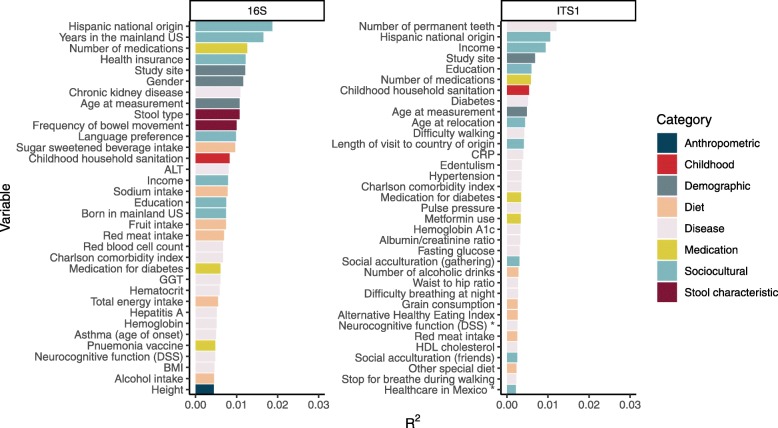
Correlates of gut bacterial (left) and fungal (right) microbiome in Hispanic/Latino residents of the USA, ranked by *R*^2^ values. Correlations were calculated using ordination based on pairwise Bray-Curtis distances. PERMANOVA analysis using the adonis function from the *vegan* package was used to assess statistical significance. The figure shows the top 35 variables, all with *P* values < 0.05, ranked by the estimated effect size. Variables were examined individually, rather than in a multivariable model containing all variables. Except where indicated by an asterisk, all variables met *q* value criteria < 0.05

Genus-level analysis of bacterial (16S) data from Hispanic adults showed that *Bacteroides* had the highest relative abundance both in those born in Latin America and those born the mainland USA (Fig. 1). In contrast, *Prevotella* had higher prevalence among Latin American-born individuals, as compared with the mainland US-born Hispanics. Among the most commonly occurring genera, *Prevotella* also had the highest variability as defined by the interquartile range in both US-born and Latin American-born groups of participants. Within *Prevotella*, we found that *P*. *copri* was the dominant species, comprising 88.7% of *Prevotella* albeit with a substantial count of unclassified species (Additional file 1: Table S1). US-born and Latin American-born individuals had similar abundance rankings of other common bacterial taxa (Fig. 2). In bivariate analyses, some taxa reached nominal statistical significance (*P* < 0.05) for differences in abundance by birthplace, including *Ruminococcaceae*, *Clostridiales*, *Bifidobacterium*, *Blautia*, *Enterobacteraceae*, and *Sutterella*.

Fungal (ITS1) GMB populations were dominated by *Aspergillus proliferans* and *Saccharomyces cerevisiae* in both mainland US-born and Latin American-born groups (Table 1). Relative abundance of several fungal taxa showed differences according to place of birth. Those born in the mainland USA had a mean relative abundance of *Cyberlindnera jadinii* of 5.8%, which was many folds higher than that among Latin American-born groups. *Candida sake*, *Candida tropicalis*, *Candida glabrata*, and *Rhodotorula mucilaginosa* were nearly absent in the US-born group but were fairly abundant in the range of 1 to 7% in Latin American-born populations.

Univariate analyses of 156 participant characteristics and health-related phenotypes, including dietary behaviors and disease-associated variables, were evaluated one-by-one by calculating beta diversity based on genus-level bacterial 16S and fungal ITS1 data. Multiple sociodemographic variables reflecting country of birth and relocation from Latin America to the mainland USA were identified in the top 35 variables (all *P* < 0.05) associated with Bray-Curtis distance in bacterial and fungal community-level analyses (Fig. 2). Nearly all of the variables associated with Bray-Curtis distance also met *q* value criteria of < 0.05, with a few exceptions for ITS1 analyses (Fig. 2).

### Relocation to the US mainland is associated with GMB composition

**Fig. 3:**
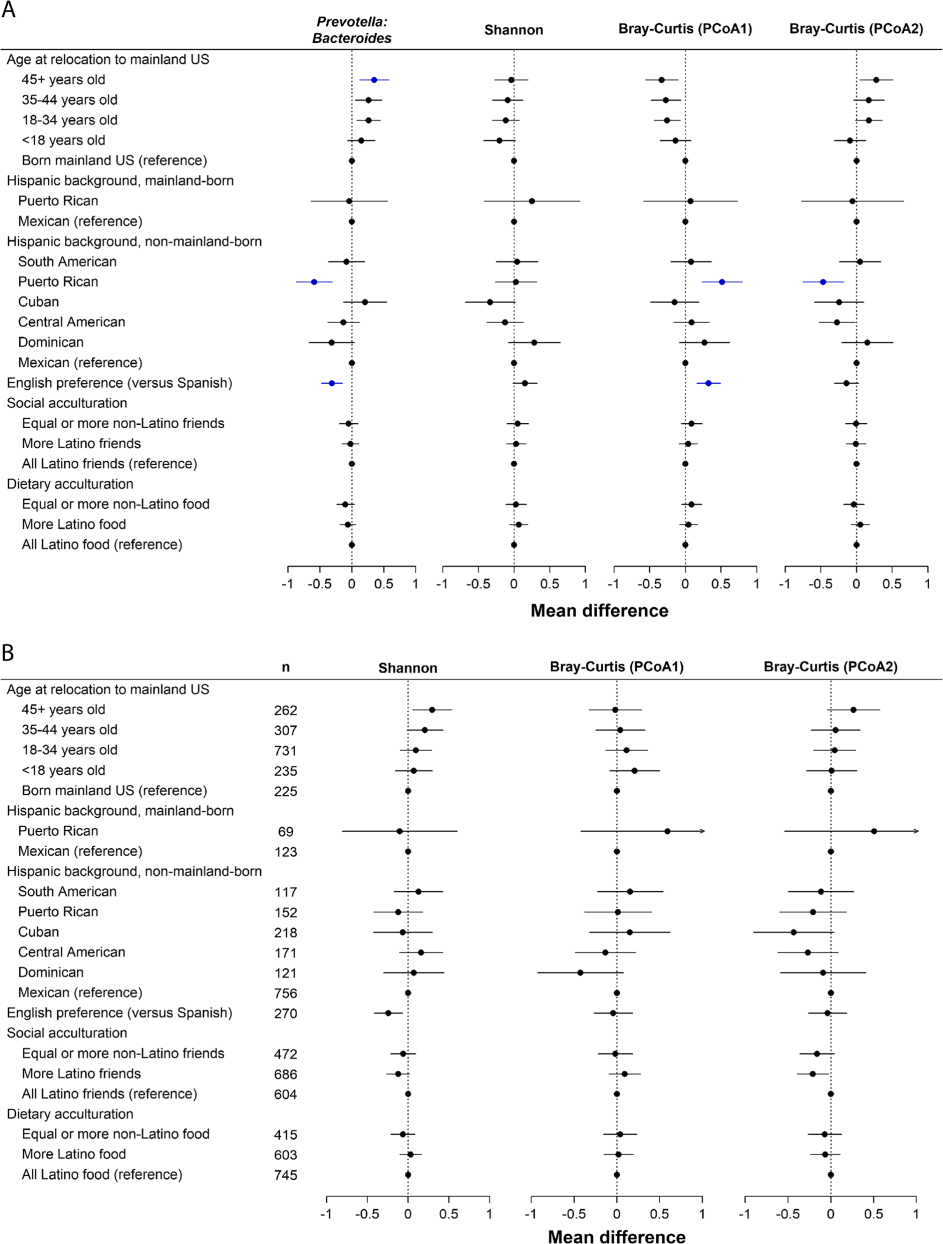
Association of birthplace and acculturation-related variables with bacterial 16S (**a**) and fungal ITS1 (**b**) gut microbiome features. **a** Bacterial microbiome associations. Shown are the results of models adjusted for age (except for the model for age at relocation), sex, field center, intake of vegetables without potatoes, intake of whole fruit, intake of whole grains, moderate-to-vigorous physical activity (continuous), BMI (six groups), diabetes (three groups), length of visit to home country (continuous), education level (four groups), income level (five groups), antibiotic in last 6 months (binary), and metformin use (binary). Plot shows linear regression beta estimates and 95% confidence intervals for mean standardized gut microbiome outcomes. Estimates for which *q* value was less than 0.05 are shown in blue. Groups with less than 15 individuals were excluded from comparison, specifically: among individuals born in the US mainland, group sizes were as follows: South American, 7; Cuban, 12; Central American, 7; Dominican, 5. Sample sizes (n) for panel **a** are the same as those displayed in panel **b**. **b** Fungal microbiome associations, analyzed in similar manner as described in A. No estimates in panel **b** had a *q* value less than 0.05. Arrows indicate that the upper confidence limit exceeded the range of the *X* axis. PCoA1 and PCoA2 denote first and second principal coordinate from principal coordinate analysis.

Systematic analysis was undertaken to discern the birthplace and migration-related factors that were independently associated with GMB (Fig. 3a). Multivariable adjustment was performed for gender, study center, intake of vegetables excluding potatoes, intake of whole fruit, intake of whole grains, moderate-to-vigorous physical activity (MVPA), body mass index (BMI), diabetes, visits returning to home country, education and income, and medications including use of antibiotics and metformin. *Prevotella* to *Bacteroides* ratio was lowest among those born in the US mainland (Fig. 3a). Among those born in Latin America, *Prevotella* to *Bacteroides* ratio increased monotonically with increasing relocation age. Analyses of bacterial (16S) Shannon index failed to find a clear “dose response” between timing of exposure to the USA and bacterial diversity. (Fig. 3a). Point estimates suggested that the Latin America-born group who relocated to the USA after 45 years of age had high bacterial alpha diversity, while in contrast, those who relocated from Latin America before 18 years of age had the lowest bacterial alpha diversity. Enigmatically, a high bacterial alpha diversity was also found in the US-born group. Confidence intervals for the groups were largely overlapping, and none of these findings for bacterial alpha diversity met *q* value criteria of < 0.05. Fungal (ITS1) Shannon index was lowest among individuals with early-life US exposure (i.e., born in the mainland USA or relocated prior to age 18 years), and highest among those who relocated to the mainland USA after age 45 (Fig. 3b).

**Fig. 4:**
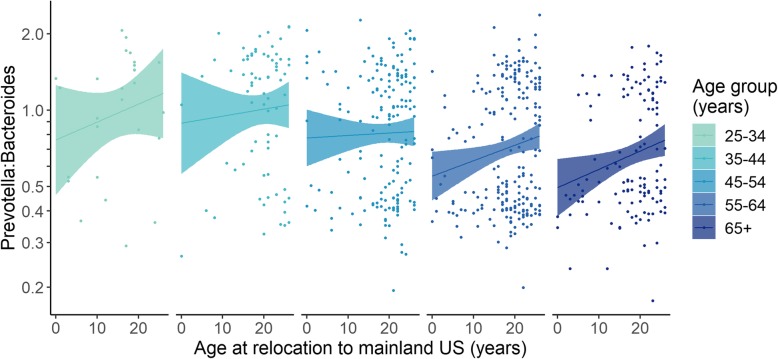
Linear regression analyses relating *Prevotella* to *Bacteroides* ratio with age at relocation to the mainland USA, among individuals who relocated to the USA before age 26. At the time they were studied, all cohort members were 25 years or older. Thus, by limiting to those who relocated to the mainland USA between birth and age 25, we could make a comparable analysis of age at relocation within groups defined by attained age. For example, only the oldest age groups of 55–64 years old (blue) or 65+ years old (magenta) could have contained an individual who had migrated at age 50 years old. However, any of the groups defined by attained age could have contained an individual who had migrated during childhood. As expected, after exclusion of those who relocated after age 26, there was no correlation between age at relocation and current age (Additional file 1: Figure S4). Within each of the groups defined by attained age, we observed a trend of increasing *Prevotella* to *Bacteroides* ratio with older age at relocation to the mainland USA

Next, we addressed the issue of age confounding in analyses of GMB composition versus relocation age. Before conducting analyses within groups stratified by current age, we excluded individuals who had relocated to the USA after age 26 because this group was strongly biased towards having older current ages. The current age and relocation age were uncorrelated after this exclusion was applied (Beta = 0.017, 95% CI − 0.029, 0.063) (Additional file 1: Figure S3). We then examined the association of relocation age and *Prevotella* to *Bacteroides* ratio within each of five groups that were defined based on age at the time of the GMB study (25–34 years, 35–44 years, 45–54 years, 55–64 years, and 65 years and older). The association between childhood relocation to the USA and lower *Prevotella* to *Bacteroides* ratio was seen across the full range of attained age, up to and including the oldest group aged 65 years and older (Fig. 4). We thus were able to control for the potential confounding influence of current age, showing that the association of relocation age and *Prevotella* to *Bacteroides* ratio was independent of current age and indicating that the association between relocation age and *Prevotella* to *Bacteroides* ratio was durable across the life course.

We found little evidence that the geographic place of origin within Latin America had associations with summary measures of GMB composition (Fig. 3a and Fig. 3b). We conducted two additional analyses to discern whether the varied national backgrounds of our participants influenced our results. The association between birthplace and relocation age with *Prevotella* to *Bacteroides* ratio and GMB diversity was similar after serial exclusion of each Latino background group, indicating that a single group was not disproportionately influencing the overall result (data not shown). A subgroup analysis that was limited to the Mexican/Mexican American individuals was also conducted (Additional file 1: Figure S4), and it generally supported the overall conclusions derived from analyses shown in Fig. 3 a and b for the overall population.

**Fig. 5:**
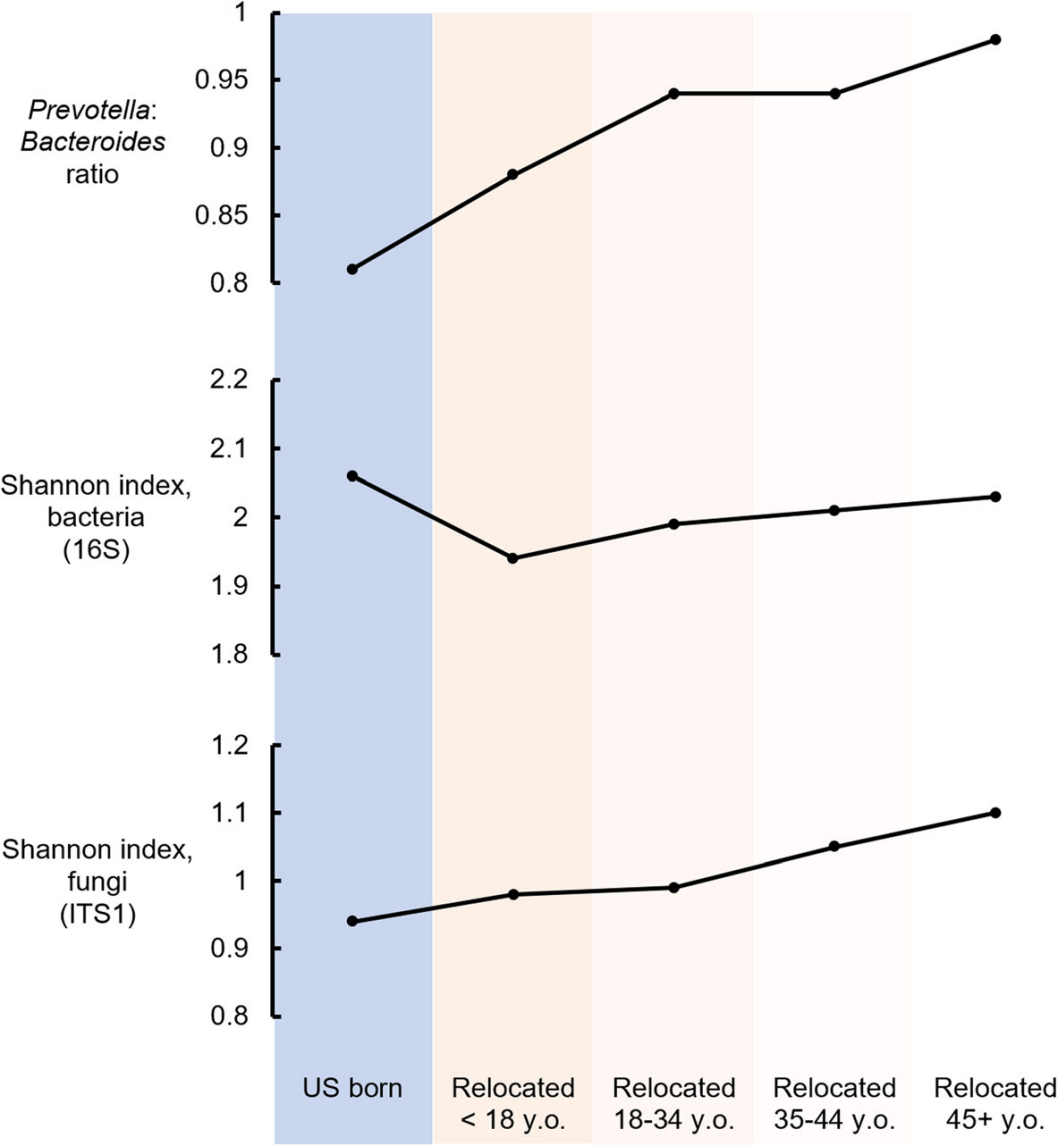
Summary of findings relating to acculturation and GMB among US Latinos. The graphs summarize the results relating birthplace and migration history with summary measures of GMB including *Prevotella* to *Bacteroides* ratio, bacterial and fungal diversity. Older age at arrival to the mainland USA, consistent with the least exposure to the USA and the lowest acculturation to the US lifestyle, was associated with the highest *Prevotella* to *Bacteroides* ratio. This profile also went along with high fungal alpha diversity. Results for the bacterial GMB community were more complex. High bacterial diversity was found among the US born, and also among the group who relocated to the mainland USA from Latin America in older adulthood. The lowest bacterial alpha diversity was observed among those relocating from Latin America to the mainland USA during early life. Values were derived from fitted multivariate linear regression models as predicted mean outcomes in each category of birthplace and age at relocation, holding model covariates constant at either their mean value (for continuous variables, length of visit to home country/territory, intake of whole fruits, whole vegetables and whole grains, moderate-to-vigorous physical activity) or at the value of the most frequent category variable response (sex = female, field center = Chicago, body mass index = overweight, glycemic status = abnormal, metformin use = no, education = greater than high school level, annual income = $20,000 to $40,000/year, and antibiotic use in the last 6 months = no)

Figure 5 summarizes the results described above relating GMB with exposure to the USA, as defined by birthplace and age at arrival to the mainland USA. *Prevotella* to *Bacteroides* ratio and fungal alpha diversity were lowest among individuals with early-life exposure to the USA. These measures increased in linear fashion across groups with later-life arrival in the USA. In contrast, bacterial alpha diversity was highest among the US-born and those who migrated from Latin America to the mainland USA after age 45 years old, whereas this GMB characteristic was lowest in childhood arrivals from Latin America to the USA.

### Association between acculturation factors and GMB

Next we sought to understand the relationship of GMB with acculturation, or adaptation of features of the US environment, which varied across birthplace and relocation age groups (Table 2) [17–19]. English language preference was associated with lower *Prevotella* to *Bacteroides* ratio and lower fungal Shannon diversity (Fig. 3a). However, English language preference was associated with higher Shannon bacterial diversity (versus Spanish language, beta _English preference_ = 0.09 higher 16S Shannon index, 95% confidence interval, 0.01, 0.16); this contradicts the hypothesis that increasing exposure to the USA leads to depletion of the bacterial microbiome. Those consuming primarily “American” foods rather than “Hispanic” foods (dietary acculturation) had significantly lower *Prevotella* to *Bacteroides* ratio, although this dietary acculturation variable was not associated with alpha diversity. Social acculturation, which captures whether social interactions mainly involved other Latino or non-Latinos, had no association with *Prevotella* to *Bacteroides* ratio or alpha diversity.

**Table 2:** Relative abundance of fungal species (ITS1 classification) comparing HCHS/SOL participants by region of birth

	Country of birth						
	Cuba	Dominican Republic	Puerto Rico	Mexico	South America	Central America	Mainland USA
*Aspergillus proliferans*, abundance	0.218	0.189	0.228	0.188	0.163	0.202	0.126
Ratio versus mainland USA	1.7	1.5	1.8	1.5	1.3	1.6	
*Saccharomyces cerevisiae*, abundance	0.318	0.234	0.322	0.271	0.294	0.277	0.413
Ratio versus mainland USA	0.8	0.6	0.8	0.7	0.7	0.7	
*Candida albicans*, abundance	0.069	0.202	0.118	0.100	0.100	0.115	0.113
Ratio versus mainland USA	0.6	1.8	1.0	0.9	0.9	1.0	
Candida sake, abundance	0.059	0.058	0.045	0.053	0.071	0.052	~ 0.001
Ratio versus mainland USA	70.2	69.4	53.7	63.3	84.3	61.8	
*Debaryomyces hansenii*, abundance	0.034	0.027	0.014	0.039	0.023	0.029	0.048
Ratio versus mainland USA	0.7	0.6	0.3	0.8	0.5	0.6	
*Candida tropicalis*, abundance	0.031	0.011	0.010	0.013	0.018	0.034	0.006
Ratio versus mainland USA	5.7	1.9	1.8	2.4	3.4	6.2	
*Wallemia muriae*, abundance	0.049	0.043	0.052	0.044	0.038	0.042	0.066
Ratio versus mainland USA	0.7	0.7	0.8	0.7	0.6	0.6	
*Candida glabrata*, abundance	0.027	0.012	0.036	0.020	0.016	0.013	< 0.001
Ratio versus mainland USA	78.4	35.0	107.0	59.6	46.5	39.1	
*Cyberlindnera jadinii*, abundance	0.006	0.002	0.018	0.026	0.013	0.020	0.058
Ratio versus mainland USA	0.1	0.0	0.3	0.4	0.2	0.3	
*Rhodotorula mucilaginosa*, abundance	0.011	0.008	0.014	0.023	0.020	0.033	< 0.0001
Ratio versus mainland USA	1055.7	795.9	1378.5	2314.3	2047.8	3290.0	

### Association between diet and GMB

We next examined variation in dietary habits across Hispanic groups, which was previously shown in our cohort [20]. Latin America-born individuals, especially those who relocated to the US mainland during later adulthood, had the most favorable eating habits, evidenced by a higher Alternative Healthy Eating Index - 2010 (AHEI) score, a summary measure of diet quality (Table 2), lower consumption of fats and sodium, and higher consumption of fiber (Table 3). Fiber was further analyzed by food sources (Additional file 1: Table S2 displays definition of food group-derived variables.) We found no significant variation in bean/legume intake according to US-born or relocation age groups. Instead, fruit and whole grains were the sources of fiber that appeared to differ across the population, favoring the adult age immigrants to the USA who had higher intakes of these foods. More favorable AHEI diet score was associated with higher *Prevotella* to *Bacteroides* ratio (beta _1 AHEI unit_ = 0.0063, 95% confidence interval 0.0027, 0.0100, *P* value = 0.0062) (Table 4). AHEI was not associated with alpha diversity for 16S (beta = −.0004, 95% confidence interval − .0048, 0.0040, *P* value = 0.34) or for ITS1 (beta = 0.006, 95% confidence interval 0.0010, 0.0099, *P* value = 0.40). Four specific foods that were associated with higher *Prevotella* to *Bacteroides* ratio were higher whole grains, higher vegetables, lower red meat, and lower trans fats (Table 5). Higher grain intake was associated with lower bacterial (16S) alpha diversity, while higher vegetable intake was associated with higher fungal (ITS1) alpha diversity (Table 5).

**Table 3:** Diet among HCHS/SOL participants, classified according to place of birth and age at relocation from Latin America to the mainland USA

	Mainland US born, Mean (SE)	Relocated 0–17 years old, Mean (SE)	Relocated 18–34 years old, Mean (SE)	Relocated 35–44 years old, Mean (SE)	Relocated 45+ years old, Mean (SE)
*N*	225	235	730	306	261
Overall diet					
AHEI score, mean	47.4 (0.4)	49.2 (0.4)†	51.4 (0.2) †	51.4 (0.4) †	51.4 (0.4) †
Dietary acculturation scale*	2.6 (0.06)	2.2 (0.05) †	1.7 (0.03) †	1.5 (0.05) †	1.5 (0.05) †
Nutrient intake					
Energy, mean (kcal)	1962 (23)	1918 (22)	1888 (13) †	1899 (19)	1903 (22)
Carbohydrates, mean (g)	234 (1.6)	241 (1.5) †	242 (0.9) †	245 (1.3) †	245 (1.5) †
Carbohydrates, mean (% calories)	50.9 (0.2)	51.8 (0.2) †	52.2 (0.1) †	52.7 (0.2) †	52.6 (0.2) †
Protein, mean (g)	77.1 (0.5)	77.2 (0.5)	78.2 (0.3)	77.7 (0.4)	77.9 (0.5)
Protein, mean (% calories)	16.9 (0.1)	16.9 (0.1)	17.1 (0.1)	17.0 (0.1)	17.1 (0.1)
Fat, mean (g)	67.5 (0.5)	65.6 (0.4) †	64.5 (0.3) †	63.2 (0.4) †	63.0 (0.4) †
Fat, mean (% calories)	30.9 (0.2)	30.2 (0.2) †	29.8 (0.1) †	29.3 (0.1) †	29.2 (0.2) †
Saturated fat, mean (% calories)	10.2 (0.1)	9.9 (0.1) †	9.7 (0.0) †	9.6 (0.1) †	9.5 (0.1) †
Saturated fat, mean (g)	22.1 (0.2)	21.3 (0.2) †	20.8 (0.1) †	20.5 (0.2) †	20.4 (0.2) †
Transfat, mean (g)	2.9 (0.04)	2.6 (0.04) †	2.4 (0.02) †	2.4 (0.04) †	2.3 (0.04) †
Dietary fiber, mean (g)	16.5 (0.3)	17.9 (0.2) †	19.1 (0.1) †	19.3 (0.2) †	19.1 (0.2) †
Sodium, mean (g)	3332 (40)	3179 (37) †	3141 (22) †	3159 (33) †	3152 (37) †
Intake of specific foods					
Vegetable excluding potato, mean (servings/day)	1.8 (0.2)	1.9 (0.1)	2.0 (0.1)	2.0 (0.1)	2.0 (0.1)
Fruit excluding juice, mean (servings/day)	0.7 (0.1)	1.0 (0.1)	1.2 (0.1) †	1.3 (0.1) †	1.3 (0.1) †
Whole grain, mean (servings/day)	1.0 (0.2)	1.5 (0.2) †	1.9 (0.1) †	1.8 (0.1) †	1.9 (0.2) †
Refined grain, mean (servings/day)	4.9 (0.2)	4.7 (0.2)	4.5 (0.1)	4.9 (0.2)	4.6 (0.2)
Meat, mean (servings/day)	6.4 (0.3)	5.9 (0.2)	5.7 (0.1)	5.4 (0.2) †	5.3 (0.2) †
Red meat, mean (servings/day)	1.9 (0.2)	1.8 (0.2)	1.8 (0.1)	1.8 (0.2)	1.8 (0.2)
Processed meat, mean (servings/day)	0.9 (0.1)	0.9 (0.1)	0.7 (0.1)	0.7 (0.1)	0.9 (0.1)
Nuts and legumes, mean (servings/day)	1.0 (0.1)	0.7 (0.1)	0.9 (0.1)	1.0 (0.1)	1.0 (0.1)
Sugar sweetened beverages, mean (servings/day)	1.2 (0.1)	1.3 (0.1)	1.2 (0.1)	1.2 (0.1)	1.3 (0.1)
Milk/dairy, mean (servings/day)	1.5 (0.1)	1.6 (0.1)	1.6 (0.1)	1.6 (0.1)	1.5 (0.1)
Alcohol, mean (servings/day)	0.35 (0.03)	0.28 (0.03)	0.26 (0.02)	0.30 (0.03)	0.32 (0.03)

*AHEI* Alternative Healthy Eating Index (Chiuve [12])

*Dietary acculturation scale 1 = Mainly Latino food, 2 = Mostly Latino food and some American food, 3 = Equal, 4 = Mostly American food, 5 = Mainly American food

Nutrient intake was predicted from an amount model (i.e., a one-part non-linear mixed model) specified by the NCI method (Tooze, [21]), using single-component SAS macros developed at NCI http://riskfactor.cancer.gov/diet/usualintakes/macros.html. This method estimates the within- and between-person components and corrects for the high intra-individual variation intrinsic to 24-h recalls given that individuals do not eat the same foods every day. We excluded recalls with daily energy intake below the recall-gender-specific 1st percentile or above the 99th percentile, and recalls that were unreliable according to the interviewer

Column comparisons are adjusted for age, sex, and field center. Additional adjustment has been made for predicted daily energy intake, for analyses of nutrients and specific foods

† *P* < 0.05 comparing to US born (*P* adjusted for pairwise comparison)

**Table 4:** Association of socioeconomic variables and diet quality with features of the gut microbiome

	16S Shannon index	P:B ratio	ITS Shannon index
	Beta (95% CI)	Beta (95% CI)	Beta (95% CI)
Household income/year ($)			
< 10,000	Reference	Reference	Reference
10,000–20,000	0.03 (− 0.06, 0.13)	0.04 (− 0.04, 0.12)	0.10 (0.00, 0.20)
20,000–40,000	0.02 (− 0.08, 0.11)	0.02 (− 0.06, 0.10)	0.10 (0.01, 0.20)
> 40,000	0.08 (− 0.02, 0.18)	− 0.11 (− 0.19, − 0.03)	0.10 (− 0.01, 0.20)
*P* value	0.16	0.0005	0.22
Educational attainment			
< 9th grade	Reference	Reference	Reference
Some high school	0.01 (− 0.09, 0.11)	− 0.09 (− 0.18, − 0.01)	− 0.02 (− 0.12, 0.08)
High school diploma	− 0.03 (− 0.11, 0.05)	− 0.08 (− 0.15, − 0.01)	0.01 (− 0.08, 0.09)
Beyond high school	0.02 (− 0.05, 0.09)	− 0.15 (− 0.21, − 0.09)	0.05 (− 0.02, 0.12)
*P* value	0.62	< 0.0001	0.15
Childhood economic hardship	− 0.02 (− 0.10, 0.07)	0.05 (− 0.02, 0.12)	0.01 (− 0.07, 0.10)
*P* value	0.72	0.14	0.77
Childhood sanitation (had basic facilities such as plumbing and sewer tank)	− 0.03 (− 0.14, 0.08)	− 0.14 (− 0.23, − 0.05)	0.08 (− 0.03, 0.19)
*P* value	0.60	0.003	0.17
Alternative Healthy Eating Index*	−.0004 (−.0048, 0.0040)	0.0063 (0.0027, 0.0100)	0.0055 (0.0010, 0.0099)
*P* value	0.35	0.006	0.40

Linear regression models are adjusted for age, sex, and field center

* For Alternative Healthy Eating Index (Chiuve, [81]), a higher score means a healthier diet

**Table 5:** Association of foods and nutrients with gut microbiome composition

	16S Shannon index	*Prevotella* to *Bacteroides* ratio	ITS1 Shannon index
	Beta (95% CI)	Beta (95% CI)	Beta (95% CI)
Whole grains	*− .0280 (− .0553, − .0006)*	*0.0417 (0.0187, 0.0647)*	0.0219 (− .0068, 0.0505)
Grains	*− .0112 (− .0207, − .0016)*	0.0007 (− .0073, 0.0088)	0.0016 (− .0083, 0.0116)
Legumes	− .0467 (− .1392, 0.0457)	0.0723 (− .0056, 0.1501)	0.0382 (− .0579, 0.1344)
Whole fruit	0.0237 (− .0172, 0.0646)	0.0078 (− .0267, 0.0422)	0.0366 (− .0061, 0.0794)
Vegetables (no potatoes)	0.0100 (− .0377, 0.0576)	*0.0638 (0.0237, 0.1039)*	*0.0527 (0.0032, 0.1023)*
Sugar sweetened beverages	− .0148 (− .0502, 0.0206)	0.0151 (− .0147, 0.0449)	−.0026 (− .0398, 0.0347)
Red and processed meat	− .0042 (− .1168, 0.1084)	*− .1310 (− .2257, − .0364* **)**	−.0790 (− .1956, 0.0376)
Fiber	− .0016 (− .0076, 0.0044)	0.0044 (− .0007, 0.0094)	0.0060 (− .0003, 0.0122)
Transfat	0.0654 (− .0698, 0.2006)	*− .2413 (− .3546, − .1279)*	−.0484 (− .1897, 0.0928)
PUFA	0.0071 (− .0327, 0.0469)	− .0150 (− .0486, 0.0185)	−.0001 (− .0417, 0.0415)
Alcohol	0.0091 (− .0493, 0.0676)	0.0014 (− .0478, 0.0507)	0.0267 (− .0343, 0.0876)

Models adjusted for age, sex, and field center. *Italics indicate* *P*<0.05. *PUFA* polyunsaturated fatty acid

Definitions of composite food group variables are described in Additional file 1: Table S2

### Physical activity habits

Using data from 7-day accelerometry, we observed that late-life migrants to the USA had the worst physical activity habits (Table 2). However, there was no evidence that physical activity habits were related to measures of GMB composition including diversity or *Prevotella* to *Bacteroides* ratio (data not shown).

### Association between socioeconomic variables and GMB

As compared with those who relocated to the US mainland in adulthood, both mainland US-born individuals and those arriving during childhood (age 0 to 17 years) had greater attained height, which is a marker of early-life socioeconomic advantage, and larger current household income (Table 2). Lower ratio of *Prevotella* to *Bacteroides* was associated with annual household income above $40,000 and higher educational attainment (Table 4). Conversely, higher *Prevotella* to *Bacteroides* ratio was found among those who lacked plumbing facilities during childhood.

### Obesity

The US Latino population has a significant burden of obesity [18]. Therefore, we next examined the potential role of GMB in obesity as has been shown in other populations [4, 22].

Relatively few individuals (*N* = 293) had body mass index in the healthy range of 18.5 to 25 kg/m^2^, while a similar number (approximately 17%) of the cohort had class II obesity (*N* = 188, BMI 35 kg/m^2^ to 40 kg/m^2^) or class III obesity (*N* = 106, BMI above 40 kg/m^2^). Geographic region of birth and timing of relocation to the mainland USA were associated with obesity, and especially class II–III obesity (Additional file 1: Figure S5, Additional file 1: Table S3, and reference [18]).

**Fig. 6:**
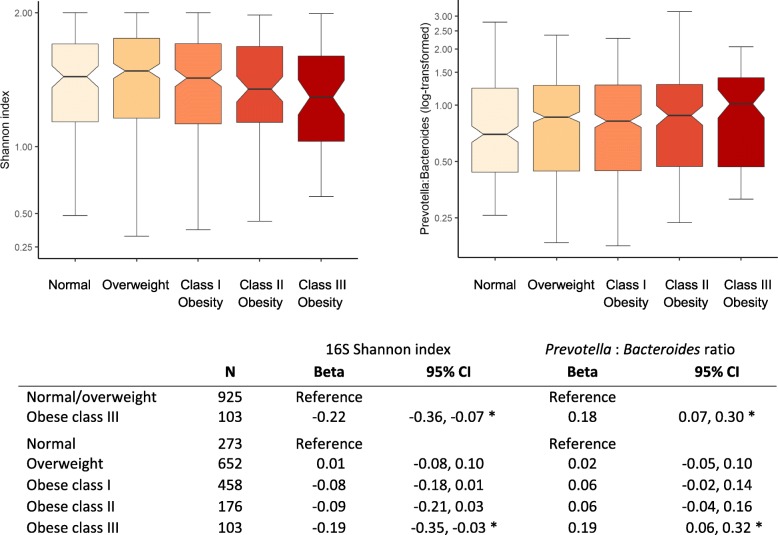
Association of gut microbiome features with obesity defined by body mass index. Beta and 95% confidence intervals were derived from linear regression models relating body mass index categories with 16S Shannon index (left) and *Prevotella* to *Bacteroides* ratio (right), after adjustment for age, sex, field center, intake of vegetables without potatoes, intake of whole fruit, intake of whole grains, moderate-to-vigorous physical activity (continuous), diabetes (three groups), length of visit to home country (continuous), education level (four groups), income level (five groups), antibiotic in last 6 months (binary), and metformin use (binary). Asterisks indicate *P* < 0.05. Body mass index (BMI) defined as normal, 18.5 to 25 kg/m^2^; overweight, 25 to 30 kg/m^2^; class I obesity, 30 to 35 kg/m^2^; class II obesity, 35 to 40 kg/m^2^; and class III obesity, BMI above 40 kg/m^2^. CI, confidence interval

The association between GMB and obesity is shown in Fig. 6. Higher levels of obesity were associated with lower bacterial alpha diversity (Shannon index) and higher *Prevotella* to *Bacteroides* ratio, after adjustment for confounders. Measures of ITS1 composition had no evidence of association with obesity (data not shown).

### Identification of bacterial and fungal taxa associated with birthplace, relocation, and obesity

We next screened 74 bacterial genera with relative abundance > 0.01% to identify taxa associated with body mass index and relocation age. Of the 74 bacterial genera, after FDR correction at *P* trend < 0.05, 20 genera were significantly associated with obesity (Additional file 1: Table S4), and 29 genera were significantly associated with birthplace and relocation age (Additional file 1: Table S5). Cross-classification of these two sets of results identified 10 bacterial genera that showed significant associations with both birthplace/relocation age and obesity (*Oscillospira*, *Acidaminococcus*, *Megasphaera*, *Anaerotruncus*, *Unclassified.Ruminococcaceae*, *Unclassified.Coriobacteriaceae*, *Unclassified.Clostridiales*, *Unclassified.Christensenellaceae*, *Unclassified.YS2 (Cyanobacteria)*, and *Unclassified.Victivallaceae,* Table 6 and Additional file 1: Figure S6). Of these 10 bacterial genera, 2 were positively associated both with obesity and with early-life exposure to the mainland USA, and 6 were negatively associated both with obesity and early-life exposure to the mainland USA. Others, including *Oscillospira* and *Anaerotruncus*, were similar to *Prevotella* to *Bacteroides* ratio in that they displayed the paradoxical pattern of being associated both with normal weight and with early-life US exposure.

**Table 6:** Regression analyses relating genera with obesity, birthplace, and age at relocation to the mainland USA. After individually examining the associations of 74 genera with relative abundance > 0.01% with obesity and with birthplace/age at relocation to the mainland USA, the ten genera displayed in this table were found to be overlapping between these two analyses. Regression models for obesity adjusted for age, sex, and center, and regression models for birthplace and age at relocation adjusted for sex and center

	Association with obesity			Association with birthplace and age at relocation to USA			Direction of association		
		*P* value	*P* value		*P* value	*P* value			
	Beta	Unadj	FDR adj	Beta	Unadj	FDR adj	Obesity	Early-life exposure to USA	Consistent?
*Oscillospira*	− 0.139	< 0.001	< 0.001	− 0.071	0.003	0.0111	Negative	Positive	No
*Unclassified.Clostridiales*	− 0.207	< 0.001	< 0.001	0.101	< 0.001	< 0.001	Negative	Negative	Yes
*Unclassified.Ruminococcaceae*	− 0.138	< 0.001	< 0.001	0.067	0.005	0.016818	Negative	Negative	Yes
*Unclassified.Christensenellaceae*	− 0.426	< 0.001	< 0.001	0.158	0.003	0.0111	Negative	Negative	Yes
*Unclassified.Coriobacteriaceae*	− 0.235	< 0.001	< 0.001	0.137	0.001	0.0056923	Negative	Negative	Yes
*Megasphaera*	0.277	< 0.001	< 0.001	− 0.167	0.003	0.0111	Positive	Positive	Yes
*Acidaminococcus*	0.318	< 0.001	< 0.001	− 0.3	< 0.001	< 0.001	Positive	Positive	Yes
*Anaerotruncus*	− 0.18	< 0.001	< 0.001	− 0.083	0.011	0.03256	Negative	Positive	No
*Unclassified.Victivallaceae*	− 0.144	0.009	0.037	0.118	0.004	0.014095	Negative	Negative	Yes
*Unclassified.YS2*	− 0.217	0.004	0.0185	0.205	< 0.001	< 0.001	Negative	Negative	Yes

Fungal ITS1 classification yielded 16 class-level, 49 order-level, 109 family-level, 192 genus-level, and 396 species-level taxa (Additional file 2: Table S6). Analysis of fungal taxa (Additional file 1: Table S7) revealed a few differences comparing those born in the mainland USA versus those born in Latin America (|LDA score| > 10^4^) (*Aspergillus*, *Cyberlindnera*, Tremellomycetes). Furthermore, in analysis of relocation age, among the 23 predominant fungal genera with relative abundance > 0.01% and present in more than 5% of individuals, *Candida* achieved an FDR-adjusted *P* value of 0.046 (Additional file 1: Table S8), while four others met nominal but not FDR-adjusted *P* value < 0.05 (*Cyberlindnera*, *Aspergillus*, *Mrakia*, *Saccharomyces*). We did not find any fungal correlates of obesity, with only *Debaryomyces* achieving a nominal *P* value < 0.05 (*P* value = 0.299 after FDR correction) (Additional file 1: Table S9).

## Discussion

The study of the human microbiome provides a new approach to understand health consequences of the environment across different geographic regions. Prior data suggest that gut microbiomes of Hispanic/Latino adults appear as a distinct cluster when analyzed alongside a collection of USA and worldwide populations [7, 23]. The results presented here describe characteristics of GMB variation and their determinants within the US Hispanic population. GMB heterogeneity among the US Latino study population was significantly accounted for by differences between the “first-generation” (Latin America-born) and “second-generation” (mainland US-born) groups. Each group had its own distinct microbiome pattern which was dependent both upon place of birth and timing of geographic relocation to the mainland USA (e.g., “relocation age”). People who relocated to the mainland USA from Latin America, particularly those who did so relatively late in life, were characterized by a relatively high ratio of *Prevotella* to *Bacteroides*. This accounts for the fact that migration- and acculturation-related variables were among the leading explanatory variables in Bray-Curtis distance clustering analyses of 16S sequence data when ranked by explained variation (Fig. 2, *R*^2^). There was also evidence for increased GMB diversity of both bacterial and fungal components in arrivals from Latin America, particularly among those who arrived in the USA during middle to late adulthood as opposed to early life. Our data are consistent with the prevailing tendency for people in lower-income countries to have different gut microbial characteristics [24] including a *Prevotella*-dominant microbiome [4], when compared with the US population. In contrast to the Latin America-born, the US-born Latino population had low *Prevotella* to *Bacteroides* ratio and low fungal alpha diversity.

Among Hispanic populations, dietary patterns (fiber, sugary sweets, animal products, etc.) and medical history (e.g., diabetes, number of medications, Charlson comorbidity index) ranked high in terms of the variance explained according to community-wide comparisons, consistent with other cohorts [25]. A novel contribution of our study was our observation that the strength of sociodemographic, region of birth, and migration-related influences rivaled that of known contributors to GMB diversity. The findings are supportive of a strong and lasting influence of early-life environment on the gut microbiome. Our cohort of largely immigrant US Latinos captured the “1.5 generation,” a subset of the first generation which refers to those who relocated to the USA during childhood and adolescence. Individuals in this group have lived their adult life in the US environment, but during childhood development, their gut microbiomes would have been established under the influence of the Latin American environment and lifestyle. The “1.5 generation” had levels of *Prevotella* to *Bacteroides* ratio that were intermediate between the “first” and “second” generations. Particularly interesting was that relocation age effects were seen regardless of the current age of participants. Thus, the tendency for childhood arrivals with longer time living in the USA to have lower *Prevotella* to *Bacteroides* ratio as compared with adult arrivals was a consistent phenomenon that did not dissipate across the life course. This finding suggests a critical time window for establishment of the adult microbiome, in line with the observation that age at separation determined GMB concordance between twins in the UK Twins cohort [26]. We also showed that Hispanic adult US residents raised in Latin America had diet patterns that differed from that among the US-born. These differences in prevailing diet patterns were discernable even after immigrants had lived in the USA a long time, and they appeared to contribute to the makeup of the GMB. However, diet did not explain the GMB differences by birthplace and migration. The dual dependencies of both GMB and diet on the historical age at migration provide an interesting avenue of research to understand the long-term health of Hispanic populations of the USA.

In contrast to results for *Prevotella* to *Bacteroides* ratio, the association of GMB bacterial diversity with birthplace and geographic region was less clear. We found a relatively weak overall association between exposure to the USA and bacterial diversity. As compared both with those who relocated as adults, and those who were born in the mainland USA, those who relocated to the USA during childhood tended to have lower bacterial diversity. Moreover, those preferring to use the English language over the Spanish language had significantly higher 16S Shannon index, which was at odds with the a priori expectation that higher acculturation to the US environment would be associated with reduced bacterial alpha diversity. This seems to provide a more nuanced picture when compared with findings among other communities [24] which have observed loss of GMB diversity after migration from a low-to-moderate income setting to the USA. It should be noted that in some studies these immigrant generation differences in bacterial diversity have been relatively modest [4] and most studies have not analyzed data separately from the “generation 1.5” childhood-arrival population.

We confirmed the expected association of low bacterial (16S) diversity with obesity [4]. We also used classification of subjects according to *Prevotella* to *Bacteroides* ratio because it is a frequently used metric to define the microbiome, although it only captures one feature of microbiome space [16]. While decreasing *Prevotella* relative to *Bacteroides* was associated with exposure to the USA and “US style” (versus “Latino”) foods, enigmatically *Prevotella* to *Bacteroides* ratio tended to be higher rather than lower among obese individuals. Therefore, our results were not consistent with the hypothesis that “replacement” of *Prevotella* with *Bacteroides* among immigrants relocating to high-income nations is associated with increased risk of obesity. On the contrary, our data suggested that normal weight Latino adults had low prevalence of *Prevotella* relative to *Bacteroides*. While resolving specific species and strains could not be done from our 16S data, it seems clear that this will be an important next step for assessing health effects of the GMB in Hispanics. For example, *Prevotella copri* is a common species that has been associated with increased risks of various diseases including diabetes [27]. Both *Prevotella* [28] and *Bacteroides* [29] are highly diverse and with strain-specific gene functions that differ between Western and non-Western populations. As compared with the *Prevotella*-dominant GMB typical of the Latin American region, Latinos highly adapted to the USA who have a *Bacteroides*-dominant GMB may have different responses to dietary components and exposure to disease-related mechanisms such as short-chain fatty acid production and degradation of the GI mucus barrier [5, 6]. To resolve apparent differences between studies, an intriguing hypothesis that trans-cohort collaborations might be able to address states that disease-associated microbiota patterns may be different in different geographic regions [10].

Having observed a significant influence of dietary fiber on *Prevotella* to *Bacteroides* ratio, we considered whether types of carbohydrates, legumes, and starches consumed differed across subgroups of the Hispanic population. Fruit and whole grain consumption were variable in the population, favoring the older adult age immigrants to the USA who had higher intakes of these foods. Bean and legume consumption was high by US standards [30]. However, this food had similar consumption across the population, and based on our adjusted analyses, we consider this diet component unlikely to contribute to the observed GMB differences.

Additional analyses identified that several genera had the signature of a bacterial group that was related in the same direction both to obesity and to early-life US exposure. For instance, *Acidaminococcus* (anaerobic, Gram-negative, acetate- and butyrate-producer [31]) was more abundant both with high BMI and with mainland US birth. *Acidaminococcus* has been associated with metabolic disease risks in prior worldwide studies. Abundance of these bacteria may be reduced in type 1 diabetes (China [32], Mexico [33]) and increased in children with stunting (Malawi, Bangladesh) [34]. Consistent with our results, *Acidaminococcus* has been found to be increased in higher BMI adults (Bangladesh [35], USA [36]) and in adults with high combined cardiovascular risk factors (China) [37]. We also confirmed that those with unfavorable body weight had reduced abundance of *Oscillospira* [22], which has been also shown as a microbiome feature that correlates with fatty liver disease which is of particularly high prevalence among Latinos [38]. Paradoxically, although adiposity and US exposure are strongly associated with one another, *Oscillospira* as well as *Anaerotruncus* (another bacteria known to be negatively related with obesity) had lower abundance in the obese but higher abundance in the US-born. This discordant pattern between these two epidemiologically linked participant characteristics was therefore seen for *Prevotella*, *Anaerotruncus*, and *Oscillospira*, which we consider an interesting finding albeit of uncertain interpretation.

We found an association of reduced mycobiome diversity with early-life exposure to the USA. Components of the mycobiome have been implicated in chronic disease risk, but this is an understudied area [39]. The lead explanatory variable for fungal beta diversity (Bray-Curtis distance) was poor oral health (missing teeth), and oral health overall is poor in the Latino population, as shown for the groups enrolled in HCHS/SOL [40]. Fungal diversity also varied by income and neighborhood of residence (census block), which may be further evidence that low socioeconomic status and living environment may influence the mycobiome. A few of our findings relating to particular fungal taxa are worthy of note. We suspect that higher abundance of *Cyberlindnera jadinii* (which is added to processed foods [41]) among US-born as compared with Latin American-born individuals may be associated with some aspect of diet. *Rhodotorula mucilaginosa*, a yeast species that can be found in the environment including within foods and beverages [42], was practically absent in the US-born members of our cohort; however, among those of Latin American birth, this species had mean abundance ~ 1% in the Caribbean-born groups (Cuba, Dominican Republic, Puerto Rico) and 2–3% in the Mexico-, Central America-, and South America-born groups. *R. mucilaginosa* is considered a rare although emerging human pathogen [42], and in the context of chronic disease, it is interesting for its carotenoid-producing potential [43]. Latin American-born individuals also had substantial mean abundance of several Candida species that were rare in the US-born, including *C. sake*, *C. glabrata*, and *C. tropicalis*. *C. tropicalis* is considered part of the normal human microbiota, yet it is of particular clinical interest for producing a virulent and sometimes antifungal-resistant systemic infection among patients in the Latin American and Asian regions [44]. Despite several interesting differences in the fungal distribution between US- and Latin American-born people, we were unable to identify particular fungal taxa that correlated significantly with obesity among US Hispanics.

Following seminal work in this area [9], we can point to several possible explanations for why exposure to the Latin American and US environments may be associated with distinct microbiota patterns. These may include conditions and mode of childbirth, breastfeeding, diet, functioning of the immune system due to pathogen exposures, and exposure to pets and livestock. In our study, lifestyle factor profiles including diet and socioeconomic status differed between the Latin American-born and US-born groups. Physical activity levels also varied across Hispanic groups, although this dimension of lifestyle was not found to be associated with GMB, an interesting null finding in light of prior studies showing GMB differences across more extreme contrasts of exercise habits [45]. Although several of these lifestyle factors were themselves associated with GMB, our multivariable adjustment models showed that lifestyle and socioeconomic variables did not explain the birthplace and migration associations with GMB or obesity risk. Nonetheless, despite the availability of a lengthy and wide-ranging in-person data collection protocol, it can be hard to exclude the influence of mismeasurement, unmeasured behaviors, or other environmental variables.

Over the short term, time-since-immigration effects on the GMB have been previously described in the USA [4]—is it plausible that the timing as well as the duration of US exposure may have independent effects? We speculate that the life course experience of childhood migrants from Latin America may have a particular influence on GMB. For instance, dramatic changes in diet, nutritional status, and environment after relocation to the USA may exert different effects when experienced in early life versus later adulthood. Thus, we might consider age-varying explanatory biological phenomena involving immunity, the physiology and function of the gastrointestinal tract, or social factors such as contacts with other US- and non-US-born individuals in the household. The time course for establishment of the adult microbiome pattern has been well studied (see [46]), although little is known about how age may alter the response to environmental perturbation (here represented as age at relocation from Latin America to the USA). In this regard, we note our prior report from the HCHS/SOL cohort that adults who were childhood migrants to the USA had higher prevalence of asthma as compared with both US-born individuals and adulthood migrants [47]. Like our GMB findings, these data on asthma are consistent with an immunological phenotype associated with early-life geographic relocation.

While we lacked a sufficient sample size to examine household clustering in this study [48, 49], in sensitivity analyses, we confirmed that key conclusions were similar after limiting the study to the subset of non-cohabitating individuals (data not shown). Other possible explanations which we may not have fully been able to control include differences across waves of migrant influx into the USA [50], as well as secular changes over time in the relevant environments (social, built, nutritional) of both the US and the Latin American source nations.

Limitations of this study include restriction to 16S and ITS1 sequencing. Shotgun metagenomic sequencing is in progress, which may allow identification of specific taxa down to the species and subspecies level, a necessary step to derive well-understood and modifiable biological targets. While we addressed the bacterial and fungal microbiome in parallel, interplay among bacterial and fungal taxa (co-occurrence, co-exclusion) will be complex to disentangle and will require larger samples and new statistical methods. Data on diet were assessed years prior to the GMB assessment, although we obtained these data using rigorous methods designed to capture habitual diet and showed strong associations between diet and GMB. Early-life environment was assessed retrospectively and subject to recall bias, suggesting that the relatively weak GMB signals in our data for variables such as childhood sanitation are likely to be underestimated. We did not study recent migrants because of the design of SOL, and geographic data was limited to the place of birth and the location of residence during the years of study participation. We also lacked repeated stool samples over time, and the analyses were cross-sectional, which will be overcome as the HCHS/SOL cohort members undergo future longitudinal assessments. Extant data suggest that genetic influences on the GMB are relatively weak and overshadowed by the environment [51, 52]. Hispanic background groups differ in average continental ancestry [53] yet we did not see a consistent pattern of difference by Hispanic background. Finally, only adults were studied, although results on migration suggest that studying children and adolescent migrant populations may capture a critical period for influences on lifelong GMB composition.

Strengths of the study setting include an extensive platform of clinical, biometric, behavioral, and sociodemographic variables which are of potential relevance to interactions among the host’s resident microbiome and the environment. Another design feature which lends credence to these comparisons was the approach of sampling all study participants from four US communities using random population-based recruitment methods and conducting assessments in a uniform manner across four US locations. The parent HCHS/SOL cohort had a relatively high participation rate of over 40%, which is notable considering that the cohort was inducted into a lengthy research program by door-to-door community recruitment. The participants were not selected from a diseased population, which allows us to address a large array of disease and biometric characteristics across a range of disease severity.

## Conclusions

In summary, this study shows that early-life migration and length of stay in mainland USA significantly affect key components of the GMB of Hispanic/Latino groups, which differ from other groups in the USA in microbiome features. In addition, obesity was associated with low bacterial alpha diversity consistent with other studies, but the findings of higher *Prevotella* to *Bacteroides* ratio in obese individuals was enigmatic suggesting a unique aspect of the GMB-host relationship in Latinos. This in turn suggests the hypothesis that particular aspects of the microbiome may explain unusual epidemiological patterns observed among the Latino community, such as high prevalence of diabetes, obesity, and asthma [47, 54, 55], concurrent with a paradoxical propensity for longevity [56].

## Methods

### Study cohort

HCHS/SOL is a prospective, population-based cohort study of 16,415 Hispanic/Latino adults (ages 18–74 years at the time of recruitment during 2008–2011) who were selected using a two-stage probability sampling design from randomly sampled census block areas within four US communities (Chicago, IL; Miami, FL; Bronx, NY; San Diego, CA) [57, 58]. The HCHS/SOL Gut Origins of Latino Diabetes (GOLD) ancillary study was conducted to examine the role of gut microbiome composition on diabetes and other outcomes, enrolling participants for this analysis from the HCHS/SOL approximately concurrent with the second in-person HCHS/SOL visit cycle (2014–2017). The study was conducted with the approval of the Institutional Review Boards (IRBs) of Albert Einstein College of Medicine, Feinberg School of Medicine at Northwestern University, Miller School of Medicine at the University of Miami, San Diego State University, and University of North Carolina at Chapel Hill. Written informed consent was obtained from all study participants.

### Participant characteristics and collection of clinical and behavioral data

A number of participant characteristics were ascertained by questionnaire at entry into HCHS/SOL, conducted by bilingual interviewers using the language preferred by the respondent. Self-reported variables included Hispanic/Latino background, place of birth, age at relocation (here termed “relocation age”), and years living in the mainland USA (with the US territory of Puerto Rico considered to be part of Latin America). Following previously described approaches, we used a combination of self-reported, objective monitoring, and clinical examination and blood laboratory components to define sociodemographic factors [59], medical history and medication use [60], physical activity including sedentary time and moderate-to-vigorous physical activity (MVPA) derived from 7-day hip worn accelerometry (Actical version B-1 model 198-0200-03; Respironics, Inc., Bend, OR) [61], and diet [62]. Sedentary time was classified according to quartiles, while MVPA was categorized according to whether participants met the 2008 US guidelines [63]. Diet variables were derived from the average of two 24-h dietary recalls that were collected at the HCHS/SOL baseline visit. The first recall was collected in person, and the second recall was collected by telephone within the following 3 months. Diet recalls were conducted using the Nutrition Data System for Research software (version 11) developed by the Nutrition Coordinating Center, University of Minnesota, (Minneapolis, Minnesota). Health insurance was defined according to participant self-report. Childhood economic hardship was assessed by the question, “Did your family ever experience a period of time when they had trouble paying for their basic needs, such as food, housing, medical care, and utilities, when you were a child? / Spanish: ¿Su familia alguna vez tuvo dificultades para pagar sus necesidades básicas como comidas, vivienda, cuidados médicos, o servicios públicos, cuando usted era niño(a)?” Access to sanitation during childhood was assessed by, “When you were growing up, did your home have the following basic utilities?... plumbing, septic tank. / Spanish: ¿Cuándo usted estaba creciendo, la casa donde vivía tenía los siguientes servicios públicos? Plomería, Drenaje/fosa séptica.” English or Spanish language preference was defined by the participant’s choice of English or Spanish written and spoken language in data collection encounters. Dietary acculturation was a self-reported measure stating whether a typical Hispanic, non-Hispanic (“American”), or blended style diet was consumed (“Of Hispanic/Latino and American food, do you usually eat...? Mainly or Mostly Hispanic/Latino foods” / Spanish: “De la comida hispana/latina y la comida americana, ¿por lo general come usted...? Principalmente comidas hispanas/latinas, or Mayormente comidas hispanas/latinas y algunas comidas americanas”.) We administered a modified 10-item version of the Short Acculturation Scale for Hispanics (SASH) which has 5-point Likert scale responses. The derived score for social acculturation was an average of the four SASH items regarding socialization practices and preferences [64]. Higher SASH response values represent greater acculturation to the dominant US culture. The overall SASH reliability was acceptable in the full sample (Cronbach’s *α* = .90), and for both English and Spanish language versions (*α*_English_ = .76; *α*_Spanish_ = .85). The reliability of SASH was similar across Hispanic/Latino background groups (ranging from *α*_South Americans_ = .85 to *α*_Mexicans_ = .89). In addition, the use of antibiotics or probiotic supplements and dietary preferences within the prior 6 months, as well as stool characteristics (Bristol scale), were ascertained via directed questions on self-administered questionnaire at the time of stool sample collection.

#### Stool sample collection and processing

Enrolled participants were provided with a stool collection kit. For each participant, a single fecal specimen was self-collected using a disposable paper inverted hat (Protocult collection device, ABC Medical Enterprises, Inc., Rochester, MN). Participants were instructed to collect a sample of the specimen with a plastic applicator attached to the cap, to place the applicator into a supplied container with a stabilizer (RNAlater, Invitrogen, Carlsbad, CA) and 0.5-mm-diameter glass beads, and then shake the container to mix stool and preservative [65]. Samples were shipped to Albert Einstein College of Medicine, aliquoted into 1-ml tubes and frozen at − 80 °C. Each aliquot was barcoded A–C and stored in a separate box.

The following method was used to randomize the samples sent to the Knight Lab for microbial sequencing. Using a team of three, three boxes were randomly selected from the set of all boxes containing the “A” sample using a random number generator. From a chosen box containing 81 samples, each person randomly selected three rows (9 tubes per row) of tubes and placed them randomly in one 96-well tube rack (1 rack per person; total 3 racks). The boxes were then rotated among the group, and the process was repeated twice resulting in three trays of 81 tubes consisting of 27 samples from each box. The process took less than 5 min and the tube racks were immediately returned to − 80 °C. The tubes from each rack were scanned in the randomized order creating a spreadsheet listing sample ID and location, placed in a new, labeled freezer box, and then returned to − 80 °C until shipment. Samples were shipped on dry ice via FedEx overnight delivery to the Knight lab for further analysis.

#### DNA extraction and sequencing

DNA extraction, 16S rRNA gene and ITS1 amplicon sequencing were done using Earth Microbiome Project (EMP) standard protocols (http://www.earthmicrobiome.org/protocols-and-standards/) [66]. Briefly, DNA was extracted with the Qiagen MagAttract PowerSoil DNA kit as previously described [67]. Amplicon polymerase chain reaction (PCR) was performed on the V4 region of the 16S rRNA gene using the primer pair 515f and 806r with Golay error-correcting barcodes on the reverse primer. Amplicon PCR was performed on the ITS1 region using primer pair ITS1f and ITS12 as described in the Earth Microbiome project (http://www.earthmicrobiome.org/protocols-and-standards/ITS1/). ITS1 amplicons were barcoded and pooled in equal concentrations for sequencing. The amplicon pool was purified with the MO BIO UltraClean PCR (Qiagen, Venlo, Netherlands) cleanup kit and sequenced on an Illumina MiSeq sequencing platform. Sequence data were demultiplexed and minimally quality filtered using the Quantitative Insights Into Microbial Ecology (QIIME) 1.9.1 [68] script split_libraries_fastq.py, with a PHRED quality threshold of 3 and default parameters to generate per-study FASTA sequence files.

### Bioinformatics processing and statistical analysis

Bioinformatic processing steps and statistical analyses were conducted in R versions 3.4.1 and 3.4.3 [69]. 16S sequence reads were clustered into operational taxonomic units (OTUs) based on ≥ 97% similarity by the UCLUST algorithm, matched against the GreenGenes reference database (version. 13_8) [70, 71]. Phylogenetic reconstruction was performed by PyNAST [72] with the information from the centroids of the reference sequence clusters contained in the GreenGenes reference database. Sequences that failed to align (e.g., chimeras) were removed. Data were then rarefied and subsampled to a coverage depth of 10,000 reads per sample for downstream analyses. Rarefaction curves are presented in Additional file 1: Figure S8.

For fungal bioinformatic processing, reads were trimmed for bases that fell below a PHRED score of 25 at the 3′ end with PrinSeq V0.20.4 [73]. DADA2 V1.8 [74] was used to pre-process the ITS1 sequencing and to remove chimeras using the default denovo protocol [74]. Processed reads were then clustered into amplicon sequencing variants using DADA2 and reference taxonomy was assigned using the naïve Bayesian classifier [75] and the UNITE reference database [76]. Outputs were imported into R using the phyloseq [77] package and further processed with vegan [78] and coin [79] packages.

16S rRNA gene V4 region (“16S”) amplicon sequencing [80, 81] was performed on 1920 samples with 142 samples being blank controls. The sequencing yielded 21,991 ± 12,087 (mean ± SD) reads per sample. After analysis with QIIME (version 1.9.1) closed reference OTU picking, there was an average of 20,624 ± 10,771 (mean ± SD) reads per sample. Of the 1778 participant samples, 1674 samples passed all QC metrics and were used in subsequent analyses. To evaluate the fungal component of the GMB, ITS1 amplification and sequencing were performed on the same samples resulting in 12,468 ± 41,628 reads per sample. Following DADA2 analysis, an average read count of 11,902 ± 36,170 reads per sample was obtained. Rarefaction analysis identified a stable plateau point at 500 reads which allowed 1028 samples to be used in subsequent analysis. PERMANOVA analysis using Bray-Curtis distances did not show any significant biases among four sequencing runs.

Taxonomic analyses were performed after collapsing OTUs at the genus level. Genera data were normalized with cumulative sum scaling (CSS) and log2 transformation to account for non-normal distribution [82]. The α-diversity (Shannon index) and β-diversity (Bray-Curtis distances) were calculated to investigate the community-level diversity of gut microbiota using phyloseq, vegan, and dada2 package in R (version 3.4.1) [77, 78]. Linear modeling was performed using the base R [25] *lm* function.

To identify correlates of GMB within the HCHS/SOL US Hispanic cohort, we used available information from the two in person HCHS/SOL study examinations as well as a brief diet, medication, and stool characteristic questionnaire that was collected at the time of GMB sampling. Lead correlates of beta diversity were identified by conducting PERMANOVA analysis of Bray-Curtis distances, computing the percent of sample clustering explained by 156 participant characteristics relating to stool quality, anthropometry (for example, height), behaviors (for example, diet), disease and use of medications (including clinical laboratory values, for example liver function tests), childhood exposures (including access to sanitation in home), sociocultural characteristics (including birthplace and relocation to the mainland USA), and demographic variables (sex, age). This set of variables was a subset of all collected variables available at the HCHS/SOL baseline and follow-up examinations, including those that had a plausible relationship with GMB and after selecting one out of every highly correlated set of variables. Pairwise correlations among included variables are shown in Additional file 1: Figure S9 and Additional file 1: Figure S10. The *adonis* function from the *vegan* package in R was used to assess statistical significance for PERMANOVA analyses. For simplicity, we used a single, uniform modeling approach for PERMANOVA analysis, using linear ordination across categories of independent (predictor) variables. This test was most sensitive to dose-response relationships between levels of the explanatory variable, and Bray-Curtis distance. To understand our results more fully, we also explored alternative statistical approaches including global differences among categories without assuming a dose-response ordination, which provided a more sensitive statistical test for variables such as relocation age which had a non-linear association with GMB metrics (data not shown). As expected, those variables rose in the *R*^2^ and *P* value rankings under the alternate modeling approach.

Using multivariable adjusted models, we isolated independent correlates of GMB outcomes. Linear modeling was performed using the base R [25] *lm* function with the dependent variable defined as the metrics of GMB including Shannon index, *Prevotella* to *Bacteroides* ratio, and the first two principal coordinates of Bray-Curtis distance. We performed log transformation as appropriate to improve model fit. We used the approaches of stratification combined with multivariable adjustment to address the relationship among multiple correlates of GMB in order to isolate associations with the variables of primary interest and exclude confounding. Adjustment variables were chosen based on a combination of empiric data on correlates of the main predictor and outcome variable, and knowledge of risk factor and disease relationships. These covariates included age (except for analyses with the primary predictor of interest defined as relocation age), gender, and study center for the initial adjusted models, and for the fully adjusted models, we added intake of vegetables without potatoes, intake of whole fruit, intake of whole grains, moderate-to-vigorous physical activity (continuous), BMI (six groups), diabetes/pre-diabetes/normoglycemic defined by American Diabetes Association criteria applied to study glucose and hemoglobin A1c levels (three groups), length and frequency of visits back to the participant’s country of origin (continuous), education level (four groups), income level (five groups), antibiotic use in the last 6 months (binary), and metformin use (binary). Next, in order to exclude confounding effects of age at the time of study, we examined the associations of relocation age with GMB across strata of current age at the time of GMB collection. This analysis was done after excluding individuals who relocated to the USA beyond age 26 years old in order to remove the strong correlation between relocation age and current age. A leave-one-out approach was also used to determine whether any single Hispanic background group was responsible for our main findings, and the Mexican subgroup of the HCHS/SOL was deemed large enough to allow analyses to be repeated in this group alone. To avoid false inferences due to small sample size, we excluded participant subgroups that had a small number of participants (for example, some of the mainland US-born groups separated out by Hispanic background). The final set of analyses examined the independent associations of GMB metrics and individual bacterial (16S) and fungal (ITS1) defined taxa with body mass index (obesity) and birthplace and migration. Significance testing followed a *P* < 0.05 criteria, and *q* values were used to control for multiple testing in R according to the method of Storey (http://github.com/jdstorey/qvalue).

## Supplementary information

**Additional file 1:Figure S1.** Age at relocation to the US among Latin America-born members of the HCHS/SOL cohort. **Figure S2.** Distribution of decade of relocation to the mainland US among Latin America-born members of the HCHS/SOL cohort. **Figure S3.** Association between age at relocation and current age in analyses restricted to individuals who relocated to the US before 26 years of age. **Figure S4.** Among Mexican/Mexican-American HCHS/SOL participants only, association of birthplace and acculturation related variables with bacterial 16S and fungal ITS1 gut microbiome features.** Figure S5.** Distribution of body mass index (BMI) categories, according to birthplace in the mainland US (50 states) and age at relocation from Latin America.** Figure S6.** Individual genera associated with obesity, birthplace and age at relocation to the mainland US. **Figure S7.** Rarefaction analysis for 16S rRNA and ITS1. **Figure S8.** Pairwise correlations among the top 35 predictor variables associated with Bray-Curtis distance for bacterial (16S) community. **Figure S9.** Pairwise correlations among the top 35 predictor variables associated with Bray-Curtis distance for fungal (ITS1) community.** Table S1. **Table of average relative abundance (%) for all species under Prevotella genus. **Table S2.** Definition of food group derived variables as determined from 24 hour dietary recalls. **Table S3. **Association between obesity and birthplace and age at relocation to the mainland US. **Table S4.** Association of genus level 16S data with obesity, adjusted for age, sex, field center and Hispanic background. **Table S5.** Association of genus level 16S data with age at relocation among Latin American born individuals, adjusted for age, sex, field center and Hispanic background. **Table S7.** Fungal taxa that differ between US born (USB) and Latin American born (LAB). **Table S8**. Association of genus level ITS1 data with obesity, adjusted for age, sex, field center and Hispanic background. **Table S9.** Association of genus level ITS1 data with age at relocation among Latin American born individuals, adjusted for age, sex, field center and Hispanic background.

**Additional file 2:Table S6.** Identified fungi from ITS1 sequencing.

**Additional file 3:** Review history.

## Data Availability

HCHS/SOL data are archived at the National Institutes of Health repositories dbGap and BIOLINCC. Sequence data from the samples described in this study have been deposited in QIITA, ID 11666, and EMBL-EBI ENA, ERP117287 [83]. HCHS/SOL has established a process for the scientific community to apply for access to participant data and materials, with such requests reviewed by the project’s Steering Committee. These policies are described at https://sites.cscc.unc.edu/hchs/ (accessioned September 15, 2019). The corresponding author will accept reasonable requests for data and specimen access, which will be referred to the Steering Committee of the HCHS/SOL project.
