# Author Correction: Gut microbiome composition in the Hispanic Community Health Study/Study of Latinos is shaped by geographic relocation, environmental factors, and obesity

**DOI:** 10.1186/s13059-020-01970-z

**Published:** 2020-02-25

**Authors:** Robert C. Kaplan, Zheng Wang, Mykhaylo Usyk, Daniela Sotres-Alvarez, Martha L. Daviglus, Neil Schneiderman, Gregory A. Talavera, Marc D. Gellman, Bharat Thyagarajan, Jee-Young Moon, Yoshiki Vázquez-Baeza, Daniel McDonald, Jessica S. Williams-Nguyen, Michael C. Wu, Kari E. North, Justin Shaffer, Christopher C. Sollecito, Qibin Qi, Carmen R. Isasi, Tao Wang, Rob Knight, Robert D. Burk

**Affiliations:** 1 Department of Epidemiology and Population Health, Albert Einstein College of Medicine, 1300 Morris Park Avenue, Bronx, NY 10461 USA; 2 Division of Public Health Sciences, Fred Hutchinson Cancer Research Center, Seattle, WA USA; 3 Department of Pediatrics, Albert Einstein College of Medicine, Bronx, NY USA; 4 Department of Biostatistics, Gillings School of Global Public Health, University of North Carolina, Chapel Hill, NC USA; 5 Institute for Minority Health Research, University of Illinois at Chicago College of Medicine, Chicago, IL USA; 6 Department of Psychology, University of Miami, Miami, FL USA; 7 Division of Health Promotion and Behavioral Science, San Diego State University, San Diego, CA USA; 8 Division of Molecular Pathology and Genomics, University of Minnesota, Minneapolis, MN USA; 9 Jacobs School of Engineering, University of California, San Diego, La Jolla, CA USA; 10 Center for Microbiome Innovation, University of California, San Diego, La Jolla, CA USA; 11 Department of Pediatrics, University of California, San Diego, La Jolla, CA USA; 12 Department of Epidemiology, Gillings School of Global Public Health, University of North Carolina, Chapel Hill, NC USA; 13 Department of Computer Science and Engineering, University of California, San Diego, La Jolla, CA USA; 14 Department of Bioengineering, University of California, San Diego, La Jolla, CA USA; 15 Department of Microbiology & Immunology, Albert Einstein College of Medicine, Bronx, NY USA

**Author Correction: Genome Biol (2019) 20:219**

**https://doi.org/10.1186/s13059-019-1831-z**

Following publication of the original paper [1], an error was reported in the third paragraph in the section “**Analysis of GMB composition and its correlates”** (page 3 of the PDF). The first sentence of the text should refer to Table 2, but mistakenly refers to Table 1.

The sentence should read:

“Fungal (ITS1) GMB populations were dominated by *Aspergillus proliferans* and *Saccharomyces cerevisiae* in both mainland US-born and Latin American-born groups (Table 2).”

As a result Table 2 appears further in the article, after the second reference to Table 2.
