# Supplementary material for: Gut microbiome composition in the Hispanic Community Health Study/Study of Latinos is shaped by geographic relocation, environmental factors, and obesity
Source: Genome Biol. 2019 Nov 1;20:219. doi: 10.1186/s13059-019-1831-z (PMC6824043; doi:10.1186/s13059-019-1831-z)
Supplement: Supplementary file 1 — Additional file 1:Figure S1. Age at relocation to the US among Latin America-born members of the HCHS/SOL cohort. Figure S2. Distribution of decade of relocation to the mainland US among Latin America-born members of the HCHS/SOL cohort. Figure S3. Association between age at relocation and current age in analyses restricted to individuals who relocated to the US before 26 years of age. Figure S4. Among Mexican/Mexican-American HCHS/SOL participants only, association of birthplace and acculturation related variables with bacterial 16S and fungal ITS1 gut microbiome features. Figure S5. Distribution of body mass index (BMI) categories, according to birthplace in the mainland US (50 states) and age at relocation from Latin America. Figure S6. Individual genera associated with obesity, birthplace and age at relocation to the mainland US. Figure S7. Rarefaction analysis for 16S rRNA and ITS1. Figure S8. Pairwise correlations among the top 35 predictor variables associated with Bray-Curtis distance for bacterial (16S) community. Figure S9. Pairwise correlations among the top 35 predictor variables associated with Bray-Curtis distance for fungal (ITS1) community. Table S1. Table of average relative abundance (%) for all species under Prevotella genus. Table S2. Definition of food group derived variables as determined from 24 hour dietary recalls. Table S3. Association between obesity and birthplace and age at relocation to the mainland US. Table S4. Association of genus level 16S data with obesity, adjusted for age, sex, field center and Hispanic background. Table S5. Association of genus level 16S data with age at relocation among Latin American born individuals, adjusted for age, sex, field center and Hispanic background. Table S7. Fungal taxa that differ between US born (USB) and Latin American born (LAB). Table S8. Association of genus level ITS1 data with obesity, adjusted for age, sex, field center and Hispanic background. Table S9. Association of genus level ITS1 da [file 13059_2019_1831_MOESM1_ESM.pdf]

Additional file 1

Supplemental results

Title: Gut microbiome composition in the Hispanic Community Health Study/Study of Latinos (HCHS/SOL) is shaped by geographic relocation, environmental factors and obesity

Authors: Robert C Kaplan[1, 2], Zheng Wang[1], Mykhaylo Usyk[3], Daniela Sotres-Alvarez[4], Martha Daviglus[5], Neil Schneiderman[6], Gregory A Talavera[7], Marc D Gellman[6], Bharat Thyagarajan[8], Jee-Young Moon[1], Yoshiki Vázquez -Baeza[10, 11], Daniel McDonald[9], Jessica S Williams-Nguyen[2], Michael C Wu[2], Kari E North[12], Justin Shaffer[9], Christopher C. Sollecito[3], Qibin Qi[1], Carmen R Isasi[1], Tao Wang[1], Rob Knight[9, 11, 13, 14], Robert D Burk[1,3, 15]

Corresponding author: Robert C Kaplan, PhD, Department of Epidemiology and Population Health, Albert Einstein College of Medicine, 1300 Morris Park Avenue, Bronx NY 10461 phone 718-430-4076. Email robert.kaplan@einstein.yu.edu

September 15, 2019

Fig S1. Age at relocation to the US among Latin America-born members of the HCHS/SOL cohort.

Fig S2. Distribution of decade of relocation to the mainland US among Latin America-born members of the HCHS/SOL cohort.

Fig S3. Association between age at relocation and current age in analyses restricted to individuals who relocated to the US before 26 years of age.

Fig S4. Among Mexican/Mexican-American HCHS/SOL participants only, association of birthplace and acculturation related variables with bacterial 16S and fungal ITS1 gut microbiome features.

Fig S5. Distribution of body mass index (BMI) categories, according to birthplace in the mainland US (50 states) and age at relocation from Latin America.

Fig S6. Individual genera associated with obesity, birthplace and age at relocation to the mainland US.

Fig S7. Rarefaction analysis for 16S rRNA and ITS1.

Fig S8. Pairwise correlations among the top 35 predictor variables associated with Bray-Curtis distance for bacterial (16S) community.

Fig S9. Pairwise correlations among the top 35 predictor variables associated with Bray-Curtis distance for fungal (ITS1) community.

Table S1. Table of average relative abundance (%) for all species under Prevotella genus

Table S2. Definition of food group derived variables as determined from 24 hour dietary recalls

Table S3. Association between obesity and birthplace and age at relocation to the mainland US.

Table S4. Association of genus level 16S data with obesity, adjusted for age, sex, field center and Hispanic background.

Table S5. Association of genus level 16S data with age at relocation among Latin American born individuals, adjusted for age, sex, field center and Hispanic background.

Table S6 is not contained in this file. See "Additional file 2.xls"

Table S7. Fungal taxa that differ between US born (USB) and Latin American born (LAB).

Table S8. Association of genus level ITS1 data with obesity, adjusted for age, sex, field center and Hispanic background.

Table S9. Association of genus level ITS1 data with age at relocation among Latin American born individuals, adjusted for age, sex, field center and Hispanic background.

Fig S1. Age at relocation to the US among Latin America-born members of the HCHS/SOL cohort.

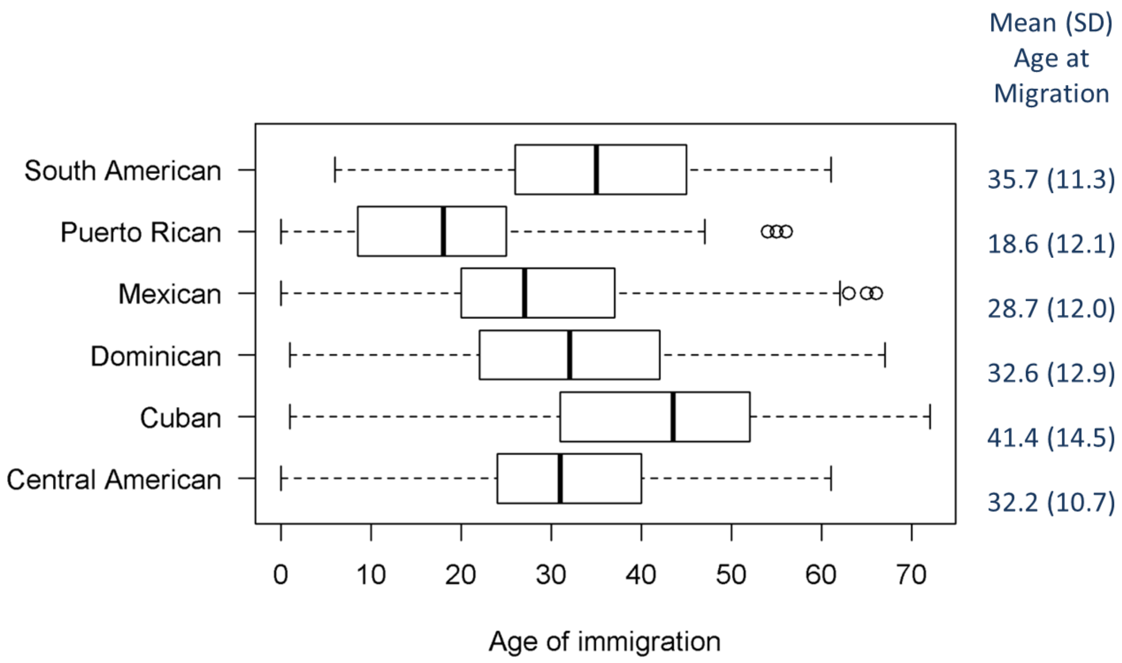

Fig S2. Distribution of decade of relocation to the mainland US among Latin America-born members of the HCHS/SOL cohort.

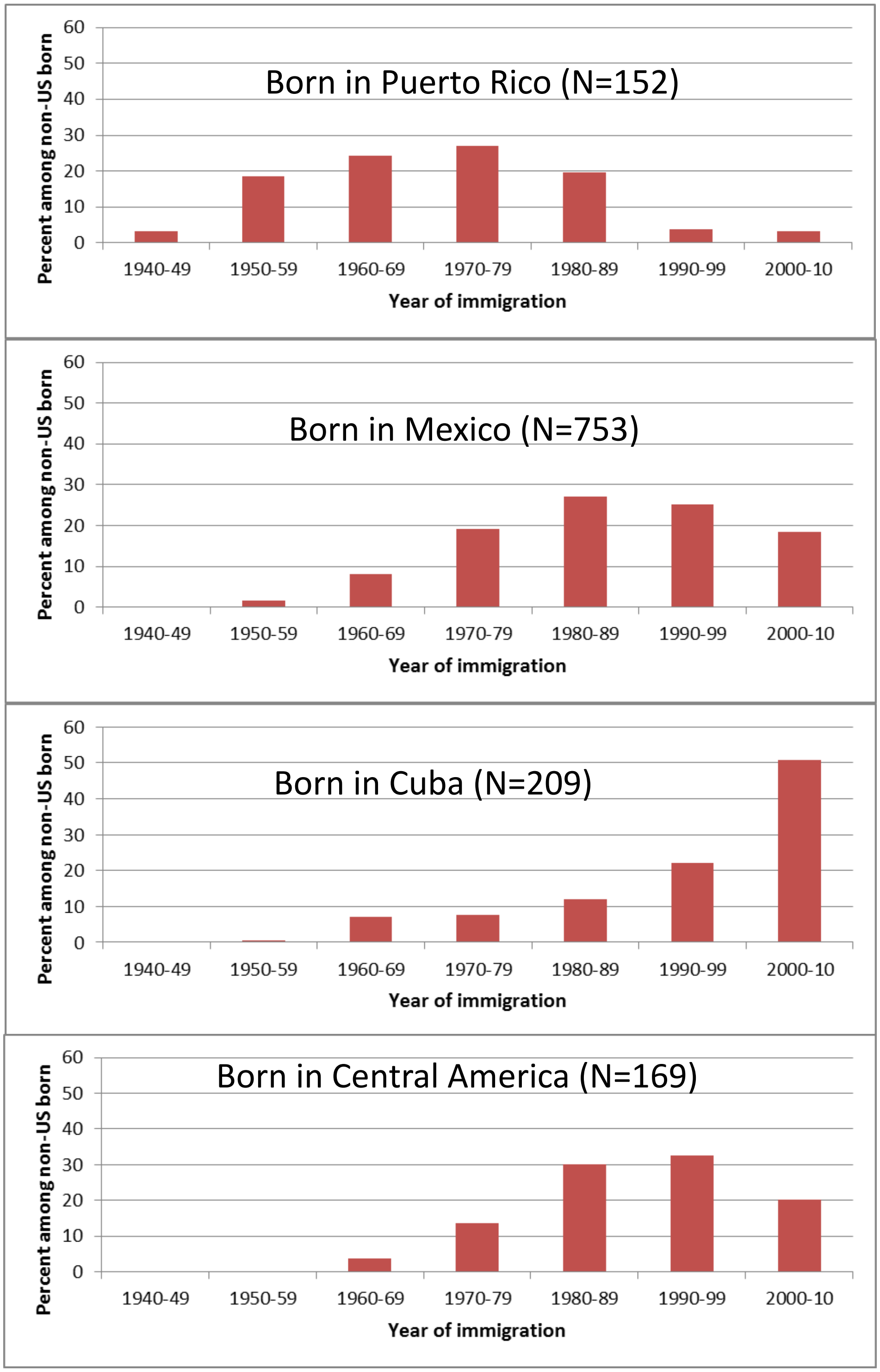

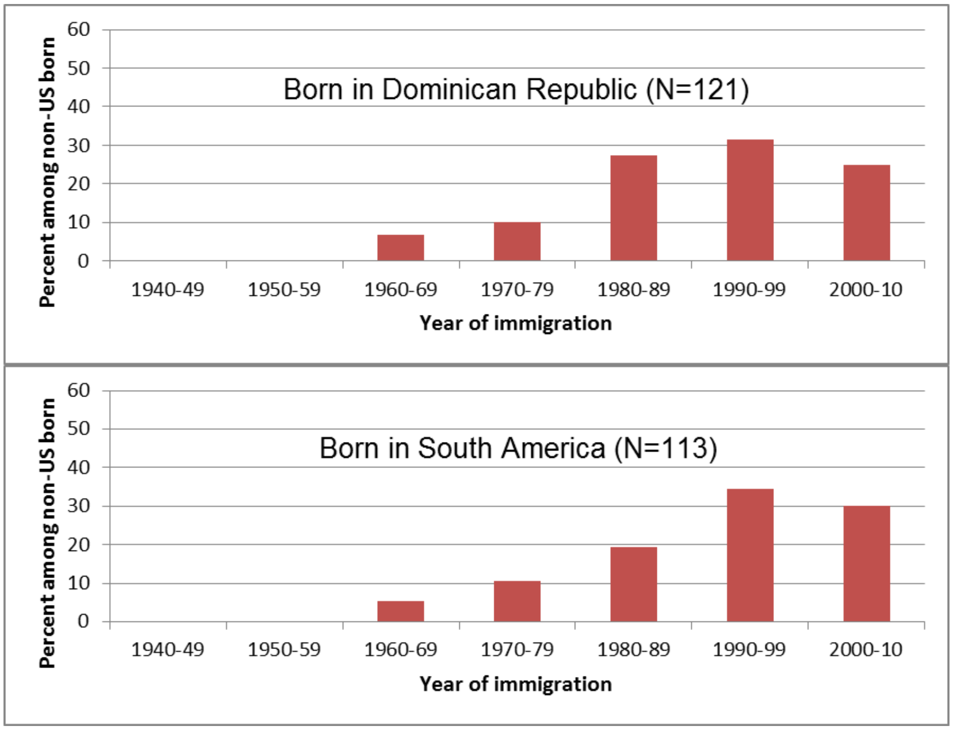
Born in Dominican Republic (N=121)
Percent among non-US born
60
50
40
30
20
10
0
1940-49
1950-59
1960-69
1970-79
1980-89
1990-99
2000-10
Year of immigration
Born in South America (N=113)
Percent among non-US born
60
50
40
30
20
10
0
1940-49
1950-59
1960-69
1970-79
1980-89
1990-99
2000-10
Year of immigration

Fig S3. Association between age at relocation and current age in analyses restricted to individuals who relocated to the US before 26 years of age. A. Scatterplot of age at relocation to the mainland US versus current age. Note that with those relocated after age 26 years (the minimum current age) having been excluded, there was no overall correlation between age at relocation and current age. B. Scatterplot of age at relocation to the mainland US versus current age among four age groups defined by attained age at the time of GMB sampling.

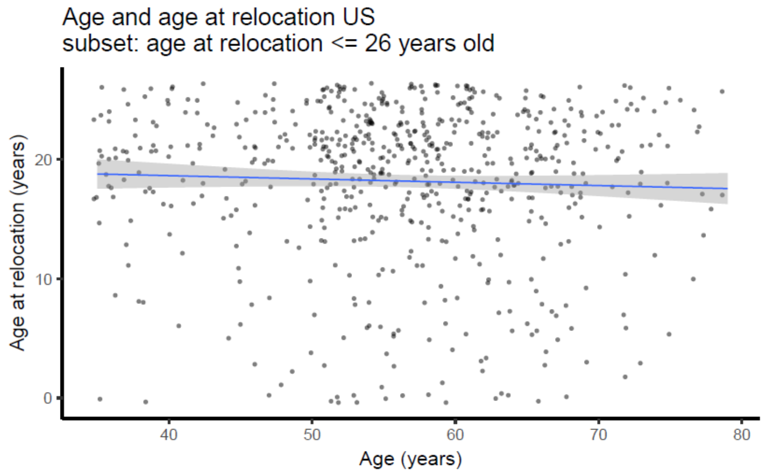

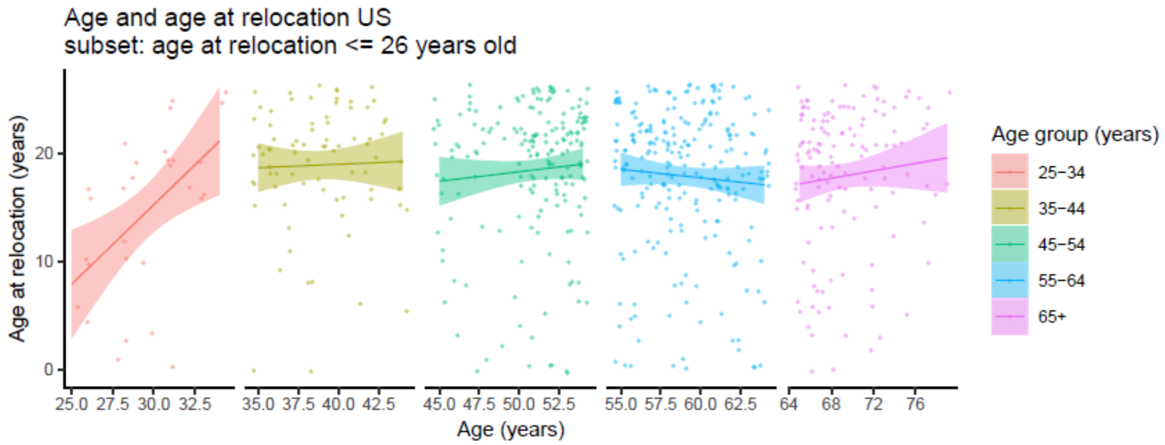

Fig S4. Among Mexican/Mexican-American HCHS/SOL participants only, association of birthplace and acculturation related variables with bacterial 16S and fungal ITS1 gut microbiome features.

A. Bacterial microbiome associations. Shown are the results of models adjusted for age (except for the model for age at relocation), sex, field center, intake of vegetables without potatoes, intake of whole fruit, intake of whole grains, moderate-to-vigorous physical activity (continuous), BMI (6 groups), diabetes (3 groups), length of visit to home country (continuous), education level (4 groups), income level (5 groups), antibiotic in last 6 months (binary), and metformin use (binary). Plot shows linear regression beta estimates and 95% confidence intervals for mean standardized gut microbiome outcomes. **B.** Fungal microbiome associations, analyzed in similar manner as described in A. PC1 and PC2, first and second principal components from principal coordinate analysis

A.

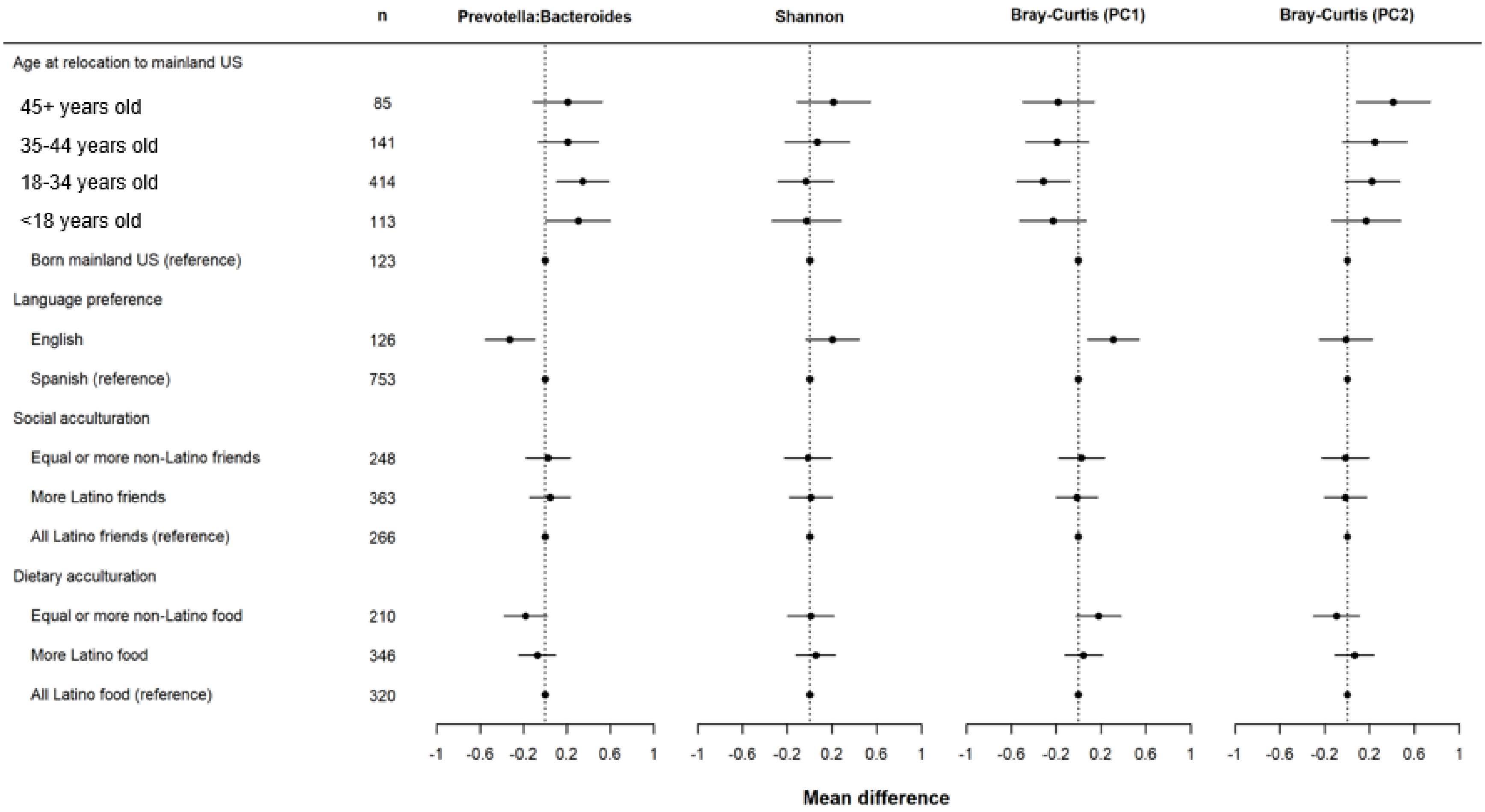

n
Prevotella:Bacteroides
Shannon
Bray-Curtis (PC1)
Bray-Curtis (PC2)
Age at relocation to mainland US
45+ years old 85
35-44 years old 141
18-34 years old 414
<18 years old 113
Born mainland US (reference) 123
Language preference
English 126
Spanish (reference) 753
Social acculturation
Equal or more non-Latino friends 248
More Latino friends 363
All Latino friends (reference) 266
Dietary acculturation
Equal or more non-Latino food 210
More Latino food 346
All Latino food (reference) 320
-1 -0.6 -0.2 0.2 0.6 1
Mean difference

B.

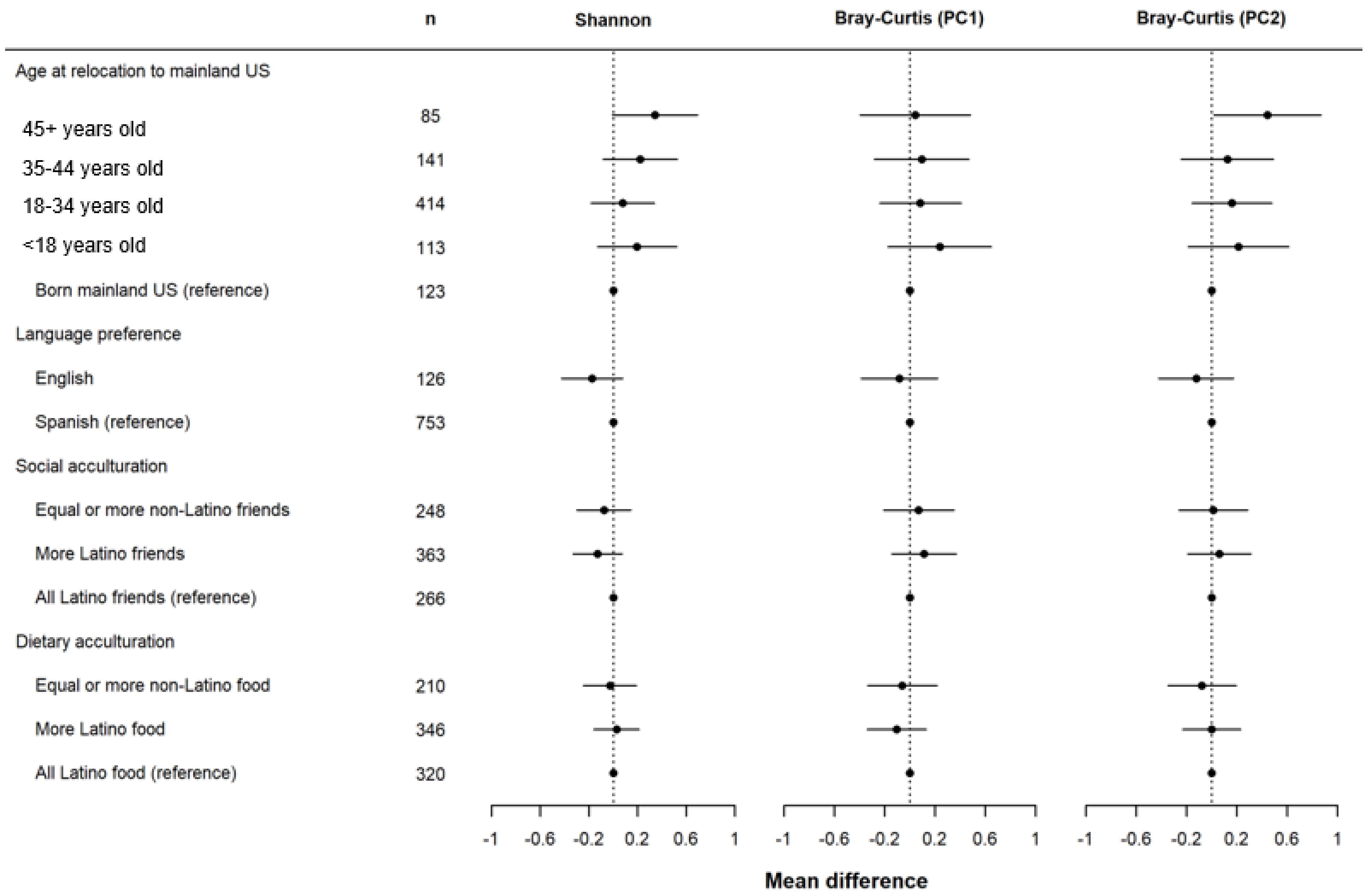

n
Shannon
Bray-Curtis (PC1)
Bray-Curtis (PC2)
Age at relocation to mainland US
45+ years old
85
35-44 years old
141
18-34 years old
414
<18 years old
113
Born mainland US (reference)
123
Language preference
English
126
Spanish (reference)
753
Social acculturation
Equal or more non-Latino friends
248
More Latino friends
363
All Latino friends (reference)
266
Dietary acculturation
Equal or more non-Latino food
210
More Latino food
346
All Latino food (reference)
320
-1
-0.6
-0.2
0.2
0.6
1
Mean difference

Fig S5. Distribution of body mass index (BMI) categories, according to birthplace in the mainland US (50 states) and age at relocation from Latin America. BMI defined as healthy range, 18.5 to 25 kg/m^2, overweight, 25 kg/m^2 to 30 kg/m^2, class I obesity, 30 kg/m^2 to 35 kg/m^2class II obesity, 35 kg/m^2 to 40 kg/m^2, and class III obesity, BMI above 40 kg/m^2.

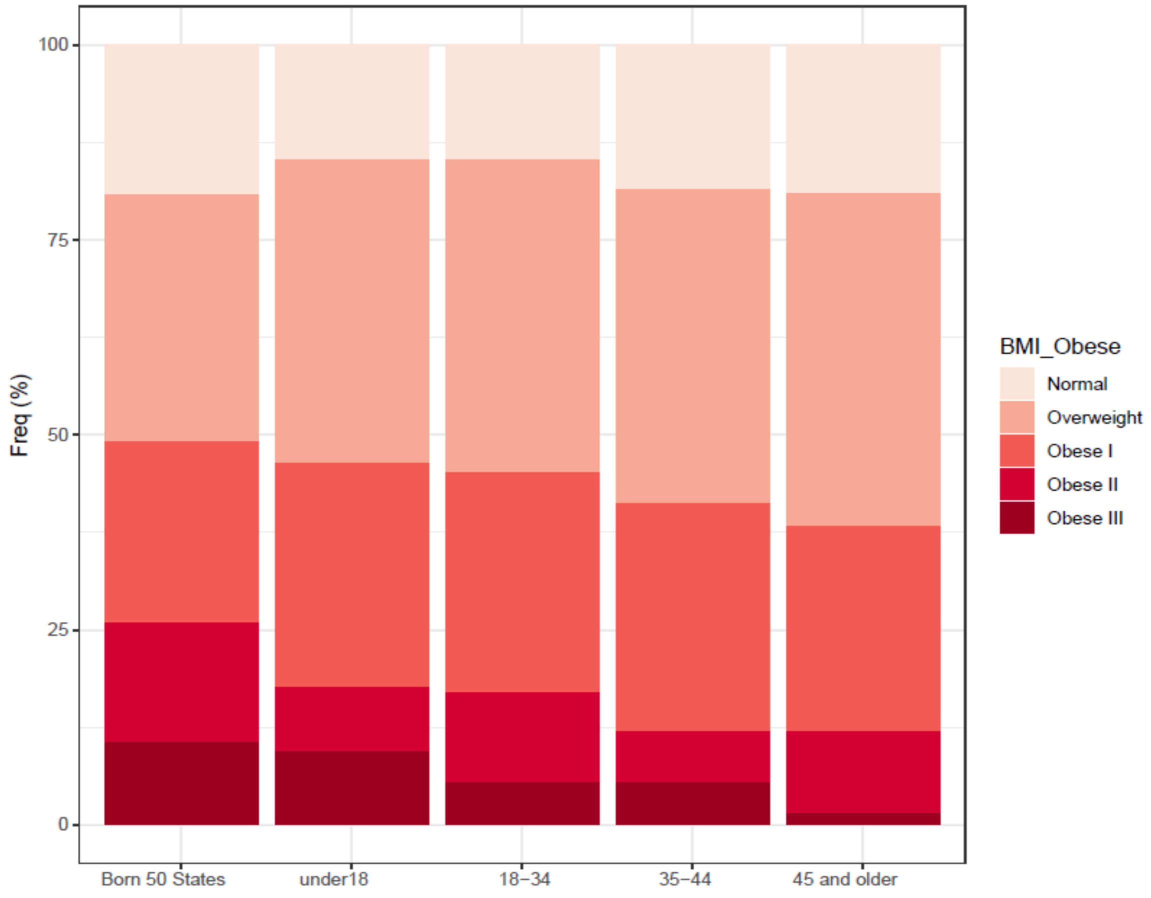

Fig S6. Individual genera associated with obesity, birthplace and age at relocation to the mainland US. A. *Oscillospira* **B.** *Unclassified.Clostridiales* **C.** *Unclassified.Ruminococcaceae* **D.** *Unclassified.Christensenellaceae* **E.** *Unclassified.Coriobacteriaceae* **F.** *Megasphaera* **G.** *Acidaminococcus* **H.** *Anaerotruncus* **I.** *Unclassified.Victivallaceae* **J.** *Unclassified.YS2*

A.

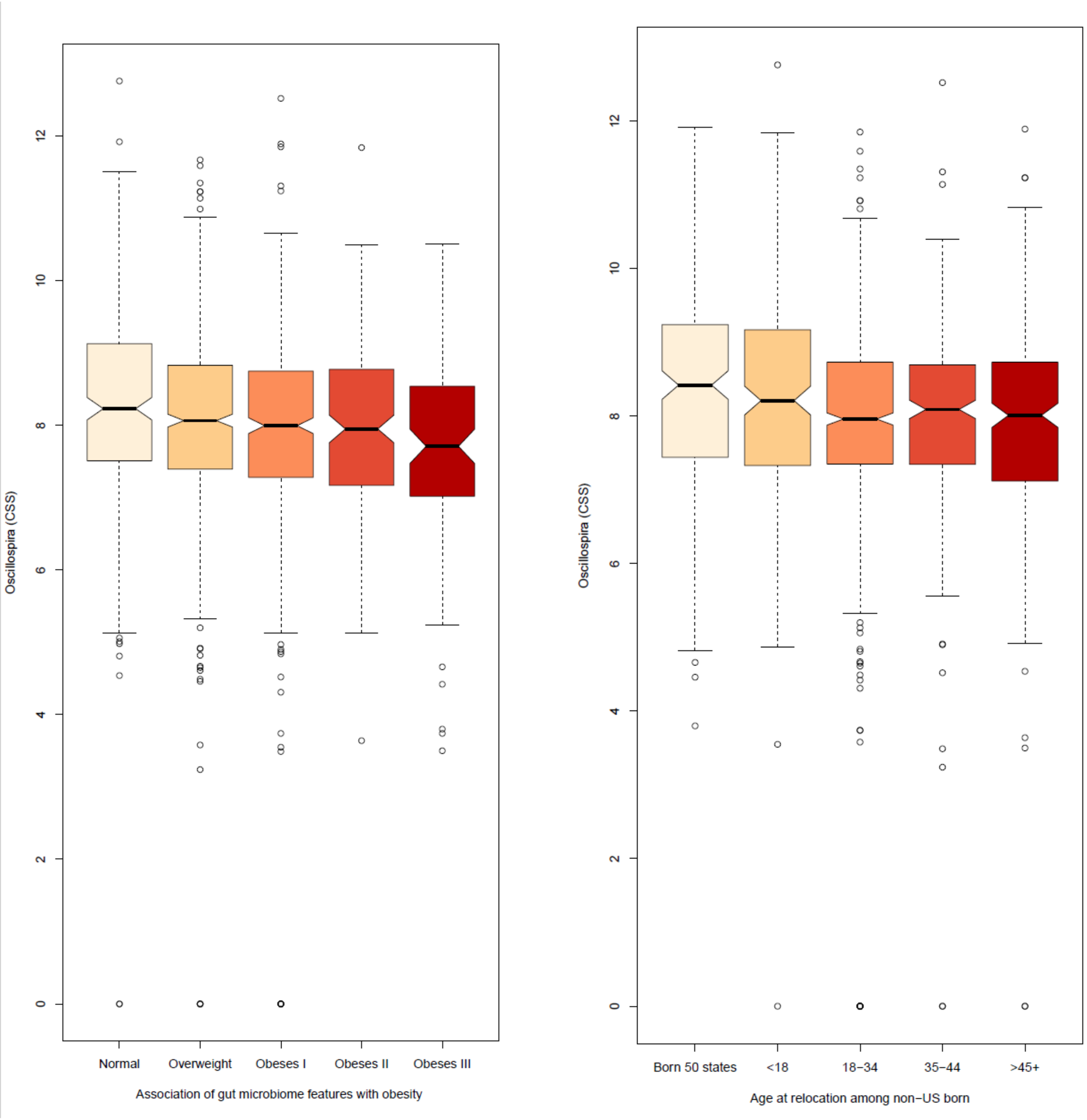

Oscillospira (CSS)
0
2
4
6
8
10
12
Normal
Overweight
Obeses I
Obeses II
Obeses III
Association of gut microbiome features with obesity
Oscillospira (CSS)
0
2
4
6
8
10
12
Born 50 states
<18
18−34
35−44
>45+
Age at relocation among non−US born

B.

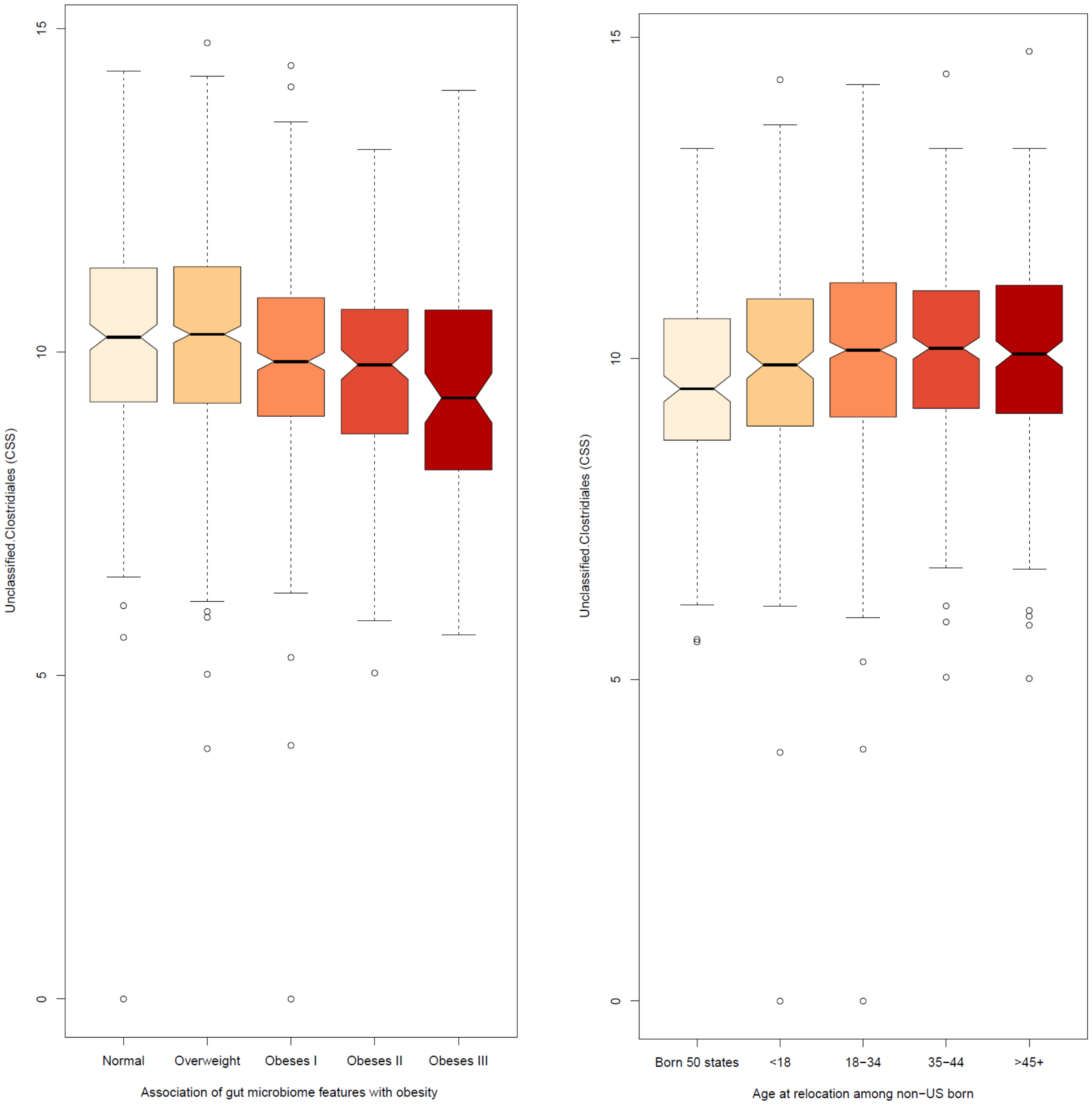

Unclassified.Clostridiales (CSS)
0
5
10
15
Normal
Overweight
Obeses I
Obeses II
Obeses III
Association of gut microbiome features with obesity
Unclassified.Clostridiales (CSS)
0
5
10
15
Born 50 states
<18
18−34
35−44
>45+
Age at relocation among non−US born

C.

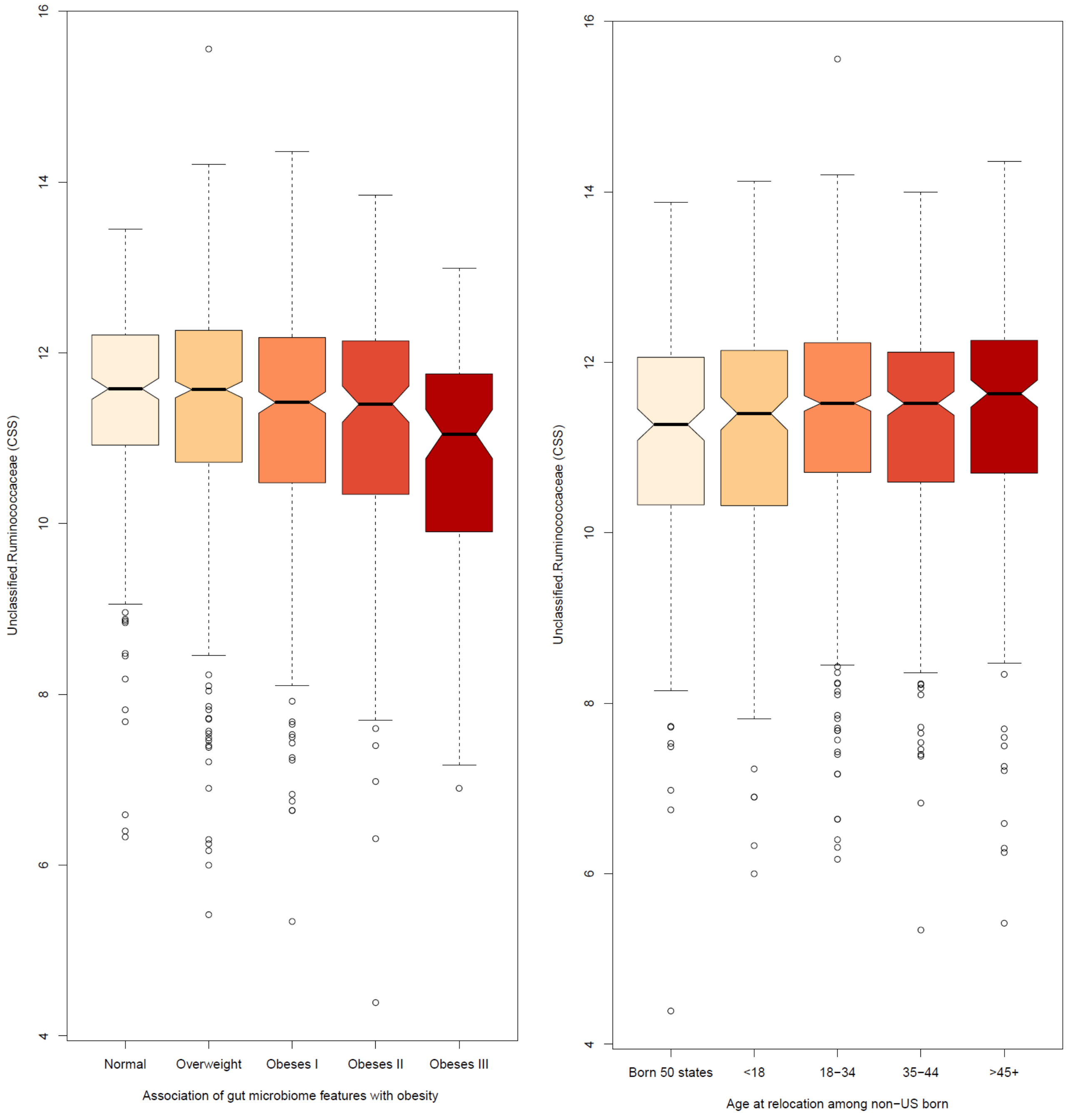

16
14
12
10
8
6
4
Unclassified.Ruminococcaceae (CSS)
Normal
Overweight
Obeses I
Obeses II
Obeses III
Association of gut microbiome features with obesity
Born 50 states
<18
18−34
35−44
>45+
Age at relocation among non−US born

D.

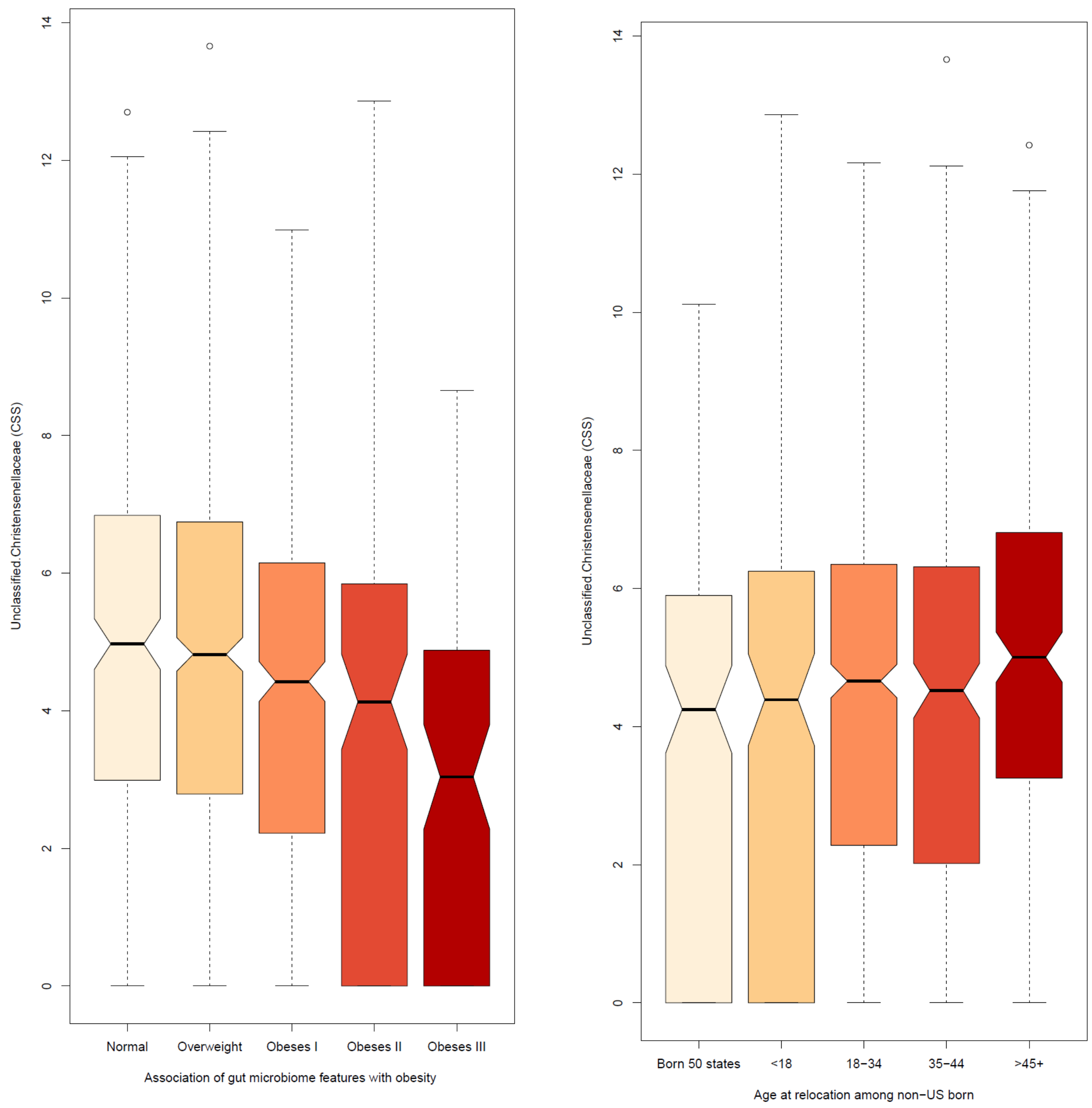

Unclassified.Christensenellaceae (CSS)
0
2
4
6
8
10
12
14
Normal
Overweight
Obeses I
Obeses II
Obeses III
Association of gut microbiome features with obesity
Unclassified.Christensenellaceae (CSS)
Born 50 states
<18
18−34
35−44
>45+
Age at relocation among non−US born

E.

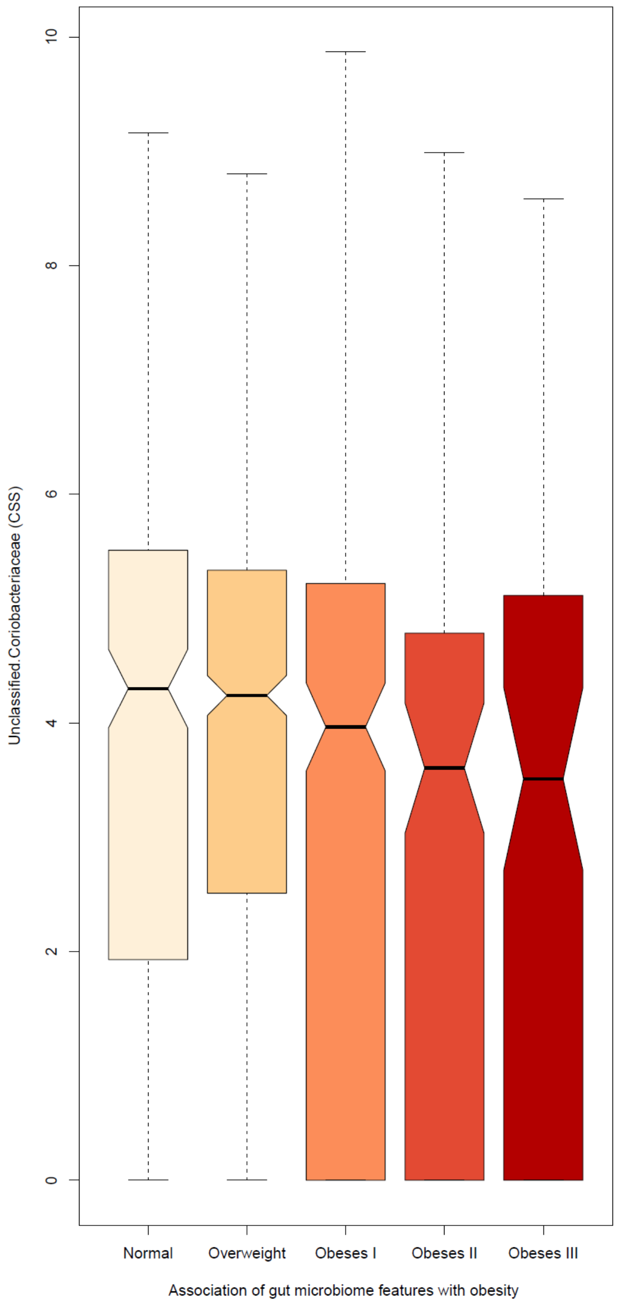

Unclassified.Coriobacteriaceae (CSS)
0
2
4
6
8
10
Normal
Overweight
Obeses I
Obeses II
Obeses III
Association of gut microbiome features with obesity

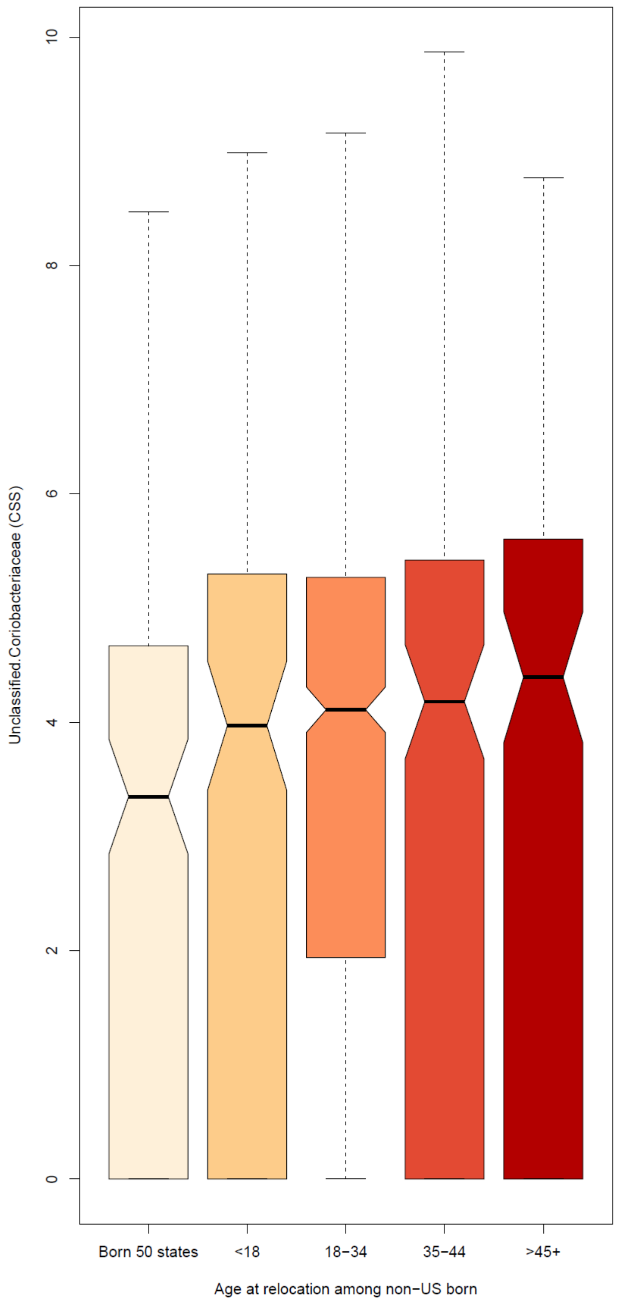

Unclassified.Coriobacteriaceae (CSS)
0
2
4
6
8
10
Born 50 states
<18
18−34
35−44
>45+
Age at relocation among non−US born

F.

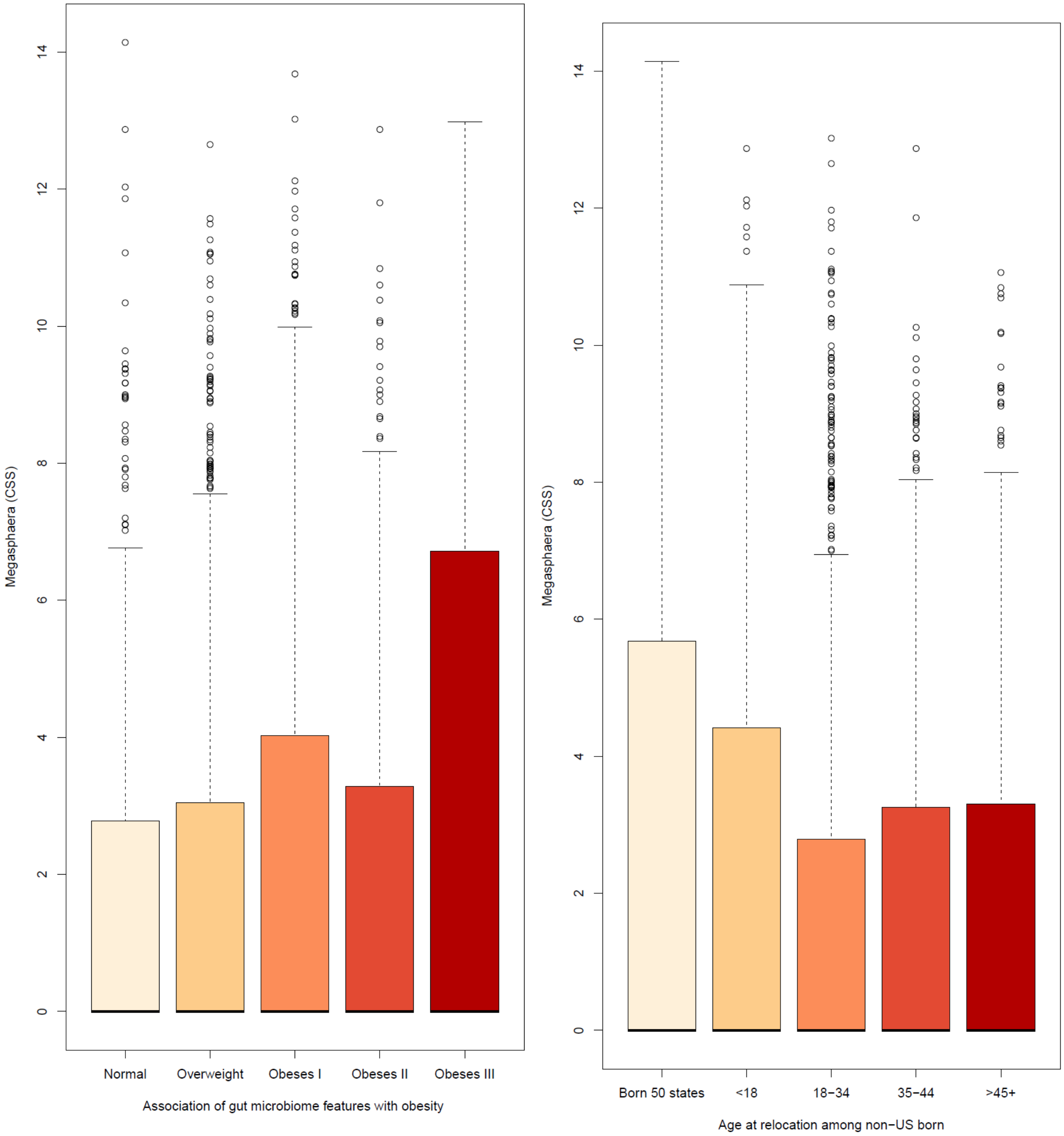

Megasphaera (CSS)
0
2
4
6
8
10
12
14
Normal
Overweight
Obeses I
Obeses II
Obeses III
Association of gut microbiome features with obesity
Megasphaera (CSS)
0
2
4
6
8
10
12
14
Born 50 states
<18
18−34
35−44
>45+
Age at relocation among non−US born

G.

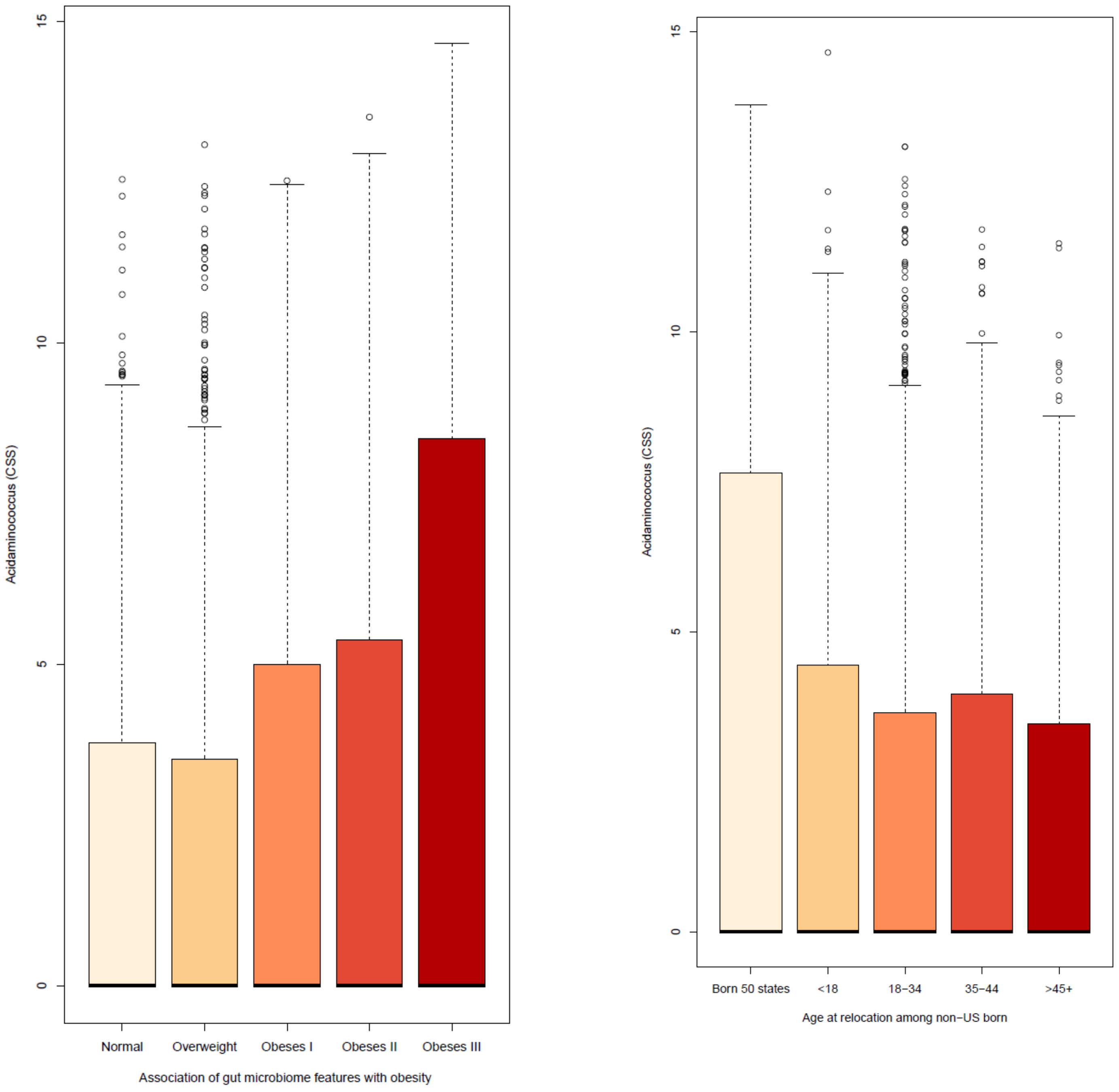

Acidaminococcus (CSS)
0
5
10
15
Normal
Overweight
Obeses I
Obeses II
Obeses III
Association of gut microbiome features with obesity
Acidaminococcus (CSS)
0
5
10
15
Born 50 states
<18
18−34
35−44
>45+
Age at relocation among non−US born

H.

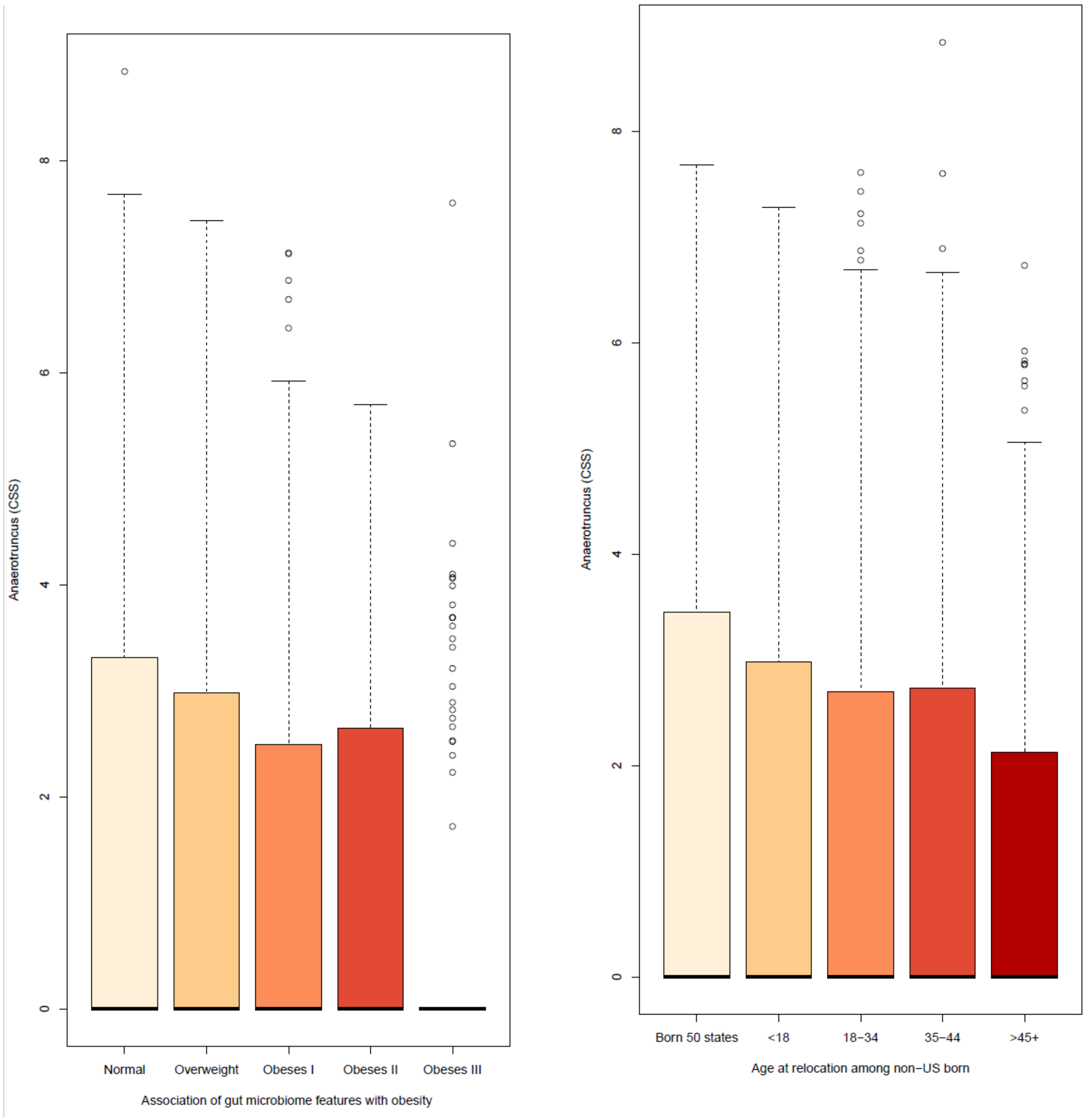
Anaerotruncus (CSS)
0
2
4
6
8
Normal
Overweight
Obeses I
Obeses II
Obeses III
Association of gut microbiome features with obesity
Anaerotruncus (CSS)
0
2
4
6
8
Born 50 states
<18
18−34
35−44
>45+
Age at relocation among non−US born

I.

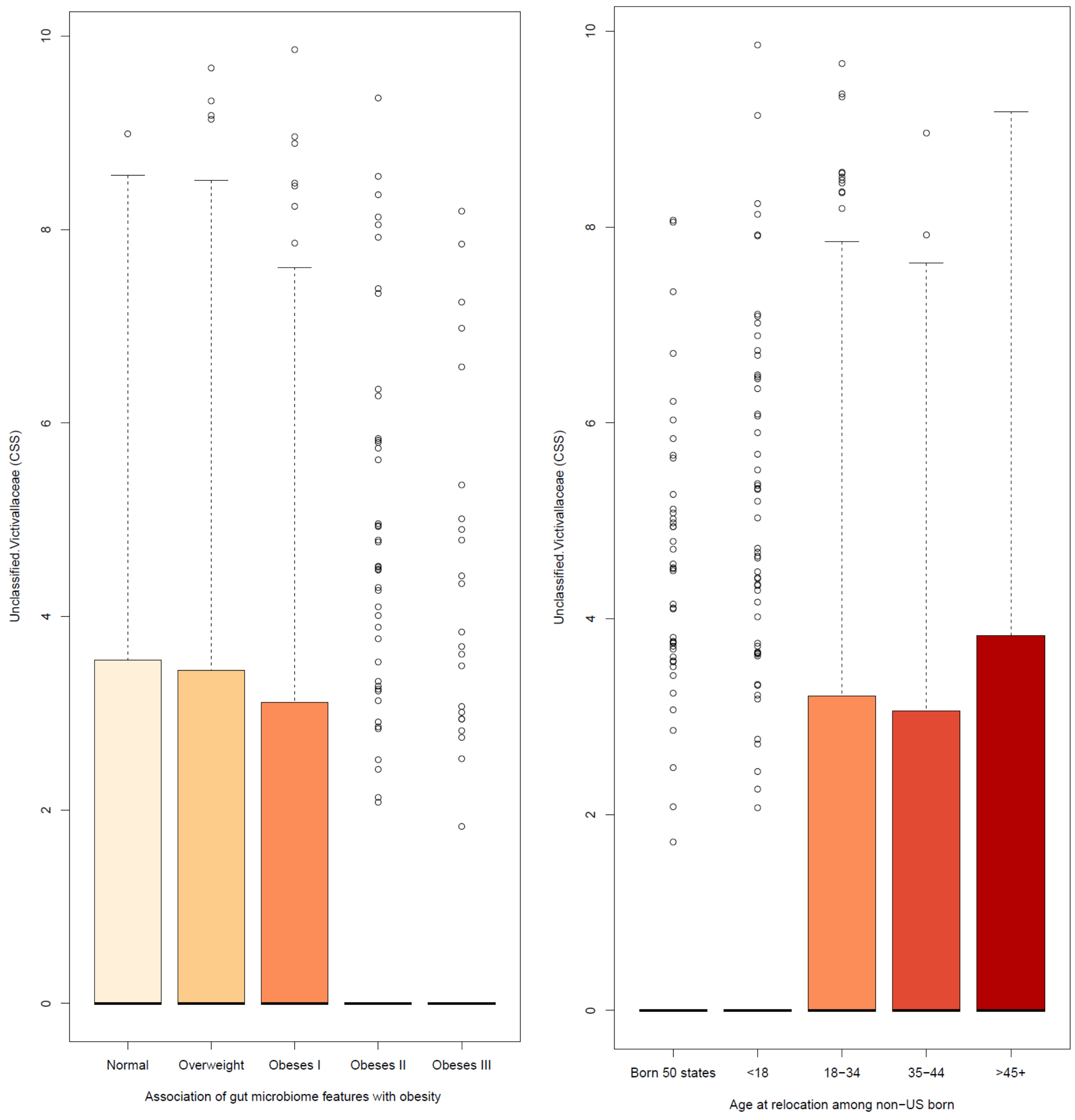

Unclassified.Victivallaceae (CSS)
0
2
4
6
8
10
Normal
Overweight
Obeses I
Obeses II
Obeses III
Association of gut microbiome features with obesity
Unclassified.Victivallaceae (CSS)
0
2
4
6
8
10
Born 50 states
<18
18−34
35−44
>45+
Age at relocation among non−US born

J.

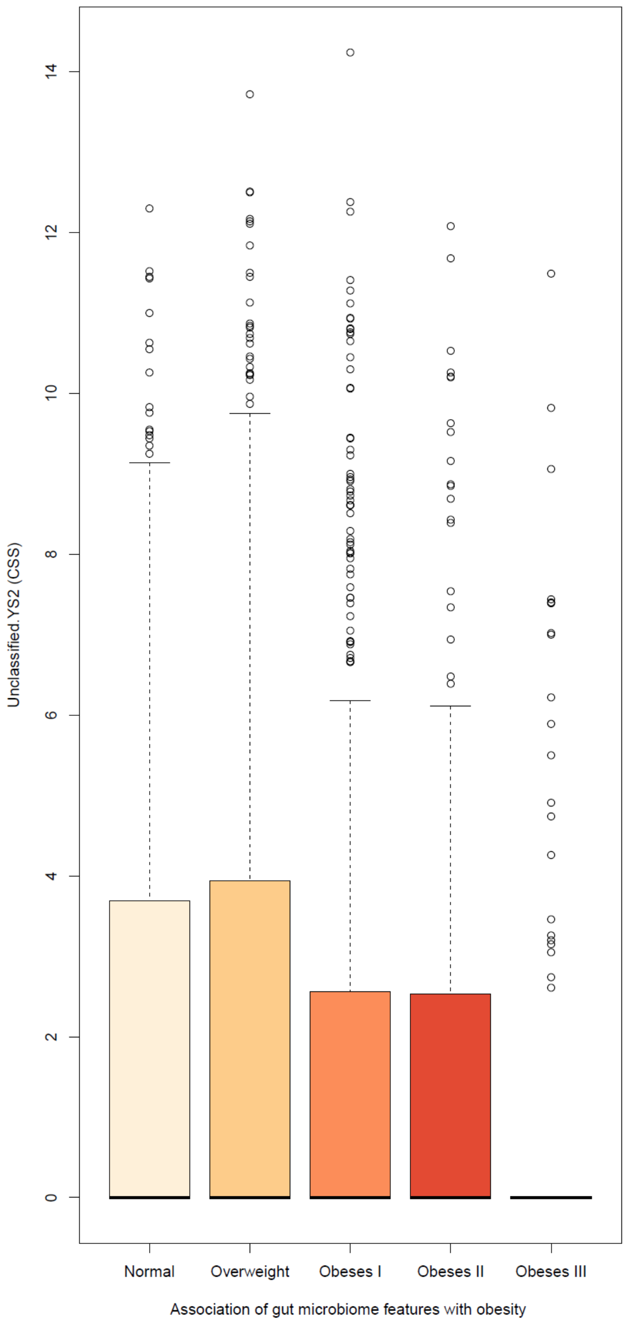

Unclassified.YS2 (CSS)
0
2
4
6
8
10
12
14
Normal
Overweight
Obeses I
Obeses II
Obeses III
Association of gut microbiome features with obesity

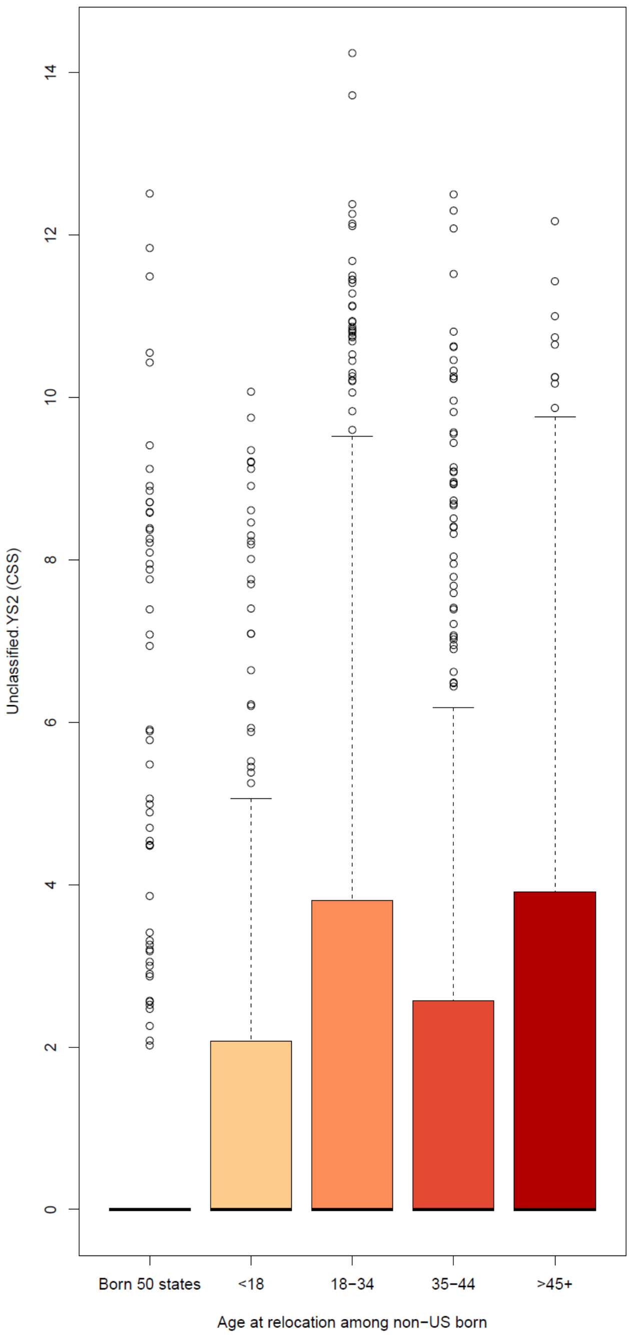

Unclassified.YS2 (CSS)
0
2
4
6
8
10
12
14
Born 50 states
<18
18−34
35−44
>45+
Age at relocation among non−US born

Fig S7. Rarefaction analysis for 16S rRNA (top) and ITS1 (bottom).

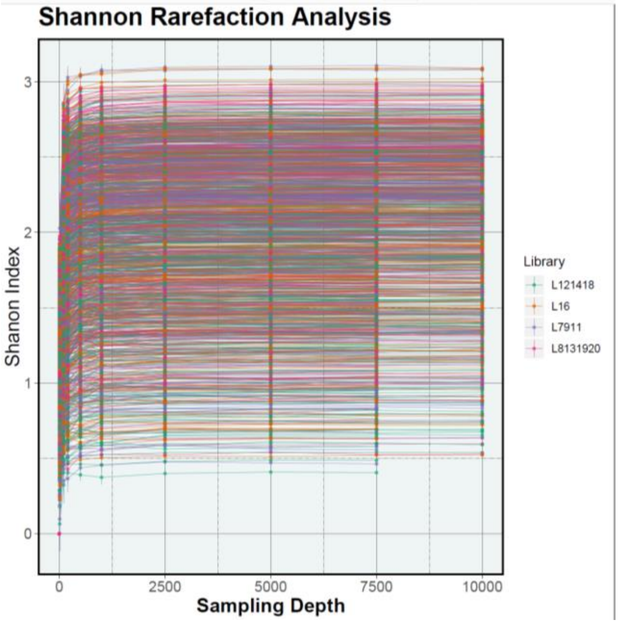

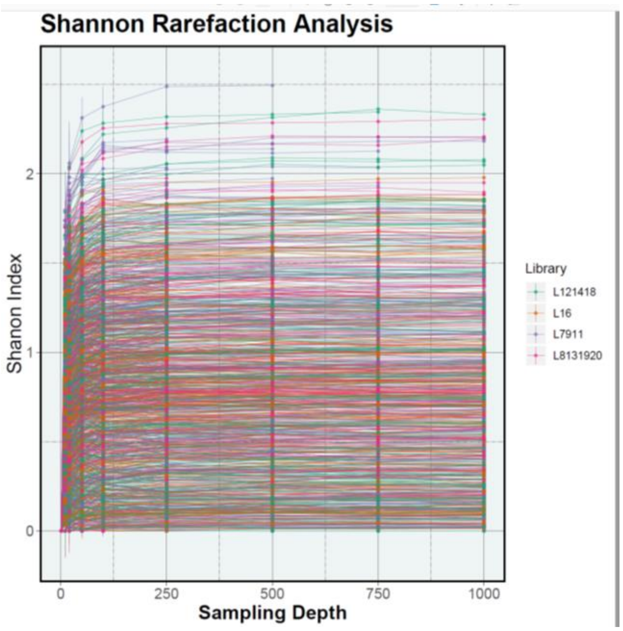

Fig S8. Pairwise correlations among the top 35 predictor variables associated with Bray-Curtis distance for bacterial (16S) community.

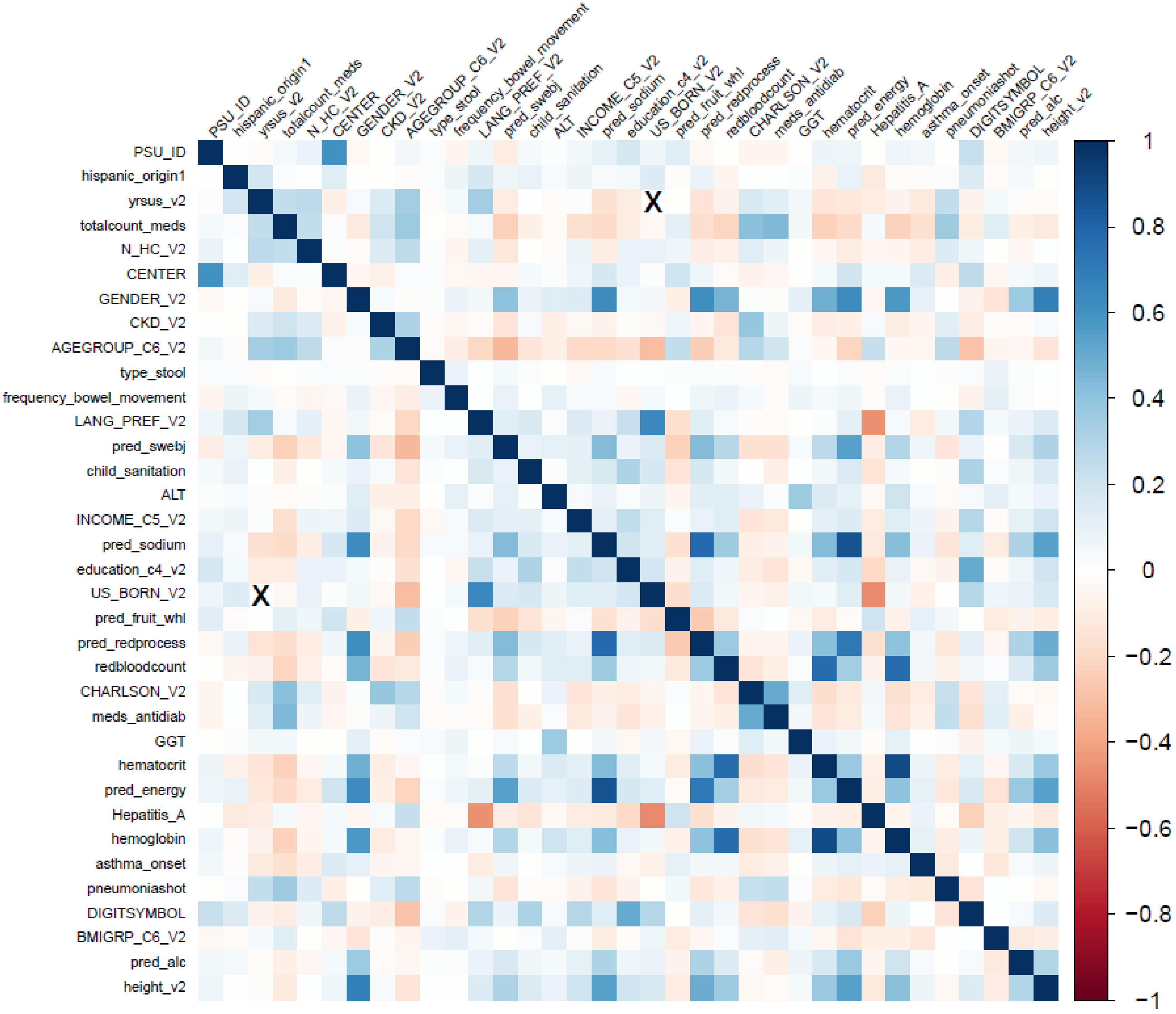

Fig S9. Pairwise correlations among the top 35 predictor variables associated with Bray-Curtis distance for fungal (ITS1) community.

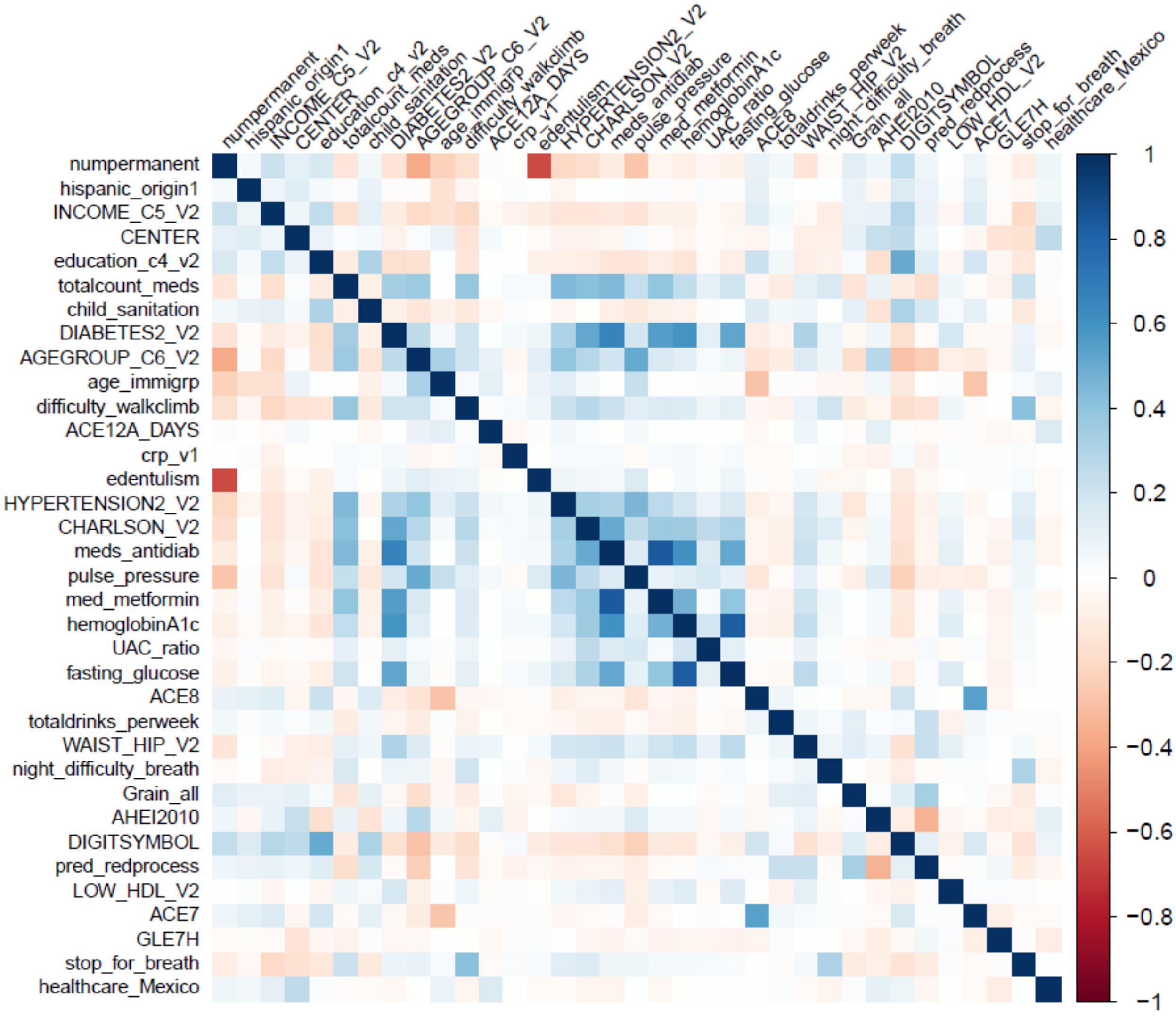

Table S1. Table of average relative abundance (%) for all species under Prevotella genus

Taxa	Prevotella_copri	Unclassified. Prevotella	Prevotella_stercorea	Prevotella_nigrescens	Prevotella_nanceiensis	Prevotella_melaninogenica	Prevotella_intermedia
Average Relative abundance (%)	23.18	1.77	1.17	2.99E-05	1.19E-04	4.54E-04	4.71E-03
Percentage in Prevotella genus (%) ; Note: Prevotella genus =100%	88.72	6.78	4.49	1.14E-04	4.57E-04	1.74E-03	0.02

Table S2. Definition of food group derived variables as determined from 24 hour dietary recalls

Food group variable	HCHS/SOL source variable name	Source variable description
FRUIT	DTSA4	FRU0100 Citrus Juice
	DTSA6	FRU0300 Citrus Fruit
	DTSA5	FRU0200 Fruit Juice excluding Citrus Juice
	DTSA7	FRU0400 Fruit excluding Citrus Fruit
	DTSA8	FRU0500 Avocado and Similar
	DTSA9	FRU0600 Fried Fruits
	DTSA10	FRU0700 Fruit-based Savory Snack
VEGETABLE WITHOUT POTATO	DTSA11	VEG0100 Dark-green Vegetables
	DTSA12	VEG0200 Deep-yellow Vegetables
	DTSA13	VEG0300 Tomato
	DTSA16	VEG0450 Other Starchy Vegetables
	DTSA18	VEG0600 Other Vegetables
	DTSA20	VEG0500 Vegetable Juice
GRAIN (REFINED)	DTSA23	GRS0100 Grains, Flour and Dry Mixes - Some Whole Grain
	DTSA24	GRR0100 Grains, Flour and Dry Mixes - Refined Grain
	DTSA26	GRS0200 Loaf-type Bread and Plain Rolls - Some Whole Grain
	DTSA27	GRR0200 Loaf-type Bread and Plain Rolls - Refined Grain
	DTSA29	GRS0300 Other Breads (quick breads, corn muffins, tortillas) - Some Whole Grain
	DTSA30	GRR0300 Other Breads (quick breads, corn muffins, tortillas) - Refined Grain
	DTSA32	GRS0400 Crackers - Some Whole Grain
	DTSA33	GRR0400 Crackers - Refined Grain
	DTSA35	GRS0500 Pasta - Some Whole Grain

	DTSA36	GRR0500 Pasta - Refined Grain
	DTSA38	GRS0600 Ready-to-eat Cereal (not presweetened) - Some Whole Grain
	DTSA39	GRR0600 Ready-to-eat Cereal (not presweetened) - Refined Grain
	DTSA41	GRS0700 Ready-to-eat Cereal (presweetened) - Some Whole Grain
	DTSA42	GRR0700 Ready-to-eat Cereal (presweetened) - Refined Grain
	DTSA44	GRS0800 Cakes, Cookies, Pies, Pastries, Danish, Doughnuts and Cobblers - Some Whole Grain
	DTSA45	GRR0800 Cakes, Cookies, Pies, Pastries, Danish, Doughnuts and Cobblers - Refined Grain
	DTSA47	GRS1000 Snack Bars - Some Whole Grain
	DTSA48	GRR1000 Snack Bars - Refined Grain
	DTSA50	GRS0900 Snack Chips - Some Whole Grain
	DTSA51	GRR0900 Snack Chips - Refined Grain
	DTSA53	GRW1200 Flavored Popcorn
	DTSA54	GRO0100 Baby Food Grain Mixtures
GRAIN (WHOLE)	DTSA22	GRW0100 Grains, Flour and Dry Mixes - Whole Grain
	DTSA25	GRW0200 Loaf-type Bread and Plain Rolls - Whole Grain
	DTSA28	GRW0300 Other Breads (quick breads, corn muffins, tortillas) - Whole Grain
	DTSA31	GRW0400 Crackers - Whole Grain
	DTSA34	GRW0500 Pasta - Whole Grain
	DTSA37	GRW0600 Ready-to-eat Cereal (not presweetened) - Whole Grain
	DTSA40	GRW0700 Ready-to-eat Cereal (presweetened) - Whole Grain
	DTSA43	GRS0700 Ready-to-eat Cereal (presweetened) - Some Whole Grain
	DTSA46	GRW1000 Snack Bars - Whole Grain
	DTSA49	GRW0900 Snack Chips - Whole Grain
	DTSA52	GRW1100 Popcorn
RED AND PROCESSED MEATS	DTSA55	MRF0100 Beef
	DTSA56	MRL0100 Lean Beef
	DTSA57	MRF0200 Veal
	DTSA58	MRL0200 Lean Veal
	DTSA59	MRF0300 Lamb

	DTSA60	MRL0300 Lean Lamb
	DTSA61	MRF0400 Fresh Pork
	DTSA62	MRL0400 Lean Fresh Pork
	DTSA65	MRF0500 Game
	DTSA63	MCF0200 Cured Pork
	DTSA64	MCL0200 Lean Cured Pork
	DTSA74	MCF0100 Cold Cuts and Sausage
	DTSA75	MCL0100 Lean Cold Cuts and Sausage
LEGUMES AND NUTS	DTSA17	VEG0700 Legumes (cooked dried beans)
	DTSA81	MOF0500 Nuts and Seeds
	DTSA82	MOF0600 Nut and Seed Butters
SUGAR SWEETENED BEVERAGES	DTSA134	BVS0400 Sweetened Soft Drinks
	DTSA137	BVS0300 Sweetened Fruit Drinks
	DTSA139	BVS0500 Sweetened Tea
	DTSA142	BVS0100 Sweetened Coffee
	DTSA145	BVS0200 Sweetened Coffee Substitutes
	DTSA148	BVS0600 Sweetened Water
	DTSA151	BVS0700 Nondairy-based Sweetened Meal Replacement/Supplement
	DTSA154	BVO0100 Non-alcoholic Beer
	DTSA155	BVO0200 Non-alcoholic Light Beer

Table S3. Association between obesity and birthplace and age at relocation to the mainland US. Obesity is defined according to body mass index of defined as obesity, above 30 kg/m^2, and class II - III obesity, above 35 kg/m^2 , versus the normal-weight group of body mass index 18.5 to 25 kg/m^2. CI, confidence interval.

	Obesity		Class II-III Obesity	
	OR (95% CI)	p-value	OR (95% CI)	p-value
Place of birth		0.03		0.006
Mainland US Born (REFERENCE GROUP)	1		1	
Latin American Born	0.56 (0.32- 0.96)		0.42 (0.19-0.95)	
Age at relocation to mainland US		0.05		0.0001
Mainland US Born	2.28 (1.22- 4.27)		4.62 (1.90-11.25)	
Latin American Born, immigrated < 15 years old	1.76 (0.89- 3.48)		3.87 (1.45-10.32)	
Latin American Born, immigrated 15-30 years old	1.19 (0.79- 1.79)		1.66 (0.98-2.18)	
Latin American Born, immigrated 30+ years old (REFERENCE GROUP)	1		1	

Adjusted for age, sex, Hispanic background, income, education, smoking and field center

Table S4. Association of genus level 16S data with obesity, adjusted for age, sex, field center and Hispanic background. Beta represents regression coefficient relating relative abundance with obesity, defined as body mass index (BMI) above 30 kg/m^2 Taxa with relative abundance > 0.01% were included in this analysis.

Taxa (Genus)	Beta	Raw P-value	FDR corrected P-value
Oscillospira	-0.139	<0.001	<0.001
Acidaminococcus	0.318	<0.001	<0.001
Megasphaera	0.277	<0.001	<0.001
Cloacibacillus	-0.11	<0.001	<0.001
Catenibacterium	0.314	<0.001	<0.001
Anaerotruncus	-0.18	<0.001	<0.001
Ruminococcus	-0.147	<0.001	<0.001
Unclassified.Ruminococcaceae	-0.138	<0.001	<0.001
Unclassified.Mogibacteriaceae	-0.188	<0.001	<0.001
Unclassified.Erysipelotrichaceae	-0.383	<0.001	<0.001
Unclassified.Coriobacteriaceae	-0.235	<0.001	<0.001
Unclassified.Clostridiales	-0.207	<0.001	<0.001
Unclassified.Christensenellaceae	-0.426	<0.001	<0.001
Coprobacillus	-0.15	0.001	0.0052857
Prevotella	0.304	0.002	0.0098667
Unclassified.YS2	-0.217	0.004	0.0185
Unclassified.Victivallaceae	-0.144	0.009	0.037
Unclassified.ML615J28	-0.147	0.009	0.037
Streptococcus	0.162	0.011	0.042842
Eggerthella	-0.109	0.012	0.0444
Unclassified.Clostridiaceae	-0.13	0.019	0.066952
Unclassified.Rikenellaceae	-0.148	0.023	0.077217
Blautia	-0.065	0.024	0.077217
Akkermansia	-0.192	0.034	0.10483
Odoribacter	-0.131	0.038	0.11248
Fusobacterium	0.12	0.051	0.14515
Unclassified.Barnesiellaceae	-0.154	0.064	0.17443
Sutterella	0.106	0.066	0.17443
Unclassified.Veillonellaceae	0.111	0.07	0.17862
Lactobacillus	0.115	0.075	0.185
Butyricimonas	-0.116	0.087	0.20768
Faecalibacterium	-0.087	0.101	0.23356
Unclassified.Bacteroidales	-0.092	0.108	0.24218

Holdemania	-0.069	0.12	0.26118
Unclassified.Anaeroplasmataceae	-0.037	0.134	0.28331
Veillonella	0.105	0.142	0.286
Lachnospira	-0.097	0.143	0.286
Dorea	0.045	0.157	0.30169
Lachnobacterium	0.11	0.159	0.30169
Unclassified.Enterobacteriaceae	0.111	0.164	0.3034
Alistipes	-0.069	0.186	0.33571
Megamonas	0.07	0.202	0.3559
Bacteroides	-0.057	0.221	0.38033
Klebsiella	0.057	0.233	0.39186
Roseburia	0.061	0.249	0.40947
Unclassified.Leuconostocaceae	0.038	0.257	0.41343
Anaerostipes	-0.064	0.301	0.47391
Unclassified.Paraprevotellaceae	0.061	0.312	0.481
Adlercreutzia	-0.041	0.347	0.52404
Collinsella	0.054	0.361	0.53428
CF231	0.03	0.377	0.54702
Unclassified.RF39	-0.066	0.389	0.55358
Desulfovibrio	-0.059	0.401	0.55363
Pyramidobacter	-0.024	0.404	0.55363
Unclassified.Elusimicrobiaceae	-0.018	0.459	0.60654
Turicibacter	-0.036	0.459	0.60654
Unclassified.S247	-0.062	0.468	0.60758
Succinivibrio	-0.036	0.494	0.63028
Parabacteroides	-0.025	0.593	0.74376
Phascolarctobacterium	0.038	0.661	0.81523
Coprococcus	-0.013	0.692	0.83222
Bulleidia	-0.015	0.7	0.83222
Eubacterium	-0.025	0.718	0.83222
Bifidobacterium	-0.026	0.72	0.83222
Dialister	0.032	0.731	0.83222
Haemophilus	-0.019	0.792	0.86051
Paraprevotella	0.026	0.795	0.86051
Bilophila	-0.017	0.796	0.86051
Unclassified.RF32	-0.02	0.805	0.86051
Pseudomonas	-0.013	0.814	0.86051
Treponema	-0.003	0.827	0.86194
Clostridium	0.01	0.864	0.888
Unclassified.Lachnospiraceae	0.004	0.88	0.889
Unclassified.Peptostreptococcaceae	-0.009	0.889	0.889

FDR, false discovery rate

Table S5. Association of genus level 16S data with age at relocation among Latin American born individuals, adjusted for age, sex, field center and Hispanic background. Beta represents regression coefficient relating relative abundance with older age at relocation to the mainland US. Taxa with relative abundance > 0.01% were included in this analysis.

Taxa (Genus)	Beta	Raw P-value	FDR corrected P-value
Bacteroides	-0.134	<0.001	<0.001
Acidaminococcus	-0.3	<0.001	<0.001
Unclassified.YS2	0.205	<0.001	<0.001
Unclassified.RF39	0.277	<0.001	<0.001
Unclassified.Clostridiales	0.101	<0.001	<0.001
Sutterella	-0.174	<0.001	<0.001
Holdemania	-0.125	<0.001	<0.001
Butyricimonas	0.186	<0.001	<0.001
Bifidobacterium	-0.218	<0.001	<0.001
Unclassified.Enterobacteriaceae	0.2	0.001	0.005692
Unclassified.Coriobacteriaceae	0.137	0.001	0.005692
Haemophilus	0.181	0.001	0.005692
Collinsella	-0.149	0.001	0.005692
Unclassified.Christensenellaceae	0.158	0.003	0.0111
Phascolarctobacterium	0.194	0.003	0.0111
Oscillospira	-0.071	0.003	0.0111
Odoribacter	0.142	0.003	0.0111
Megasphaera	-0.167	0.003	0.0111
Faecalibacterium	0.12	0.003	0.0111
Blautia	-0.064	0.003	0.0111
Unclassified.Victivallaceae	0.118	0.004	0.014095
Unclassified.Ruminococcaceae	0.067	0.005	0.016818
Parabacteroides	-0.093	0.01	0.030833
Dorea	-0.062	0.01	0.030833
Anaerotruncus	-0.083	0.011	0.03256
Unclassified.S247	0.158	0.013	0.03563
Klebsiella	0.09	0.013	0.03563
Adlercreutzia	-0.079	0.016	0.042286
Desulfovibrio	0.126	0.018	0.045931
Unclassified.Anaeroplasmataceae	0.042	0.022	0.054267
Unclassified.Leuconostocaceae	0.056	0.026	0.062065
Eubacterium	-0.116	0.027	0.062438
Unclassified.Barnesiellaceae	0.132	0.034	0.074
Lachnospira	0.105	0.034	0.074
Lachnobacterium	0.123	0.036	0.076114

Paraprevotella	0.156	0.038	0.078111
Prevotella	0.149	0.042	0.084
Coprobacillus	-0.068	0.046	0.089579
Eggerthella	-0.06	0.069	0.13092
Unclassified.Bacteroidales	0.074	0.082	0.1517
CF231	0.043	0.088	0.15883
Turicibacter	0.06	0.096	0.16914
Unclassified.Clostridiaceae	0.068	0.102	0.17553
Unclassified.ML615J28	0.064	0.129	0.21695
Unclassified.Paraprevotellaceae	0.065	0.15	0.24667
Bulleidia	0.038	0.194	0.30702
Dialister	-0.092	0.195	0.30702
Fusobacterium	-0.058	0.211	0.32529
Akkermansia	0.081	0.236	0.35549
Unclassified.Lachnospiraceae	-0.024	0.242	0.35549
Megamonas	-0.048	0.245	0.35549
Coprococcus	0.028	0.25	0.35577
Unclassified.Erysipelotrichaceae	-0.058	0.266	0.3714
Ruminococcus	0.025	0.295	0.40426
Unclassified.Peptostreptococcaceae	-0.05	0.308	0.4144
Pyramidobacter	0.021	0.321	0.42193
Veillonella	0.053	0.325	0.42193
Treponema	0.01	0.413	0.52693
Cloacibacillus	0.018	0.426	0.53431
Unclassified.Rikenellaceae	0.038	0.439	0.54143
Anaerostipes	-0.034	0.464	0.56289
Pseudomonas	-0.028	0.504	0.60155
Clostridium	0.026	0.534	0.62724
Streptococcus	0.029	0.551	0.63709
Unclassified.Mogibacteriaceae	0.017	0.646	0.73337
Unclassified.Elusimicrobiaceae	-0.008	0.663	0.73337
Alistipes	0.017	0.664	0.73337
Lactobacillus	0.019	0.701	0.76285
Unclassified.RF32	0.021	0.728	0.78075
Succinivibrio	-0.008	0.849	0.89751
Catenibacterium	0.009	0.893	0.93073
Bilophila	0.005	0.929	0.95481
Roseburia	0.002	0.95	0.96301
Unclassified.Veillonellaceae	0	0.993	0.993

FDR, false discovery rate

Table S7. Fungal taxa that differ between US born (USB) and Latin American born (LAB).

All taxa presented met criteria of |LDA score| > 10^2 for comparisons of mainland US born (USB) versus Latin American born (LAB). Bold, |LDA score| > 10^4

	Level	Increased	Relative abundance	Pct. Carriers
**Aspergillaceae**	Family	USB	0.23	88.74
**Eurotiomycetes**	Class	USB	0.24	89.22
**Eurotiales**	Order	USB	0.23	88.92
**Aspergillus**	Genus	USB	0.21	87.37
**Cyberlindnera**	Genus	USB	0.020	11.61
**Cyberlindnera_jadinii**	Species	USB	0.020	10.78
Penicillium_roqueforti	Species	USB	0.0067	4.23
Agaricomycetes	Class	USB	0.013	14.71
Kluyveromyces_marxianus	Species	USB	0.0012	3.10
Panellus	Genus	USB	3.15E-06	0.06
Xenasmatella_ardosiaca	Species	USB	1.47E-06	0.06
Alternaria_rosae	Species	USB	3.96E-06	0.06
Erythricium_laetum	Species	USB	9.68E-05	0.06
Cladosporium_salinae	Species	USB	6.31E-05	0.06
Erythricium	Genus	USB	9.68E-05	0.06
Candida_railenensis	Species	LAB	0.0024	3.34
Cystofilobasidium	Genus	LAB	0.0052	8.76
Dipodascus_geotrichum	Species	LAB	0.014	7.15
**Tremellomycetes**	Class	LAB	0.038	35.85

Table S8. Association of genus level ITS1 data with obesity, adjusted for age, sex, field center and Hispanic background. Beta represents regression coefficient relating relative abundance with obesity, defined as body mass index (BMI) above 30 kg/m^2. Taxa with average relative abundance > 0.01% and presence in at least 5% individuals (i.e. 23 predominant fungal genera) were included in this analysis.

Taxa. ITS genus	Beta	Std. Error	Raw p value	FDR corrected p value
Debaryomyces	-8.09E-03	0.003	0.013	0.299
Guehomyces	3.69E-03	0.002	0.061	0.557
Kluyveromyces	1.51E-03	0.001	0.091	0.557
Cystofilobasidium	-1.67E-03	0.001	0.107	0.557
Candida	1.23E-02	0.008	0.121	0.557
Clavispora	2.23E-03	0.002	0.282	0.818
Mrakia	7.12E-04	0.001	0.310	0.818
Pichia	4.29E-04	0.000	0.312	0.818
Saccharomyces	-8.46E-03	0.009	0.320	0.818
Dipodascus	-1.90E-03	0.002	0.416	0.866
Penicillium	1.28E-03	0.002	0.473	0.866
Wallemia	1.79E-03	0.003	0.501	0.866
Kazachstania	-7.03E-04	0.001	0.538	0.866
Meyerozyma	-6.29E-04	0.001	0.620	0.866
Wickerhamomyces	4.98E-04	0.001	0.622	0.866
Aspergillus	2.91E-03	0.007	0.674	0.866
Hanseniaspora	-4.31E-04	0.001	0.723	0.866
Agaricus	5.59E-04	0.002	0.777	0.866
Cladosporium	-6.12E-05	0.000	0.829	0.866
Trichosporon	-3.69E-04	0.002	0.836	0.866
Rhodotorula	-5.00E-04	0.003	0.845	0.866
Cyberlindnera	4.86E-04	0.003	0.865	0.866
Issatchenkia	-9.30E-05	0.001	0.866	0.866

FDR, false discovery rate

Table S9. Association of genus level ITS1 data with age at relocation among Latin American born individuals, adjusted for age, sex, field center and Hispanic background. Beta represents regression coefficient relating relative abundance with older age at relocation to the mainland US. Taxa with average relative abundance > 0.01% and presence in at least 5% individuals (i.e. 23 predominant fungal genera) were included in this analysis.

Taxa. ITS genus	Beta	Std. Error	Raw p value	FDR corrected p value
Candida	1.84E-02	0.006	0.002	0.046
Cyberlindnera	-5.03E-03	0.002	0.019	0.201
Aspergillus	-1.14E-02	0.005	0.028	0.201
Mrakia	-1.11E-03	0.001	0.035	0.201
Saccharomyces	-1.28E-02	0.006	0.044	0.202
Trichosporon	2.34E-03	0.001	0.079	0.303
Hanseniaspora	1.33E-03	0.001	0.145	0.476
Cladosporium	2.82E-04	0.000	0.183	0.526
Rhodotorula	2.12E-03	0.002	0.269	0.633
Kluyveromyces	7.31E-04	0.001	0.275	0.633
Penicillium	-1.26E-03	0.001	0.345	0.703
Issatchenkia	3.73E-04	0.000	0.367	0.703
Debaryomyces	1.95E-03	0.002	0.423	0.747
Guehomyces	9.79E-04	0.001	0.507	0.747
Wallemia	-1.24E-03	0.002	0.534	0.747
Agaricus	8.91E-04	0.001	0.547	0.747
Clavispora	8.73E-04	0.002	0.574	0.747
Meyerozyma	5.06E-04	0.001	0.593	0.747
Cystofilobasidium	3.88E-04	0.001	0.617	0.747
Pichia	8.74E-05	0.000	0.783	0.883
Dipodascus	4.30E-04	0.002	0.806	0.883
Kazachstania	-1.20E-04	0.001	0.888	0.928
Wickerhamomyces	-1.51E-05	0.001	0.984	0.984

FDR, false discovery rate
