# Supplementary material for: Gut microbiome composition in the Hispanic Community Health Study/Study of Latinos is shaped by geographic relocation, environmental factors, and obesity
Source: Genome Biol. 2019 Nov 1;20:219. doi: 10.1186/s13059-019-1831-z (PMC6824043; doi:10.1186/s13059-019-1831-z)
Supplement: Supplementary file 3 — Additional file 3: Review history. [file 13059_2019_1831_MOESM3_ESM.docx]

Review History

**First round of review**

**Reviewer 1**

**Are you able to assess all statistics in the manuscript, including the appropriateness of statistical tests used?** No, I do not feel adequately qualified to assess the statistics.

**Comments to author:**

In this large sample of Latino cohort participants with fecal sample collection protocols, the authors evaluate associations of participant characteristics with alpha and beta-diversity of the micro/mycobiota and the Prevotella to Bacteroides ratio. Their primary findings focus on place of birth, age at immigration/years in the U.S. Overall strengths of the manuscript include the unique and critically important study population, sample size, multicenter/varied Hispanic origin, and characterization of bacterial and fungal diversity. Overall weaknesses include cross-sectional analysis (cannot be fixed but causes vs. consequences of could both be considered better in discussion), inadequate consideration/treatment of variables like an acculturation multicomponent scale and key dietary factors, and limited focus on overall structure and putative function of bacterial communities (e.g., co-occurrence/co-exclusion).  It is also not quite clear what is really gleaned from the comparison to AGP beyond repeating what other papers in this space have done. While there is certainly value in the a priori analysis, very little new or specific to this population (vs. other immigration papers in non-Hispanics), is learned with the focus on the P to B ratio. For example, one conclusion is that migration to U.S. at an early age results in "loss" of Prevotella and fungal diversity, but what is gained?  Maybe its a range of other bacteria to break down a more diverse diet - and why microbial diversity doesn't follow as strongly with other findings. With more in-depth involvement of cohort investigators with expertise in these areas and those on the manuscript already, this paper could be significantly improved and its importance to public health made much clearer than it is now.  

Other specific comments:

p. 4 The authors aptly present hypotheses and prior literature regarding gut microbiome community structure and adoption of Western-style diet (shifting dietary patterns or nutrition transition from carbohydrate staples to more animal products), but there is very little in the analysis or discussion of findings to support this. What about fiber or varying fiber sources, particularly beans/legumes? It is possible that total fiber and even total fat or protein intake may not differ between the groups of interest (does it?), but one would expect the primary food contributors to fiber intake (and others) do. This pops again on Discussion page 17 line 36, and p 18 lines 24-31, but no context is given from the current analysis. It is unclear why the authors didn't or couldn't look at this. Also, with such a strong focus on two fiber generalists, Prevotella and Bacteroides - that may essentially get the same job done but with different health consequences - the authors should additionally consider Chen Sci Reports Jan 2017 "Fiber-utilizing capacity varies in Prevotella- versus Bacteroides-dominated gut microbiota; and Desai...Sonneburg Cell Nov 2016. This may also account for the lack of associations with obesity. In short, substitution effects (which can be estimated analytically) within the diet and the microbiome are both meaningful.

Questions, which may have answers elsewhere but would be nice to know more readily:

The focus on different age groups (sometimes above 45 and sometimes under 26) look a bit arbitrary and need further justification in the text where they first appear. Are these population-based cutpoints or something else? Its not intuitive on page 29 and 30 either, why exclude vs. stratified analysis to determine if associations differ among these groups (not just masked/confounded).

For findings on page 8 lines 22, does the p-value account for multiple comparisons? Does it assume that all exposure/participant metadata is independent?  

Is sitting time available, not just physical activity (job/recreational)?

How many 24-hour dietary recalls, average/min/max, were collected from participants? No reference is provided for statement on page 21 (lines 29-36). 

Is it possible that age at immigration/other variables are also associated with frequency of returning to Mexico/Latin America post-immigration, maybe even for extended periods of time?

The findings on page 7 are difficult to understand and need some further considerations for clarity, particularly lines 51-53.

Abstract minor word choice/usage: replace "etiological reasons for" with "etiology of"; p.4 "have been little studied"...copy editors should be able to catch the others. 

Other lesser known papers of the microbiome in Mexican Americans that may be of interest/support current findings include Hoffman Plos One April 2018 and Ross Microbiome March 2015.

**Reviewer 2**

**Are you able to assess all statistics in the manuscript, including the appropriateness of statistical tests used?** Yes, and I have assessed the statistics in my report.

**Comments to author:**

Kaplan et al. performed amplicon sequencing of 16S rRNA gene and ITS fragment from fecal samples of 1674 Hispanic / Latinos participants, and report a difference in Prevotella : Bacteroides ratio and in microbial diversity between those who migrated to the US when young (<18 years old) and those who migrated as adults (> 45 years old). The authors' point on migration and westernized microbiota is similar to Vangay et al. 2018 Cell, with a different population. The age difference is broadly consistent with a more plastic microbiota as teenagers/young adults (e.g. twins' age of no longer sharing a household, Xie et al. 2016 Cell Systems). So it is good to see more diverse and young cohorts from around the world. The focus of this story maybe too much on P:B, and differences in other taxa, such as Enterobacteriaceae are not sufficiently covered. Fungal ITS data from such a large cohort is new, but it has surprisingly contributed little to the story, other than diversity.

The impression that Prevotella only eats plants and do not forage on host molecules is based on a very limited number of draft genomes. The dominant Prevotella species in the gut, P. copri, has been implicated in diseases such as rheumatoid arthritis (Scher et al. 2013 eLife, Zhang et al. 2015 NMed) and T2D (Pedersen et al. 2016 Nature). But it is now emerging that people from developing countries have P. copri strains that are absent in the US (De Filippis et al. 2019 Cell Host Microbe and other meeting presentations). So do the authors have access to a few new genomes from this Hispanic population?
Data from hunter-gatherers and apes suggest that Treponema is more for fiber. Do the authors see that as a not so abundant genus? Fig. 2 only shows the top 20.

Fig. 1 shows a difference in PCA relative to US and UK cohorts, but there is no reference for samples from developing countries, ideally from the home countries of these Hispanic / Latino individuals. Technical consistency needs to be controlled for when plotting 16S results from different studies. Unweighted and weighted UniFrac distances would help reveal how the cohorts differ in low-abundance and high-abundance bacteria, respectively. It would also be helpful to color-code some of the metadata onto the plots, e.g. country of origin, age at migration, current BMI.

As Mexico represents the largest source of the cohort, how does the P:B ratio look in this sub-cohort only? As the authors describe, Puerto Rico born individuals migrated young and Cuban born individuals migrated old.

Some of the claims lack statistical rigor. For mining of the dataset by other researchers in the future, both the sequences and the metadata need to be easily accessible.

Other technical or textual comments:
1)     The Abstract should be revised to adhere to the fact that this is a cross-sectional instead of longitudinal comparison.

2)     In the first paragraph, " In fact, many of these, including obesity, diabetes, and asthma are highly prevalent in the US Hispanic population, although the association with the Hispanic GMB pattern is unknown. ", specific citations should be added.

3)     In Figure 2, the q value should be added. In addition, there is a lack of explanation of statistical method in this caption. And the confounders which have been adjusted should be mentioned. The two groups also need to be box-plotted together for comparison, not separately.

4)     The q value is required in Figure 3. Are these independently or cumulatively analysed? It is not known how the factors relate to each other. Some of the top factors on the chart are difficult to interpret. For example, what does 'Health Insurance' mean? Is it reflecting socioeconomic status and occupation? The 16S plot shows 'Years in the mainland US' and 'Age at measure'. Does that mean 'Age at relocation' is not significant? The description in the Results should also be more precise, without unfounded generalization.

5)     Also in Figure 3, 'Hispanic national origin' indeed showed up at the top 1 or 2 for both the 16S and ITS results. But do we know where they stayed in the US and does that location difference matter? Figure 4 only says mainland vs. non-mainland. There could be many combinations.

6)     In Figure 4, q value is required. Many beta 95% CIs are near or containing zero. The effect size is relatively small for this association. 

7)     Potentially influence from medication use, healthy product, antibody, disease status, sequence library, and sequencing amount need to be controlled for in Sup. Table 1 and 2. For Sup. Table 2, the association among obesity, birthplace and age at relocation should be tested to validate the epi results. 

8)     In line 21 of the page 11, the description "Comparisons of GMB metrics among national background groups revealed weak or no associations (Figure 3A and 3B). " seems to be about Figure 4.

**Authors Response**

**Point-by-point responses to the reviewers’ comments:**

Please find below point-by-point response to the comments. All changes to the manuscript are marked in the text.

**Comments from editors:**

*1. Concerns that additional insights into the bacterial and fungal diversity are needed, particularly since this is such a large and unique cohort.*

The recommendation to incorporate additional analyses about the bacterial and fungal components was an extremely helpful point of this general editorial recommendation and specific suggestions of reviewers. We have addressed this point in several ways.

First, in the revised manuscript we provide detailed analysis and discussion of bacterial taxa identified by 16S and fungal taxa identified through ITS1 analysis. This includes identifying taxa that differ among Hispanic population subgroups and also those that are associated with obesity. Please see: **Abstract**; **pages 7-8, 14-15, 20-22** of the revised manuscript; **Tables 1 and 6**; **Supplemental Tables 1, 4, 5, 6, 7, 8 and 9**; **Supplemental Figure 6**. Almost all of this material is new.

In addition, we added original findings and discussion regarding *Prevotella* species that may be associated with Hispanic background, including *P*. *Copri* as recommended by one of the reviewers (see **critique 22**). Third, we addressed the specific comment of Reviewer 2 regarding Enterobacteriacae through additional analyses of this taxa (see **critique 20**).

Lastly, the enigmatic finding of lower bacterial diversity in US-born Latinos is interesting and potentially indicates population differences as does the lack of association of obesity with *Prevotella* : *Bacteroides* ratio.

*2. Reviewer 2 also suggests additional statistical analyses*

We have substantially reanalyzed our data with regard to the statistical concerns of Reviewer 2. Please see **critiques 29, 30** and **34** below which indicate the relevant manuscript changes and additions.

*3. Both reviewers suggest discussion of diet and other possible confounders.*

This has been addressed in several places through additional analysis that consider the effect of confounding variables. All of the main analyses have been substantially re-run, and the results of fully-adjusted multivariable models which control for diet, physical activity, socioeconomic status, medications and other potential confounders have been added (e.g., **Figures 3 and 6**, **Supplemental Figure 4**). We note that none of these analyses changed the main conclusions of the manuscript. The methods, results and interpretation of diet analyses have been expanded in **Tables 3, 4** **and** **5**, **Supplemental Table 2**, and **pages 11-12 and 20** of the manuscript.

**Reviewer #1 comments:**

*4. Overall weaknesses include cross-sectional analysis (cannot be fixed but causes vs. consequences of could both be considered better in discussion)…*

Thank you for raising this valid point. We have added further discussion of the constraints of the study design (**page 25**). The wording of the **abstract** has also been modified to reflect the cross-sectional study design more accurately.

*5. … [Weaknesses include] inadequate consideration/treatment of variables like an acculturation multicomponent scale and key dietary factors*

We augmented our analyses of acculturation related variables. This material appears on **page 10-11** and **Figure 3**, with methods described in detail on **pages 28-29**. Three different approaches were used to characterize each participant’s orientation to cultural features of the Hispanic/Latino community or that of non-Latino/non-Hispanic US mainstream. We have revised the manuscript to provide a more full description of these multi-item scales, which include Spanish or English language use, dietary acculturation, and a social relations component (Short Acculturation Scale for Hispanics or “SASH”, Social Subscale as defined by Marin et al 1987).

Analysis of dietary variables has been considerably expanded in the revised manuscript (see **Tables 3, 4** **and** **5**, **Supplemental Table 2**, and **pages 11-12 and 20**.) As described, analysis of broad categories of food groups suggested that whole grains, vegetables, red and processed meats, and trans fat nutrients were dietary influences on the *Prevotella* : *Bacteroides* ratio. Further analyses (**Table 5**) demonstrate that the US born individuals consumed a diet higher in fats and lower in carbohydrates and fiber, relative to those born in Latin America. Next, we sought to identify the specific food groups that distinguished between groups defined by immigration and birthplace. In the US born individuals, the average daily consumption of fruit and whole grain servings was only half that among those who relocated from Latin America as adults.

Finally, we have added new analyses that consider the confounding effects of diet and acculturation in a more complete and systematic fashion. This includes **Figures 3 and 6**, and **Supplemental Figure 4**.

*6. … [Weaknesses include] limited focus on overall structure and putative function of bacterial communities (e.g., co-occurrence/co-exclusion)*

In response to specific comments of reviewers we have added analyses that go beyond the overall community structure (see **critiques 20, 21, 22, and 23**). We recognize our manuscript has certain limitations (e.g., inability resolve bacterial gene function using 16S data) and these are mentioned in the Discussion (**pages 19, 24**). We constructed co-occurrence networks in exploring our data and found that they did not add much useful interpretation; although this is an interesting suggestion, in the interest of brevity we have chosen to leave those analyses out of the manuscript.

*7. It is also not quite clear what is really gleaned from the comparison to AGP beyond repeating what other papers in this space have done*

We agree that the comparison with American Gut and UK Gut are not central to the conclusions of the paper. This part of the manuscript has been removed. Instead, we cite a prior study (page 5) which draws the same conclusion as ours regarding differences between Hispanics and non-Hispanics (Ross 2015, see **critique 19**).

*8. While there is certainly value in the a priori analysis, very little new or specific to this population (vs. other immigration papers in non-Hispanics), is learned with the focus on the P to B ratio. For example, one conclusion is that migration to U.S. at an early age results in "loss" of Prevotella and fungal diversity, but what is gained? Maybe its a range of other bacteria to break down a more diverse diet - and why microbial diversity doesn't follow as strongly with other findings. With more in-depth involvement of cohort investigators with expertise in these areas and those on the manuscript already, this paper could be significantly improved and its importance to public health made much clearer than it is now.*

We agree with the reviewer that other genera beyond Prevotella and Bacteroides are of potential interest. Please see newly added material that discusses bacterial genera which were associated with both obesity and age at relocation **on pages 7-8, 14-15, 20-22** of the revised manuscript; **Tables 1, 6 and 7**; **Figure 2**; **Supplemental Tables 1, 4, 5, 7 and 8**; **Supplemental Figure 6**.

The reviewer’s point about the need to consider diversity of diet is also important. New material has been added on the Hispanic diet and its association with immigration and obesity (**Tables 3, 4** **and** **5**, **Supplemental Table 2**, and **pages 11-12 and 20**). Please also see **critiques 5 and 10** (variation in diet) and **critique 11** (interaction between diet and bacteria).

With regard to the involvement of cohort investigators, we note that the authors of this manuscript includes the Principal Investigators of the study’s field centers (Kaplan, Schneiderman, Talavera, Daviglus) and Coordinating Center (Sotres-Alvarez), and the gut microbiome laboratories (Knight, Burk). We have added several statements with relevance to public health and disease outcomes that may be related to our microbiome findings (**pages 20-22, 26**).

*9. p. 4 The authors aptly present hypotheses and prior literature regarding gut microbiome community structure and adoption of Western-style diet (shifting dietary patterns or nutrition transition from carbohydrate staples to more animal products), but there is very little in the analysis or discussion of findings to support this.*

We have added data that shed light on the shift in dietary pattern associated with US residence and acculturation. Key parts of the manuscript include **Table 5** and relevant text on **page 12**, showing variation in diet with birthplace and immigration. The discussion has been considerably expanded in its treatment of diet (**pages 18 and 20**). Please also see response to **critique 5**.

*10. What about fiber or varying fiber sources, particularly beans/legumes? It is possible that total fiber and even total fat or protein intake may not differ between the groups of interest (does it?), but one would expect the primary food contributors to fiber intake (and others) do. This pops again on Discussion page 17 line 36, and p 18 lines 24-31, but no context is given from the current analysis. It is unclear why the authors didn't or couldn't look at this.*

The analysis of diet has been expanded to include a larger ranger of nutrients, food groups and specific foods, see **critiques 5 and 9**. As shown in **Table 5**, the US born individuals consumed a diet higher in fats and lower in carbohydrates and fiber. We further explored the detailed food source data (**Supplemental Table 2**) to address the reviewer’s excellent question about consumption of fiber and in particular beans and legumes. Beans and legumes had a mean intake of 1.0 servings per day (standard deviation, 1.9 servings/day), which is high by US standards. For example, well-accepted therapeutic diets such as the Dietary Approaches to Stop Hypertension (DASH) intervention^[1]^ and the Mediterranean diet patterns^[2]^ set goals of one daily serving of these foods, similar to the average level in our cohort. However, we found no significant variation in bean/legume intake according to US born or age-at-relocation groups, and intake of beans and legumes were not associated with microbiome. Instead, fruit and whole grains were the sources of fiber that appeared to differ across the population, favoring the adult-age immigrants to the US who had higher intakes of these foods (**page 12**), and grains in particular were associated with the gut microbiome (**Table 4**).

*11. Also, with such a strong focus on two fiber generalists, Prevotella and Bacteroides - that may essentially get the same job done but with different health consequences - the authors should additionally consider Chen Sci Reports Jan 2017 and Desai Cell Nov 2016. This may also account for the lack of associations with obesity. In short, substitution effects (which can be estimated analytically) within the diet and the microbiome are both meaningful.*

We thank the reviewer for the suggestion to glean insights from these two references. We have added a new discussion point, citing these papers twice (see page **4** and **19-20**). We propose that among those of US mainland birth, relative Bacteroides GMB dominance may influence their response to dietary components and exposure to disease related mechanisms such as short chain fatty acid production and susceptibility to pathogens because of degradation of the GI mucus barrier. With regard to substitution effects, we understand that these are widely applied to diet data but are unsure what particular analysis the reviewer is asking for.

*12. The focus on different age groups (sometimes above 45 and sometimes under 26) look a bit arbitrary and need further justification in the text where they first appear. Are these population-based cutpoints or something else? Its not intuitive on page 29 and 30 either, why exclude vs. stratified analysis to determine if associations differ among these groups (not just masked/confounded).*

We thank the reviewer for drawing attention to our incomplete description of these analyses. We provide a more complete description and rationale for the age groupings that were chosen. For example:

a. The choice of category cutpoints for age as 18 years old, 35 years old, and 45 years old (for example see Figure 4A) followed conventions established in our initial Study of Latino publication (Daviglus et al, JAMA 2012, PMID: 23117778), while also preserving adequate sample size within each category.

b. In the case the age-restricted analysis, we wished to conduct similar analyses of *Prevotella* : *Bacteroides* ratio versus age at relocation, within population subgroups defined by current age. We have revised and expanded our explanation of why individuals who migrated after age 26 were excluded from this analysis. In short, this choice of cutpoint was data-driven; it had to do with allowing us to make a fair comparison across groups defined by attained age. Please see **page 9**, and **Supplemental Figure 3**, which are re-written sections that aim to explain the approach more clearly.

*13. For findings on page 8 lines 22, does the p-value account for multiple comparisons? Does it assume that all exposure/participant metadata is independent?*

We agree with the suggestion to account for multiple testing. We have adjusted this and another other analyses in the manuscript to control the false discovery rate and added a statement to the Methods to describe the method we used. See methodological statement on **page 35**, updates to **Figure 2** **and 3**. We also present a correlation matrix which addresses the question of whether variable inter-correlation might have introduced statistical problems; we conclude that correlations were almost all weak to moderate (**Supplemental Figures 8 and 9**).

*14. Is sitting time available, not just physical activity (job/recreational)?*

As it happens, through a seven day accelerometry protocol we collected standardized measures of sedentary time among participants in our study. As shown in **Table 2** and referenced in the manuscript text on **page 12**, the proportion of participants in the highest quartile of sedentary time was greater for those who relocated to the mainland US after age 45. PERMANOVA (results for top 35 variables shown in **Figure 2**) found that sedentary time parameterized as quartiles was not significantly associated with Bray-Curtis distance for bacterial (r2= 0.0003, q-value= 0.19) or fungal microbiota (r2=0.001, q-value=0.16). This variable did not rank among the top 35 according to r2 value and therefore, was not shown **Figure 2**, although we have made mention in the text of this analysis (**page 12**). We include these data and a discussion point (**pages 22-23**) in the revised manuscript.

*15. How many 24-hour dietary recalls, average/min/max, were collected from participants? No reference is provided for statement on page 21 (lines 29-36).*

Please see the detailed methods for this two-episode 24 hour recall protocol on **page 28**, which have added in the revised version.

*16. Is it possible that age at immigration/other variables are also associated with frequency of returning to Mexico/Latin America post-immigration, maybe even for extended periods of time?*

We have collected this kind of information, and have added the relevant variable to the descriptive table (see **Table 2**). Of note, length of visit to country of origin post-immigration was among the significant correlates of ITS1 beta diversity (**Figure 2**). We were able to add adjustment for return to participants’ home countries as a covariate in the main analyses, which did not alter our findings regarding birthplace or relocation-age (see **Figure 3**).

*17. The findings on page 7 are difficult to understand and need some further considerations for clarity, particularly lines 51-53.*

Thank you, we have rewritten this section.

*18. Abstract minor word choice/usage: replace "etiological reasons for" with "etiology of"; p.4 "have been little studied"*

Thank you, we have made these changes.

*19. Other lesser known papers of the microbiome in Mexican Americans that may be of interest/support current findings include Hoffman Plos One April 2018 and Ross Microbiome March 2015.*

Thank you for the suggestion. We have added Ross et al as an additional reference (**pages 4 and 16, reference 8**), both to point to another well conducted gut microbiome study and also to provide evidence of differences between Latino and non-Latino US groups. It is interesting that the Ross study (Cameron County, Texas) was conducted in a border region, which in comparison with our study likely has quite a distinct migration history and also more frequent exposure to the Mexican environment. We opted not to cite Hoffman et al because it addresses the oral rather than the gut microbiome.

**Reviewer #2 report:**

*20.* The age difference is broadly consistent with a more plastic microbiota as teenagers/young adults (e.g. twins' age of no longer sharing a household, Xie et al. 2016 Cell Systems). So it is good to see more diverse and young cohorts from around the world. *The focus of this story maybe too much on P:B, and differences in other taxa, such as Enterobacteriaceae are not sufficiently covered.*

Thank you for the suggestion. The idea to compare our results with the UK Twins cohort is interesting and we have added this reference (**page 18, reference 23**).

Abundance of *Enterobacteriaceae* showed a qualitative difference between US born and Latin American born individuals (adjusted for gender and field center, p = 0.025, also see **Figure 1**), but did not have graded association with relocation-age or BMI. Additional detail is provided here for the reviewer to see.

*21. Fungal ITS data from such a large cohort is new, but it has surprisingly contributed little to the story, other than diversity.*

Thank you for encouraging us to expand analysis and discussion of fungal ITS1 analysis in the manuscript. Please refer to **pages 7-8, 15, 21-22** of the revised manuscript; **Tables 1, 6 and 7**; **Figure 2**; **Supplemental Tables 6, 7, 8 and 9**. This uncovered several fungi with higher abundance in the Latin American born group (*Rhodotorula mucilaginosa*, several *Candida* species), and some interesting constituents that were higher in the GMB of US born individuals including *Cyberlindnera jadinii*.

*22. The impression that Prevotella only eats plants and do not forage on host molecules is based on a very limited number of draft genomes. The dominant Prevotella species in the gut, P. copri, has been implicated in diseases such as rheumatoid arthritis (Scher et al. 2013 eLife, Zhang et al. 2015 NMed) and T2D (Pedersen et al. 2016 Nature). But it is now emerging that people from developing countries have P. copri strains that are absent in the US (De Filippis et al. 2019 Cell Host Microbe and other meeting presentations). So do the authors have access to a few new genomes from this Hispanic population?*

We appreciate the reviewer’s suggestion. Since we have used 16S V4 data, we extended our analyses beyond the genus level but were cautious to raise caveats about the limits of 16S for resolving down to the species level. Specifically, we went back and using our data find that most (88.7%) *Prevotella* in our cohort maps to *P. copri* as shown below (now included as **Supplemental Table 1**). Based on these data we do not have information on P. copri strains. We have added some relevant information and discussion to the manuscript (**page 7, 19**).

*23. Data from hunter-gatherers and apes suggest that Treponema is more for fiber. Do the authors see that as a not so abundant genus? Fig. 2 only shows the top 20.*

We appreciate the importance of this comment. The average relative abundance for *Treponema* is only 0.02%, and we find 13 Treponema carriers defined as those with relative abundance > 0.01% (Table below). All but one are Latin American born. It is of possible interest that among *Treponema* carriers, South Americans seem to be over-represented (3/13, 23%), while Puerto Rican, Dominican and Cuban (e.g., Caribbean) individuals may be under-represented (only 1 of each among the 13 persons). We considered this to be insufficient data to add to the paper due to the small sample. We provide the table below given the reviewer’s interest, which details the 13 *Treponema* carriers. Depending on the advice of the editors we will be happy to add this as a supplemental table.

Legend: S, San Diego; C, Chicago, B; Bronx; M, Miami. Age_immi, age at relocation to the mainland US

*24. Fig. 1 shows a difference in PCA relative to US and UK cohorts, but there is no reference for samples from developing countries, ideally from the home countries of these Hispanic / Latino individuals. Technical consistency needs to be controlled for when plotting 16S results from different studies….*

Given the valid concerns raised by Reviewer #2, as well as the advice of Reviewer #1 that the comparison versus American Gut and UK Gut data do not add to the paper, we have elected to drop Figure 1. Instead, we cite Ross et al which provided a similar type of analysis already.

*25. As Mexico represents the largest source of the cohort, how does the P:B ratio look in this sub-cohort only?*

We have added the suggested analyses among the Mexican group (**Supplemental Figure 4**).

*26. Some of the claims lack statistical rigor. For mining of the dataset by other researchers in the future, both the sequences and the metadata need to be easily accessible.*

We refer the reviewer to the “Data and biospecimen availability and access” section of the manuscript which describes how microbial sequences and metadata can be accessed in accordance with relevant data security procedures. We have addressed all the specific statistical questions in the revised version; thank you for providing these suggested modifications to the statistical plan.

*27. The Abstract should be revised to adhere to the fact that this is a cross-sectional instead of longitudinal comparison.*

We thank the reviewer for the opportunity to be more clear about the limitations of the study design. The abstract has been updated accordingly.

*28. In the first paragraph, " In fact, many of these, including obesity, diabetes, and asthma are highly prevalent in the US Hispanic population, although the association with the Hispanic GMB pattern is unknown. ", specific citations should be added.*

Appropriate citations have been added as suggested. See **page 4, references 2 and 3**.

*29. In Figure 2, the q value should be added. In addition, there is a lack of explanation of statistical method in this caption. And the confounders which have been adjusted should be mentioned. The two groups also need to be box-plotted together for comparison, not separately.*

Thank you for these suggestions to improve the clarity of this plot. We tried alternative layouts and found that this organization of **Figure 1** provided the clearest readability. The caption clarifies the method as unadjusted Wilcoxon signed-rank test.

*30. The q value is required in Figure 3. Are these independently or cumulatively analysed? It is not known how the factors relate to each other.*

Thank you for this comment. We have updated **Figure 2** (formerly Figure 3) to reflect q-values. We have also updated the figure legend to state that these variables were assessed individually in univariate analysis (also see **page 8**). We have also added a **Supplementary Figures 8 and 9** and discussion on **page 33** which shows the correlation between the variables depicted in Figure 2 for the reference of readers.

*31. Some of the top factors on the chart are difficult to interpret. For example, what does 'Health Insurance' mean? Is it reflecting socioeconomic status and occupation? The 16S plot shows 'Years in the mainland US' and 'Age at measure'. Does that mean 'Age at relocation' is not significant? The description in the Results should also be more precise, without unfounded generalization.*

We appreciate the opportunity to clarify the meaning of variables included in Figure 2. Methods for defining health insurance have been added to **page 28**, along with other details that had been omitted for brevity (**pages 28-29**).

We also respond to this comment by explaining to the reader how we produced the ranking of variables (by r2 values) in Bray-Curtis distance analysis in **Figure 2**. As may be expected, different approaches to parameterizing the variables produces slightly different results. In screening a large set of variables for their r2 value, we present analyses that were based on an ordinal analysis approach for all variables. That is, linear ordination of variable categories was incorporated; thus, this test was most sensitive to dose-response relationships between levels of the explanatory variable, and Bray-Curtis distance. When 16S analyses were re-run using a test for global differences among categories without assuming a dose-response ordination, age at relocation appeared as among the most important and significant variables. This is consistent with the pattern of other results, for example as demonstrated in **Figure 3A**, which shows the association of the birthplace/age at relocation variable with bacterial Shannon diversity to have a “V” shape rather than a linear association. We hope this explanation will help the reader to gain a more nuanced understanding of our approach and the results. These additional details are provided on **page 34** (Methods).

*32. Also in Figure 3, 'Hispanic national origin' indeed showed up at the top 1 or 2 for both the 16S and ITS results. But do we know where they stayed in the US and does that location difference matter? Figure 4 only says mainland vs. non-mainland. There could be many combinations.*

While we recorded place of birth (city, state, country) and place of current residence, we do not have complete information on all of the places in US where participants may have stayed over time. We relied upon the field center at which they enrolled as a participant to capture the place of residence at the time of sampling. While we note that regression analyses adjust for field center of residence, we have also noted as a limitation of the study that complete residential histories were not available and introduce an unmeasured dimension of complexity (**page 25**).

*33. In Figure 4, q value is required. Many beta 95% CIs are near or containing zero. The effect size is relatively small for this association.*

We have updated confidence intervals in **Figure 3** (formerly Figure 4) to account for multiple testing. Regarding the issue of effect sizes, we recognize that the scaling of some of the independent variables such as Short Acculturation Scale for Hispanics (SASH) acculturation score led to very small effect sizes that are difficult to interpret. We provide an alternative coding of this variable as categories in order to describe the strength of association across the range of acculturation.

*34. Potentially influence from medication use, healthy product, antib[iotics], disease status, sequence library, and sequencing amount need to be controlled for in Sup. Table 1 and 2.*

We controlled for technical details of sequencing (e.g., read depth) by performing rarefaction to 10,000 reads per sample. In addition, to account for other potential confounders identified by the reviewer, we have added the variables mentioned as covariates to regression models shown (**Figures 3 and 6**, **Supplemental Figure 4**, with the method described on **page 34-35**). We note that these additional confounder adjustments did not change our conclusion

*35. For Sup. Table 2, the association among obesity, birthplace and age at relocation should be tested to validate the epi results.*

Confirming our previous publication (**reference 17**), within the gut microbiome study sample obesity was higher among those born in the US and those who relocated to the US at younger ages. We have added a table describing these associations in more detail (**Supplemental Table 3**, and **Supplemental Figure 5**).

*36. In line 21 of the page 11, the description "Comparisons of GMB metrics among national background groups revealed weak or no associations (Figure 3A and 3B). " seems to be about Figure 4.*

Thank you. This has been corrected.

**Second round of review**

**Reviewer 1**

The authors have made significant efforts to improve the manuscript and to share more analytic findings from this unique cohort. I have no further comments on content. 

When editing for clarity, I would just caution the authors/copy editors to carefully consider the descriptions of results for this epidemiologic study keeping in mind that directionality or causality is lacking.

**Reviewer 2**

All comments were answered satisfactorily and the manuscript is much improved in analyses and in writing.

Yet the following minor points remain to be fixed.
1. "long US exposure" or "More US exposure" used in this study at multiple places (e.g. page 9, Fig. 5) are easily confused with the length of immigration time. Unification for "earlier life exposure" is more appropriate.

2. Fig. 4 "For example, individuals who had migrated at age 50 could only have been represented in the 55-64 years old (blue) or 65+ years old (magenta) " is difficult to interpret. It seems to be "who had migrated for 50 years". Also, the p-values are needed to add at each panel of the graph in Fig. 4.

3. Check all the figures and make them tidy and beautiful. There are many problems such as the irrelevant content in Fig. 1 (facet names) and Fig. 3B (arrow at upper bound of confidence interval), the inappropriate color matching in Fig. 3A. Besides, Fig. 5 is difficult to interpret.

**Authors Response**

We have responded to comments of reviewers: causal language has been corrected, for example we edited the text to include terms such as “has an association with” rather than “has an effect on” (R1); language pertaining to “early life exposure” has been unified (in place of “long US exposure” or “more US exposure” (R2); the legend to Fig. 4 has been corrected to improve clarity, although we have used somewhat different language than that suggested by R2; we have fixed either figures or figure legends to respond to suggestions of R2.

1. ^Moore TJ et al. Effect of dietary patterns on ambulatory blood pressure : results from the Dietary Approaches to Stop Hypertension (DASH) Trial. Hypertension. 1999 Sep;34(3):472-7.^
2. ^Doménech M et al. Mediterranean diet reduces 24-hour ambulatory blood pressure, blood glucose, and lipids: one-year randomized, clinical trial. Hypertension 2014;64:69–76.^
